# Targeting endoplasmic reticulum export disrupts metabolic resilience in multiple myeloma

**DOI:** 10.1038/s41392-026-02833-y

**Published:** 2026-07-07

**Authors:** Utku Horzum, Herbert Oberacher, Margot Haun, Stephan Geley, Monica Roman-Trufero, Holger Auner, Agnieszka Martowicz, Gerold Untergasser, Eberhard Gunsilius, Wolfgang Willenbacher, Hamdullah Yanik, Gunes Esendagli, Dominik Wolf, Hesso Farhan

**Affiliations:** 1 Institute of Pathophysiology, Medical University of Innsbruck, Innsbruck, Austria; 2 Institute of Legal Medicine, Medical University of Innsbruck, Innsbruck, Austria; 3 Division of Hematology, Lausanne University Hospital, Lausanne, Switzerland; 4 Department of Hematology and Oncology, Internal Medicine V, Comprehensive Cancer Center Innsbruck (CCCI), Austrian Comprehensive Cancer Network (ACCN), Tyrolean Cancer Research Institute (TKFI), Medical University of Innsbruck, Innsbruck, Austria; 5 Department of Basic Oncology, Cancer Institute, Hacettepe University, Ankara, Turkey

**Keywords:** Cell biology, Cancer

## Abstract

Multiple myeloma (MM) is characterized by the production and secretion of large quantities of immunoglobulins, making this malignancy highly dependent on mechanisms that maintain cellular proteostasis. While significant clinical progress has been made by targeting the degradative branch of proteostasis, much less attention has been given to the biosynthetic branch. In this study, we demonstrated that inhibiting COPII-dependent endoplasmic reticulum (ER) export induces cell death in several MM cell lines and primary patient-derived cells. The induction of cell death was dependent on the secretory status of MM cells. Blocking ER export in secretory MM cells caused the accumulation of misfolded proteins, which activated ER-associated degradation (ERAD). Consequently, we observed an ERAD-dependent increase in the levels of free cytosolic amino acids and a subsequent activation of mTORC1 signaling. Simultaneously, we observed mitochondrial dysfunction. These alterations resulted in a mismatch between the increased energy demand due to mTORC1 activation, and the disrupted energy supply from mitochondrial impairment. This energetic imbalance results in homeostatic collapse and cell death of secretory MM cells. The therapeutic potential of the concept was demonstrated in two in vivo myeloma models. These findings suggest that the ER export machinery could be a promising therapeutic target in multiple myeloma.

## Introduction

Multiple myeloma (MM) is the second most common hematologic malignancy, arising from a clonal proliferation of plasma cells in the bone marrow. Multiple therapeutic agents have been introduced to treat MM patients,^1,2^ but despite major therapeutic advances, MM remains an incurable disease, and the occurrence of drug resistance with the concomitant disease recurrence represents a major challenge in daily clinical practice. Therefore, there is an ongoing need for new therapeutic strategies for MM. A hallmark of MM cells is the secretion of large amounts of immunoglobulins or free light chains,^3^ thus, making these cells addicted to mechanisms of protein homeostasis (proteostasis). Proteasome inhibitors, a major therapeutic line in MM, target the degradative branch of proteostasis, but to the best of our knowledge, only scant information is available on the role of the biosynthetic branch of proteostasis, i.e., the secretory pathway in MM. Efforts so far have been limited to targeting protein synthesis and quality control,^4,5^ but we completely lack understanding of the role of the machinery that controls export from the endoplasmic reticulum (ER) in the survival of multiple myeloma cells. Selective targeting of immunoglobulin secretion may alter the survival of myeloma cells and sensitize them to other proteostasis-targeting compounds, such as proteasome inhibitors.

Secretory proteins leave the ER in a COPII-dependent manner at so-called ER exit sites (ERES).^6,7^ Among the subunits of the COPII coat, SEC24 is the primary component responsible for secretory cargo binding. Humans express four SEC24 paralogs (SEC24A-D) which have partially overlapping cargo preferences.^8,9^ However, it remains unclear which SEC24 paralog mediates the export of immunoglobulins from the ER and this study aimed at closing this knowledge gap. Here, we identified SEC24A and SEC24B as the paralogs mainly responsible for controlling ER export of immunoglobulins and demonstrate that targeting ER export represents a potential novel therapeutic target in MM. We showed that inhibition of ER export in MM cell lines and patient-derived cells induced cell death, which was driven by the accumulation of misfolded proteins in the ER. Degradation of these misfolded proteins resulted in elevated levels of free cytosolic amino acids, which triggered mTORC1 signaling. Concomitantly, disruption of mitochondrial function due to altered ER homeostasis, resulted in an imbalance between mTORC1-driven metabolic demand, and reduced ATP production by dysfunctional mitochondria. Thus, we show that targeting ER export promotes MM cell death through metabolic imbalance, paving the way for the future development of novel drugs to target MM.

## Results

### SEC24A and SEC24B are important players in multiple myeloma

**Fig. 1:**
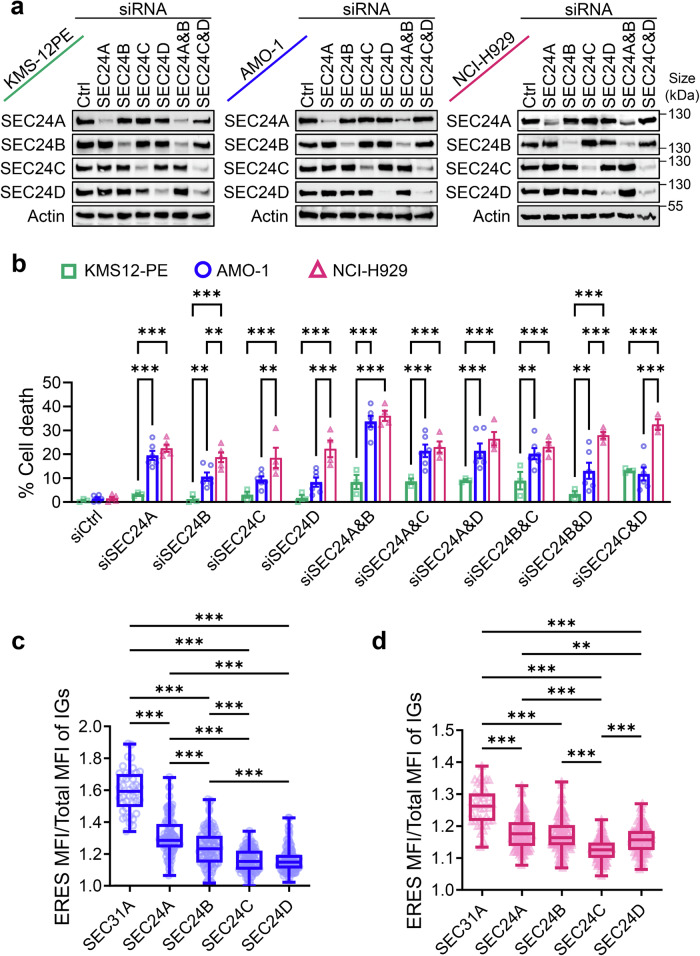
Effect of SEC24 paralog depletion on survival of myeloma cells. **a** Immunoblots validating individual and combinatorial siRNA knockdowns of SEC24 paralogs in KMS12-PE, AMO-1, and NCI-H929 MM cell lines at 48 hafter transfection. **b** The impact of SEC24 depletion on cell viability was assessed using the CellTiterGlo assay after 48 h of knockdowns. Data were generated in ≥3 independent experiments. Each symbol represents an independent experiment. Significance was tested using the Tukey multiple comparison test (2-way ANOVA, ***P < 0.001; **P < 0.01). **c**, **d** Immunoglobulins predominantly colocalized with SEC24A and SEC24B paralogs. **c** AMO-1 or **d** NCI-H929 cells were incubated at 10 °C for 60 min to increase the local concentration of Igs at ERES. Cells were then spun onto coverslips and processed for immunofluorescence staining using antibodies against SEC24 paralogs and IgA. SEC31A staining was used as a positive control representing almost all ERES. The mean fluorescence intensity of IgA was measured at ERES and normalized to total cytosolic IgA intensity. The results are representative of three biologically independent experiments (number of cells >100 for each condition). Data are shown as a box-and-whisker plot displaying the median, first and third quartiles, and all data points. Whiskers in the box plots indicate maximum and minimum values. Significance was tested using the Tukey multiple comparison test (1-way ANOVA, ***P < 0.001; **P < 0.01)

We first sought to determine which SEC24 paralog contributes to the survival of MM cells. To this end, we initially used three different myeloma cell lines that represent different states of proteostatic burden: (i) non-secretory KMS12PE cells,^10^ (ii) AMO-1 cells, which secretes IgA at low level, and (iii) the NCI-H929 cells, which also secretes IgA, but at a much higher level (Supplementary Fig. 1a, and Supplementary Fig. 2a, b). KMS12PE cells were validated to be non-secretory because no immunoglobulins could be detected by either immunofluorescence or FACS (Supplementary Fig. 2a, b). Because myeloma cells are notoriously difficult to transfect, we first developed a novel protocol for reproducible and efficient siRNA-mediated knockdowns (see Materials and methods, Fig. 1a and Supplementary Fig. 1b, c). Knockdowns of individual SEC24 paralogs or combinations thereof were established and cell viability was assessed 48 after siRNA transfection using the CellTiter-Glo assay. The secretory cell lines NCI-H929 and AMO-1 exhibited pronounced sensitivity to depletion of various SEC24 paralogs (Fig. 1b), whereas survival of non-secretory KMS-12PE cells was unaffected by inhibition of ER export. In particular, the co-depletion of SEC24A&B induced the strongest effects, and was therefore used for the rest of the study as the main perturbation. We detected cleavage of caspase 3 at 48 h after siRNA transfection, and the effect continued to increase up to 96 h after knockdown (Supplementary Fig. 1d). Identical kinetics were observed with PARP cleavage (Supplementary Fig. 1d). Depletion of SEC24A&B induced a significant reduction in steady-state immunoglobulin secretion in both AMO-1 and NCI-H929 cells (Supplementary Fig. 1e, f). For technical reasons, we were unable to perform siRNA rescue experiments, due to the high levels of cell death induced by plasmid transfection in cells that received siRNAs. This was not limited to SEC24 paralog knockdown, but was also observed in control siRNA conditions, indicating that this type of experiment is not compatible with our experimental system.

We next tested the effect of SEC24A&B co-depletion on six additional myeloma cell lines and observed variable levels of reductions in survival (Supplementary Fig. 1g, Supplementary Fig. 2a, b, and Supplementary Table 1). Effects on cell viability were in the range of 20–35%, which is consistent with the notion that silencing SEC24A&B only partially impairs exit from the ER (because cells still have two other Sec24 paralogs), thus representing a mild perturbation of ER-proteostasis (Supplementary Fig. 1e, f). SEC24A&B knockdown also had no effect on general organization of ERES (Supplementary Fig. 3), indicating that this alteration of ER export might be selective to certain secretory proteins, and will not affect general secretion. The absence of an effect in non-secretory MM cells is in line with previous reports showing that knockdown of COPII subunits in the non-secretory cell line HeLa had no effect on their survival.^11^ Finally, we investigated whether inhibition of ER-export synergizes with the proteasome inhibitors, including bortezomib, carfilzomib, and MG132. Because both interventions target separate branches of the proteostasis network, we assessed their combined effect in a panel of 8 secretory myeloma cell lines and the non-secretory KMS-12PE cells as a negative control. As expected, non-secretory KMS12PE cells showed very little sensitivity to all proteasome inhibitors, which could not be enhanced by knockdown of SEC24A&B (Supplementary Figs. 4–6). In contrast, high level of toxicity was observed in the hypersecretory NCI-H929 cell line, supporting the notion that the secretory status determines the sensitivity to proteostasis-disrupting conditions. This high level of proteasomal toxicity prevented us from testing a synergism with SEC24A&B depletion in this cell line (Supplementary Figs. 4, 5, and Supplementary Fig. 6). However, in all cell lines, we observed additive effects to various extent and clear synergism of proteasome inhibitors with SEC24A&B knockdown in AMO-1 cells (Supplementary Figs. 4–6). On the contrary, the combination of SEC24A&B knockdown with lenalidomide, a commonly used IMiD, did not result in major additive or even synergistic effects in most cell lines (Supplementary Fig. 7).

### Immunoglobulins colocalize with SEC24A and SEC24B in ERES

The effect of SEC24A&B depletion on survival of secretory myeloma cells implies that these SEC24 paralogs play a dominant role in the trafficking of immunoglobulins out of the ER. While previous work showed that ER-export of IgM is controlled by COPII-dependent factors,^12,13^ the identity of the SEC24 paralog mainly responsible was not investigated. We performed colocalization experiments of immunoglobulins with SEC24 paralogs in ERES. There is currently no evidence that SEC24 paralogs segregate on specific ERES. Rather, some ERES have a dominant SEC24 paralog, but are generally thought to be positive for all of them. Exit from the ER is fast, which precludes the detection of immunoglobulins in ERES at steady-state. To overcome this difficulty, we cultured AMO-1 and NCI-H929 cells for 60 min at 10 °C, a condition known to slow the exit of secretory proteins at the level of ERES.^14,15^ Cells were subsequently fixed and co-immunostained for IgA together with the individual SEC24 paralogs. Although immunoglobulins colocalized with all SEC24 paralogs, the colocalization was significantly stronger with SEC24A and SEC24B (Fig. 1c, d and Supplementary Fig. 8). This result is in line with the higher sensitivity of myeloma cells to SEC24A&B depletion compared to the other paralogs. We also co-stained immunoglobulins with SEC31, a component of every COPII coat and found that it showed greater colocalization with immunoglobulins than with any SEC24 paralog, indicating some level of segregation of SEC24 paralogs to different ERES.

### ER stress in cells depleted of SEC24A&B does not contribute to cell death

**Fig. 2:**
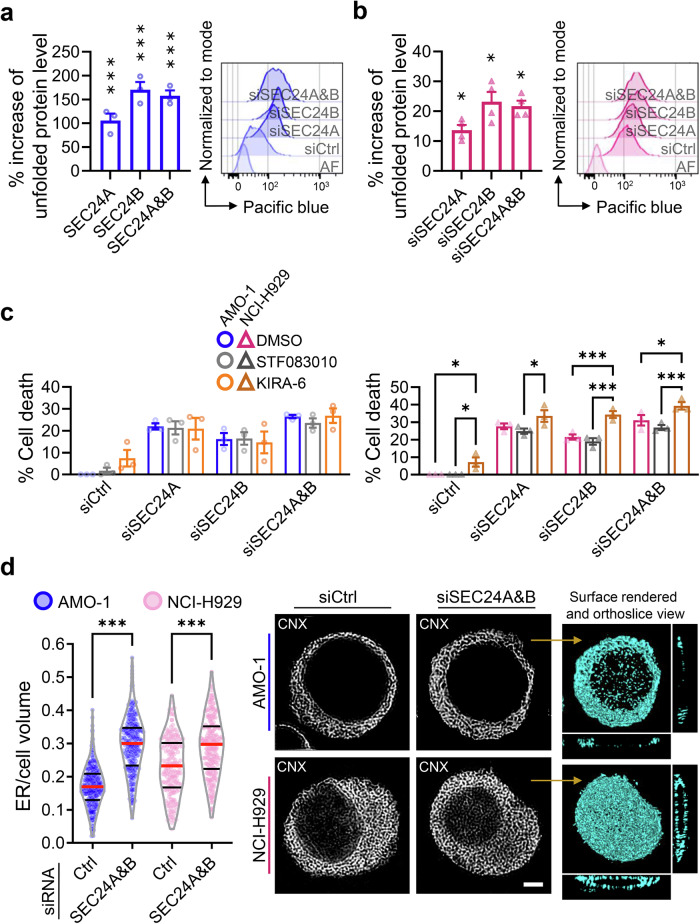
SEC24A&B-depleted secretory MM cells accumulate misfolded proteins and increase the size of the ER. **a**, **b** TPE-MI fluorescence was measured by flow cytometry after staining cells with 50 µM TPE-MI for 30 min. The data show the percentage change in median fluorescence intensity compared to the siRNA control condition for **a** AMO-1 and **b** NCI-H929 cells. Means ± SEM are shown (*n* = 3–5 measurements). Statistical significance was measured with one-way ANOVA followed by Dunnett’s test (***P <0.001; *P < 0.05). Representative histograms are shown on the right side of each graph with the same color code. **c** After 32 h of siRNA-mediated transfection of MM cell lines, cells were treated with vehicle (DMSO), 2 µM KIRA6, or 40 µM STF083010 for an additional 16 h. Cell viability was then assessed using the CellTiter-Glo assay. Data represent the mean of three independent experiments ±SEM. ***P < 0.001; *P <0.05, as calculated using a two-way ANOVA and Tukey post hoc test. **d** After 48 h of SEC24A&B co-knockdown, cells were stained with calnexin to label the ER, and confocal images of 3D reconstructions of the ER were acquired. Image stacks with 0.1 μm steps along the z-axis were used to analyze ER morphology using ImageJ 3D-suite. ER volume was normalized to the corresponding cell volume. Quantification was performed from three independent experiments. Data are presented as violin plots. Horizontal solid red lines in the violin plots represent medians, and black lines represent the first (lower) and third (upper) quartiles. Dots represent individual cells, AMO-1 (blue) and NCI-H929 (pink), *n* > 50 for each condition. The right panel shows a representative set of images for each cell type, with AMO-1 images presented at the top and NCI-H929 images below. Scale bar, 10 µm

To elucidate the mechanisms mediating myeloma cell death induced by knockdown of SEC24A&B, we hypothesized that reduced ER export activity leads to ER stress due to an accumulation of secretory proteins in the ER lumen, triggering an excessive unfolded protein response (UPR). In fact, crowding the ER lumen was previously shown to induce ER stress in non-secretory cell types.^16,17^ To test this hypothesis, we determined whether SEC24A&B depletion results in an increase in the load of misfolded proteins. We employed TPE-MI, a compound which upon reaction with free SH-groups in unfolded proteins becomes fluorescent, and thus can be quantified by flow cytometry.^18^ As shown in Fig. 2a and b, depletion of SEC24A&B (individually and in combination) resulted in a marked increase in the level of misfolded proteins in the secretory myeloma cell lines AMO-1 and NCI-H929. The increase with SEC24B depletion alone was already as strong as with the double knockdown, and was slightly higher than SEC24A depletion. This likely reflects a compensatory effect, because the levels of SEC24B are slightly increased in SEC24A knockdown cells, while SEC24B depletion does not affect SEC24A levels (Fig. 1a and Supplementary Fig. 1b). However, despite this increase in misfolded proteins, only a very weak UPR was detected in SEC24A&B knockdown cells (Supplementary Fig. 9a, b), suggesting that the observed cell death is unlikely to be mediated by UPR activation. In fact, the IRE1-XBP1 branch of the UPR has been reported to exert essential homeostatic roles in plasma cell differentiation and immunoglobulin production.^19–21^ In line with this homeostatic role of IRE1, pharmacological inhibition of IRE1 kinase or RNase activities did not rescue SEC24A&B depleted myeloma cells (Fig. 2c). One of the pro-survival roles of IRE1 is that it plays a role in expansion of ER volume in cells facing a high proteostatic burden. Silencing SEC24A&B resulted in an expansion of ER volume in the secretory myeloma cell lines AMO-1, FOLE and NCI-H929 (Fig. 2d, and Supplementary Fig. 10), but not in the non-secretory KMS12-PE cell line (Supplementary Fig. 10a) or low secretory cell line OH-2 (Supplementary Fig. 10b). ER expansion was completely dependent on IRE1 activity in the highly secretory NCI-H929 cell line (Supplementary Fig. 10d), and partially IRE1-dependent in the moderate secretory cell line AMO-1 (Supplementary Fig. 10c). Overall, these results indicate that the induction of UPR is unlikely to be involved in the cell death of SEC24A&B depleted myeloma cells.

### An ERAD-mTORC1 signaling pathway mediates cell death of SEC24A&B-silenced myeloma cells

**Fig. 3:**
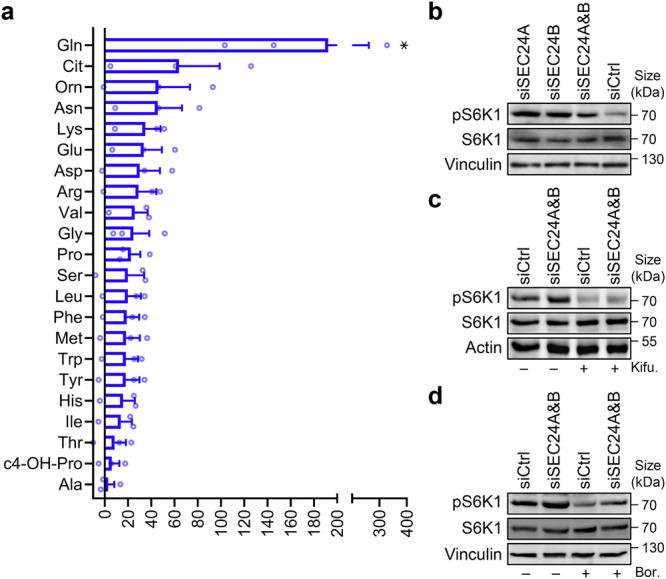
SEC24A&B-depleted cells exhibit elevated amino acids and ERAD-dependent mTORC1 activation. **a** Intracellular amino acid levels are presented as a percentage change over the negative control samples after 48 h of SEC24A&B silencing in AMO-1 cells. Each bar graph represents the average percentage change ±SEM (*n* = 3) relative to negative control cells. Statistical significance was determined by one-way ANOVA followed by Dunnett’s test (*P = 0.05). **b** AMO-1 cells were transfected with the indicated SEC24 siRNAs or control siRNA and subjected to western blot analysis of phosphorylated and total S6K1 after 48 h of knockdown. **c** AMO-1 cells transfected with siCtrl or siSEC24A&B were treated with 2 μM of kifunensine for 16 h, **d** or with 200 nM of bortezomib for 4 h, and then subjected to immunoblotting of phosphorylated and total S6K1 at the end of the 48-h knockdown. **b**–**d** Data are representative of at least three independent experiments

The accumulation of misfolded proteins in SEC24A&B knockdown cells prompted us to investigate a potential involvement of ER associated degradation (ERAD) in myeloma cell death. Induction of ERAD results in targeting misfolded proteins for proteasomal degradation, which would consequently elevate cytosolic amino acids. Therefore, we prepared cytosolic extracts of AMO-1 cells comparing control and SEC24A&B depleted cells and measured the levels of free amino acids. We normalized the amino acid levels to total cellular protein content. This normalization method is adequate because we did not observe an increase of cell size in SEC24 knockdown conditions (Supplementary Fig. 11). Impairment of ER-export resulted in a broad ~15–45% increase of most amino acids as well as a ~ 200% increase of glutamine (Fig. 3a, Supplementary Fig. 12, Supplementary Table S3–4). We performed dedicated analysis of glutamine levels in AMO-1 and NCI-H929 cells. SEC24A&B depletion resulted in a strong increase (~50% in AMO-1 cells and >200% in NCI-H929 cells) (Supplementary Fig. 13). This increase was likely due to an induction of ERAD, because it could be fully reversed by the ERAD-inhibitor kifunensine, as well as by proteasomal inhibitor bortezomib in both cell lines (Supplementary Fig. 13). This indicates that ERAD is a major contributor to the increased glutamine levels in SEC24A&B knockdown cells. Measurements of the levels of cytosolic amino acids was repeated in NCI-H929 cells and show a similar result as obtained with AMO-1 cells, namely that knockdown of SEC24A&B increases the levels of amino acids in the cytosol. To test whether the increase in amino acid levels is mediated by ERAD, we treated cells with kifunensine and observed that this reversed the increase, indicating a major role for ERAD in mediating the increase of cytosolic amino acids in SEC24A&B silenced cells (Supplementary FigS. 14–15).

Mammalian target of rapamycin complex 1 (mTORC1) senses nutrient availability and is activated by a wide range of amino acids.^22,23^ Accordingly, we found that silencing SEC24A&B resulted in higher levels of phospho-S6 kinase (Fig. 3b, Supplementary Fig. 16a), a surrogate marker for mTORC1 activation. Treatment of SEC24A&B silenced cells with the ERAD inhibitor kifunensine, abolished the increase in mTORC1 (Fig. 3c and Supplementary Fig. 16b), indicating that mTORC1 activation depends on ERAD-mediated proteolysis. Likewise, brief (4 h) treatment with low doses of (200 nM) of bortezomib also reduced mTORC1 activation in SEC24A&B knockdown cells (Fig. 3d and Supplementary Fig. 16c). Consistent with ERAD activation in SEC24A&B knockdown conditions, kifunensine treatment of AMO-1 and NCI-H929 cells reduced polyubiquitinated protein levels to almost baseline levels (Supplementary Fig. 17a, b).

**Fig. 4:**
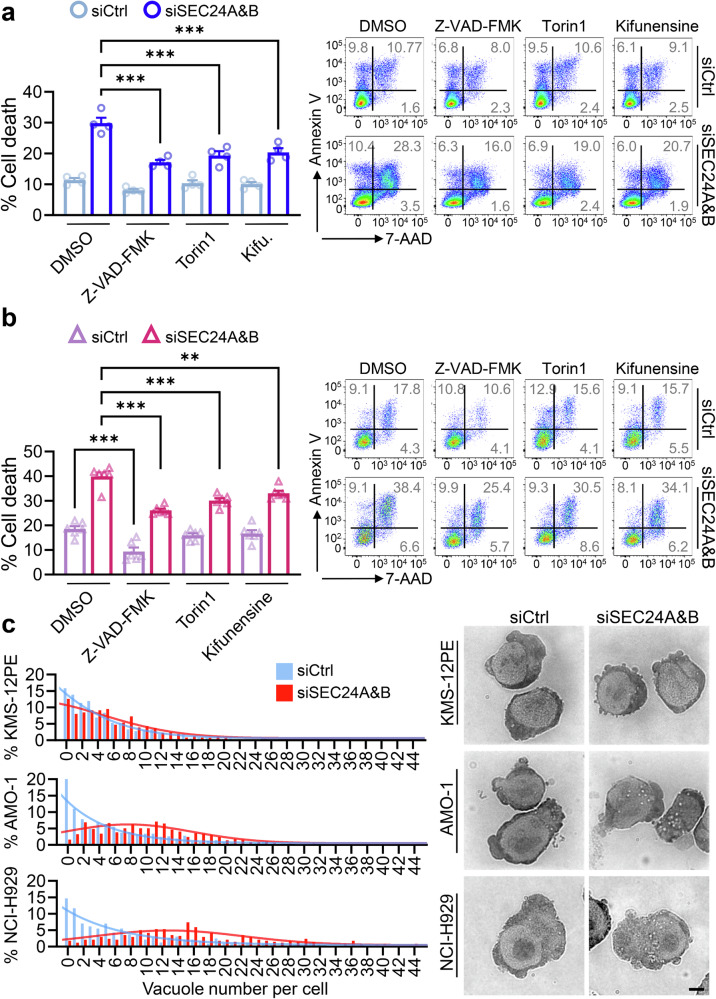
Mechanisms and types of cell death of SEC24A&B depleted cells. **a** AMO-1 and **b** NCI-H929 cells were exposed to Z-VAD-FMK (20 μM), torin-1 (2 nM), kifunensine (2 μM), or DMSO for the last 16 h of a total 48-h SEC24A&B or control siRNA transfection, after which cell death was evaluated using an Annexin V and 7-AAD staining assay followed by flow cytometry analysis. The data are presented as means ± SEM from 4 to 6 individual experiments. Statistical significance was tested using the Tukey multiple comparison test (1-way ANOVA, ***P < 0.001; **P < 0.01; *P < 0.05). Right panels show representative dot plots of Annexin V and 7-AAD staining in MM cells. **c** Histograms show the distribution of the number of vacuoles per cell after SEC24A&B or control siRNA transfection for 48 h in MM cell lines. The number of vacuoles was quantified in more than 350 cells for each cell type. Data were obtained from at least three independent experiments. Right panels show representative images of MM cells after May-Grunwald/Giemsa staining. Scale bar, 10 µm

Our data so far support a model whereby alteration of ER-export triggers an accumulation of misfolded proteins in the ER, which are degraded by ERAD, resulting in an increase in the levels of cytosolic amino acids, which triggers mTORC1 activation. Next, we addressed two questions: (i) is elevated mTORC1 activity connected to myeloma cell death in SEC24A&B silenced cells, and (ii) what type of cell death is induced? To address these questions, we treated SEC24A&B silenced cells with torin-1 (to block mTOR) and with kifunensine (to block ERAD) for 16 h and found that both conditions partially rescued AMO-1 and NCI-H929 cells from cell death (Fig. 4a, b). The torin-1 concentration used (2 nM) reduced hyperactivated mTORC1 signaling to a level comparable to control cells (Supplementary Fig. 18a). This result indicates that elevated mTORC1 activity as well as ERAD are at least partially connected to cell death of myeloma cells with deficient ER-export. Notably, a pan-Caspase inhibitor or a caspase-9 specific inhibitor provided only partial protection (Fig. 4a, b, Supplementary Fig. 18b, c), indicating that apoptosis is not the sole mechanism of cell death induced by SEC24A&B depletion. To explore alternative cell death pathways, we treated SEC24A&B knockdown cells with the ferroptosis inhibitor ferrostatin-1 and necroptosis inhibitor necrostatin-1, neither of which provided protection (Supplementary Fig. 19a, b). Likewise, we found no evidence for senescence induction (Supplementary Fig. 19c). We noted that SEC24A&B knockdown cells exhibited more cytoplasmic vacuolization, which was absent in the non-secretory cell line KMS-12PE (Fig. 4c). Increased ER volume and cytosolic vacuoles are indicators of the induction of paraptosis, a relatively poorly understood cell death mechanism.^24^ In summary, our results indicate that impairment of ER export triggers an ERAD-mTORC1 cascade that reduces the viability of secretory MM cells. Cell death in this context involves both apoptotic and paraptotic mechanisms.

### SEC24A&B depletion in MM cells disturbs the cellular energy balance

**Fig. 5:**
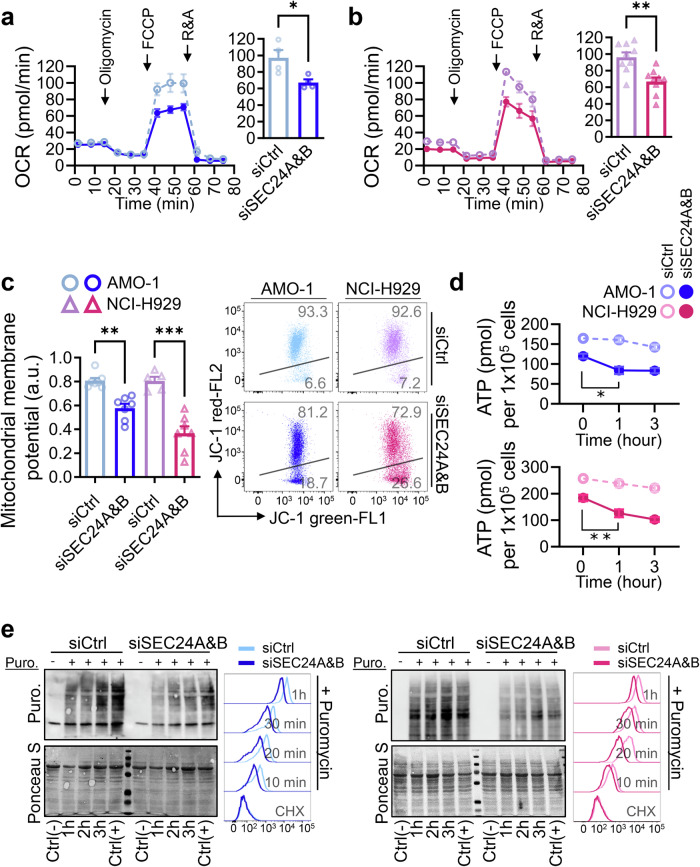
SEC24A&B depleted cells have altered mitochondrial function and exhibited reduced protein synthesis rates. **a**, **b** After 48 h of control or SEC24A&B siRNA-mediated transfection, OCR was measured on a Seahorse XF96 Analyzer under basal conditions and during successive additions of 1.5 mM oligomycin, 0.5 mM FCCP, and 0.5 mM rotenone/antimycin A in **a** AMO-1 and **b** NCI-H929 MM cell lines. Spare respiratory capacity is also presented in the bar graph. Graphs show mean values ± SEM (*n* = 4 independent experiments). Statistical analysis was conducted using a two-tailed unpaired t-test (**P < 0.01; *P < 0.05). **c** Mitochondrial membrane potential was determined by staining with the ratiometric dye JC-1 in AMO-1 and NCI-H929 MM cell lines after 48 h of control or SEC24A&B siRNA-mediated knockdowns. Graphs show mean values ± SEM (*n* = 5–7 independent experiments). ***P < 0.001; **P < 0.01, Student’s unpaired two-tailed t-test. Pseudocolor dot plots show a representative experiment. Red JC-1 staining indicates polarized mitochondria, while a loss of red fluorescence shows depolarization. Mitochondrial depolarization is calculated and presented as Arbitrary Units (a.u.) of the red/green fluorescence intensity ratio. **d** Intracellular ATP levels were measured in control and SEC24A&B-depleted AMO-1 (top panel, blue) and NCI-H929 (bottom panel, pink) cell lines, which were cultured for the indicated time periods in a medium where glucose was replaced with galactose. Lighter dashed lines and solid lines represent control siRNA and SEC24A&B siRNA transfections, respectively. Quantification is shown as mean ± SEM, *n* = 6, two-way ANOVA with Tukey’s multiple comparisons test (**P < 0.01; *P < 0.05). **e** Translation was monitored using puromycin-labeled proteins. Representative results from *n* = 3 experiments are shown. Puromycin incorporation of mock-treated (ctrl(-)), CHX-pretreated (25 μM), and puromycin (puro)-labeled (10 μg/ml) for the indicated times (1 h, 2 h, and 3 h), or only labeled with puromycin without CHX pretreatment for 3 h as a positive control (ctrl(+)), were analyzed by western blot using an antibody to puromycin in AMO-1 (left) and NCI-H929 (right) cells after 48 h of siRNA-mediated knockdown. Anti-puromycin immunoblots (upper panels) and Ponceau S staining (lower panels) are shown. Ponceau blots verify protein loading. FACS analysis of puromycin-labeled cells was detected with Alexa 647–labeled antibodies to puromycin at early time points. Representative histogram overlays are presented next to the blots. Light lines and solid lines represent control siRNA and SEC24A&B siRNA transfections, respectively

Higher mTORC1 activity is typically associated with an anabolic state, where cells generate more biomass through increasing the rate of translation. Because translation consumes ~30% of cellular energy, SEC24A&B silenced cells are expected to have a higher energetic demand. At the same time, disruption of ER homeostasis was shown to compromise mitochondrial function,^25^ suggesting a potential mismatch between energy demand and production. To assess mitochondrial activity, we measured glycolytic and respiratory activity in SEC24A&B knockdown cells. While no effect on basal glycolysis was detected (Supplementary Fig. 20), our data revealed a marked decrease in the spare respiratory capacity in SEC24A&B-depleted MM cells (Fig. 5a, b). Furthermore, we found that SEC24A&B depletion led to significant depolarization of mitochondria that decreases the capacity of secretory MM cells to produce ATP through oxidative phosphorylation at the inner mitochondrial membrane (Fig. 5c). To further assess mitochondrial dysfunction, we shifted the growth conditions of MM cells from glucose-containing to galactose-containing media. The oxidation of galactose to pyruvate by glycolysis yields no net ATP, forcing cells to rely on mitochondrial respiration for ATP generation. In control cells, ATP levels slowly declined over a 3-hour period, whereas SEC24A&B depleted cells exhibited a substantially greater drop in ATP levels, which is consistent with mitochondrial dysfunction (Fig. 5d).

Given that translation is the most energetically costly process,^26^ we asked whether the elevated mTORC1 activity in SEC24A&B knockdown cells would still be able to increase their translation activity, despite having dysfunctional mitochondria. Therefore, we monitored translation using a puromycin-incorporation assay (SUnSET).^27^ FACS was used to detect translation at early time points, while immunoblotting was performed after one hour of puromycin-incorporation. Both approaches revealed a decrease in global translation in SEC24A&B-depleted secretory MM cells, suggesting that energy insufficiency constrains anabolic activity (Fig. 5e). Altogether, these results suggest that impairment of ER export in secretory MM cells disrupts cellular energy balance by simultaneously increasing energetic demand through mTORC1 activation and impairing mitochondrial ATP production. The resulting energetic imbalance eventually leads to cell death of myeloma cells.

### Effect of SEC24A&B depletion on MM cell growth in vivo and in patient-derived cells

**Fig. 6:**
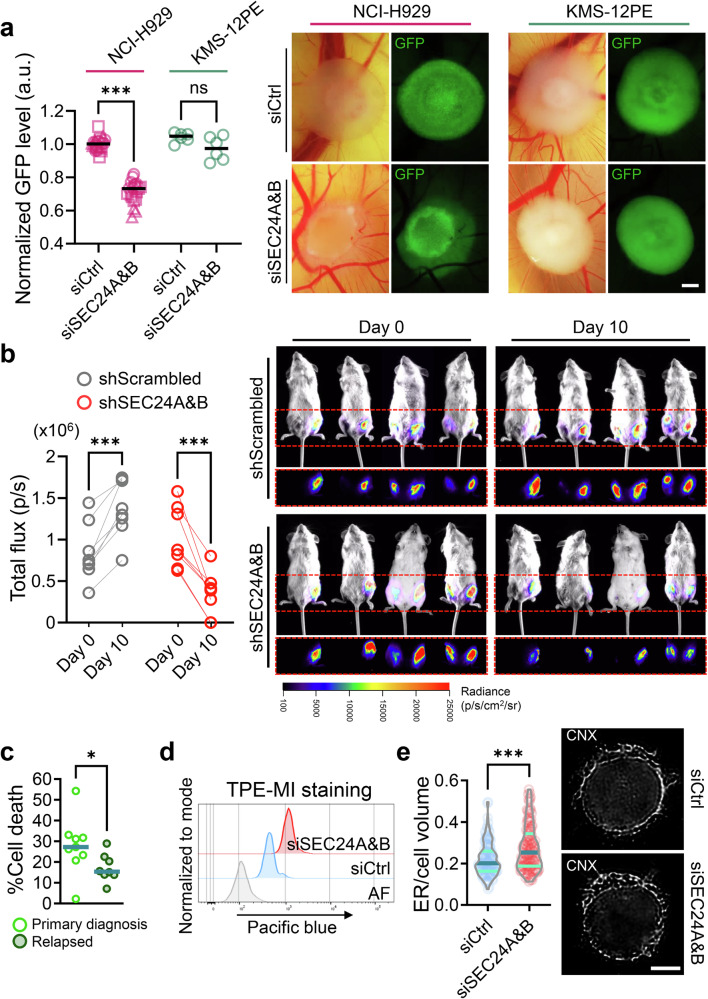
Effects of SEC24A&B knockdown on MM cells in vivo and in patient-derived MM cells. **a** Chicken chorioallantoic membrane assay was used for in vivo testing. MM spheroids containing SEC24A&B or control siRNA treated MM cells in a growth factor-reduced Matrigel matrix were positioned on the membrane and incubated for 4 days. GFP concentrations of single spheroid were measured by ELISA. Quantification of the data was obtained from multiple technical replicates represented as different symbols. Horizontal black lines indicate medians. Statistical analysis of the results was performed by unpaired, two-tailed t test. ***P < 0.001. MM spheroids were photographed using a stereo-fluorescence microscope. Representative spheroids are shown on the right side. Scale bar, 1 mm. **b** NSG mice were intrafemurally injected with NCI-H929 cells bearing scrambled-shRNA or SEC24A&B-shRNA. Two weeks after inoculation, tumors were established in all mice and readily detected by bioluminescence images (day 0). Then, mice received 2.5 mg/kg doxycycline intraperitoneally twice at 5-day intervals. Following two rounds of doxycycline induction, the mice were injected i.p. with 100 μl of CycLuc1 (5 mM) and imaged ten minutes post-injection (day 10). Each point represents the total flux (photons/sec) in the region of interest (red dashed-line) of the mice. Gray circles, NCI-H929 cells bearing scrambled-shRNA (*n* = 8); red circles, NCI-H929 cells bearing SEC24A&B-shRNA mice (*n* = 8). ***P < 0.001, Student’s paired two-tailed t-test. Representative bioluminescence images of mice show systemic tumor progression and response to SEC24A&B depletion. **c** Cell viability of primary human bone marrow CD138^+^ myeloma cells was measured using the CellTiterGlo assay after 48 h of SEC24A&B knockdown. Cell viability was normalized to siRNA control. Patients were grouped as newly diagnosed (*n* = 9) and relapsed (*n* = 8). Lines indicate medians. *P < 0.05, Student’s unpaired two-tailed t-test. **d** Flow cytometry histograms of TPE-MI staining in one representative patient after 48 h of siRNA-mediated knockdowns. AF = unstained cells for autofluorescence. **e** ER volume of one representative patient after 48 h of SEC24A&B or negative control siRNA treatment of patient-derived CD138 + MM cells. ER volume was normalized to corresponding cell volume. Data are presented as violin plots. Horizontal dark cyan lines in the violin plots represent medians, and light cyan lines represent the first (lower) and third (upper) quartiles. Each light-colored circle represents an individual cell (*n* > 100 cells per condition). ***P < 0.001, Student’s unpaired two-tailed t-test. The right panel shows a representative set of images for each condition. Scale bar, 10 µm

Our experiments described so far were conducted in vitro using MM cell lines. To test the growth of MM cells ex vivo, we used a chorioallantoic membrane (CAM) assay as described previously.^28^ We engineered NCI-H929 or KMS-12PE to express GFP-CaaX to enable the visualization and quantification of spheroid growth. The CAM assay offers the advantage of a rapid 3D in vivo system to monitor growth of MM cells. Cells were transfected with non-targeting control siRNA or SEC24A&B siRNAs and tumor spheroids were embedded in growth factor-reduced Matrigel matrix and positioned onto the CAM for 4 days. MM spheroids were excised and their mass was quantified using an anti-GFP-ELISA. SEC24A&B depletion significantly reduced spheroid mass of secretory MM cell line, NCI-H929 (Fig. 6a). In contrast, spheroids derived from non-secretory KMS-12PE cells did not exhibit any significant decrease in size when depleted of SEC24A&B.

To further validate these findings in a mammalian in vivo disease model, we established an orthotopic mouse model using luciferase expressing NCI-H929 cells stably expressing doxycycline-inducible shRNAs targeting SEC24A and SEC24B or a scrambled control. Cells were injected intrafemorally into 6–8-week-old immunocompromised NSG mice to establish orthotopic MM xenograft model in the bone marrow. Two weeks after intrafemoral injection, tumor growth was monitored by bioluminescence imaging and doxycycline was administered intra-peritoneally twice at 5-day intervals. While tumors derived from cells expressing scrambled shRNA continued to grow for the next 10 days, tumors established with SEC24A&B shRNA-expressing cells exhibited a substantial reduction in size (Fig. 6b, Supplementary Fig. 21a, b). Finally, to determine the clinical significance of our findings, we asked whether reduction of SEC24A&B levels in patient-derived myeloma cells would reproduce some of the effects observed with MM cell lines. Primary cells were obtained from 17 patients (9 primary diagnosed and 8 relapsed, Supplementary Table 2). SEC24A&B knockdown resulted in a 30% reduction in the viability of cells isolated from newly diagnosed patients, whereas it had a much weaker effect on cells collected from relapsed patients (Fig. 6c). In line with the observations from cell lines, SEC24A&B silencing also increased the amount of protein misfolding and increased ER volume in primary MM cells (Fig. 6d, e).

## Discussion

A hallmark of MM cells is the production and secretion of vast amounts of immunoglobulins, thus making proteostasis a potential Achilles heel in secretory MM. With the notable exception of proteasome inhibitors, only few efforts have been dedicated to targeting the proteostasis network in MM cells. Proteasome inhibitors target the degradative branch of proteostasis, and have proven to be clinically highly successful.^29–31^ Another example for a role of the degradative branch of proteostasis comes from preclinical studies highlighting the role of autophagy in MM pathophysiology.^32,33^ Only a small number of studies so far have focused on the biosynthetic branch of proteostasis. For instance, targeting the protein synthesis machinery was recently shown to potentiate the activity of the anti-MM drug selinexor.^34^ Targeting oncogenic translation programs was also shown to be effective in targeting myeloma cells.^35^ Another example is the translocon (SEC61) inhibitor mycolactone, which was shown to be effective as a potential anti-MM agent.^4^ The translocon is responsible for the insertion of secretory proteins into the ER lumen and consequently this strategy is likely to unselectively block the secretion of most secretory proteins. Very recently, we showed that drugs targeting the tyrosine kinase LTK affect general ER export, impairs the biogenesis of ER exit sites and reduces the growth of myeloma cells.^36,37^ However, it remained elusive whether targeting the ER export machinery per se can be used to prevent growth of myeloma cells. In the current work, we showed that targeting SEC24A and Sec24B reduces the growth of myeloma cells in vitro and in vivo, without affecting general secretion, because we did not observe an impairment of ER exit sites. Silencing SEC24A&B also had no effect on the growth of non-secretory cells. Future validation will need to include humanized animal models to further connect preclinical results with potential patient trials. To our knowledge, this study provides the first demonstration that targeting the COPII-machinery compromises the survival of both myeloma cell lines and patient-derived cells, suggesting it could be a potential therapeutic option for MM in the future.

Due to the inherent error rate of protein synthesis, a molecule the size of an immunoglobulin will have a 10–15% chance of being misfolded.^38,39^ Although MM cells have secretory apparatus adapted to handle high secretory load, the capacity of the ER to process proteins remains limited.^40^ We observed that hypersecretory MM cells accumulate misfolded proteins when ER-export is inhibited by SEC24A&B knockdown. However, we detected only a mild UPR, which is most likely due to the fact that secretory myeloma cells have an expanded ER, capable of handling the extra burden of misfolded proteins through ERAD.

Our work uncovered an unanticipated mechanistic link between ERAD and mTORC1 in MM cells with altered ER export. Activation of mTORC1 was driven by ERAD-mediated degradation of misfolded proteins, which increased cytosolic amino acid levels. While most amino acids increased by 15–30%, glutamine increased by about 200%. To address its source, we measured glutamine levels upon pharmacological blockade of ERAD (kifunensine) and of the proteasome (bortezomib). Both interventions strongly suppressed the glutamine increase induced by SEC24A&B depletion, indicating that the surge in glutamine is highly dependent on the ERAD pathway (Supplementary Fig. 13). Given that glutamine accounts for only 5.6% and 4.3% of immunoglobulin light and heavy chains, additional mechanisms likely contribute, potentially including altered mitochondrial function and reduced glutaminase activity.

Blocking mTORC1 hyperactivation prevented the death of SEC24A&B depleted MM cells. Although the canonical role of mTORC1 is to mediate cell growth,^41^ there is precedence linking mTORC1 overactivation to cell death, via mechanisms including senescence or autophagy.^42–59^ However, we found no evidence for an induction of senescence or autophagy in SEC24A&B silenced myeloma cells (Supplementary Fig. 22 and Supplementary Fig. 19c). We propose a new model that involves metabolic/energetic imbalance due to a mismatch between energy demand and production. This model is supported by the following observations: (i) protein synthesis, an energy demanding process is downregulated despite active mTORC1, (ii) mitochondria exhibit several signs of being dysfunctional in SEC24A&B depleted cells, and (iii) in accordance with mitochondrial dysfunction, ATP production rapidly declined in SEC24A&B depleted cells upon switch from glucose to galactose. These observations support the notion that deficiency in ER export creates an energetic mismatch: mTORC1-driven anabolic demand exceeds the diminished energy-producing capacity of dysfunctional mitochondria. Although the basis for this mitochondrial impairment remains unclear, we speculate that it might be due to a defect in inter-organelle communication as shown recently by others.^25^ Elucidating this inter-organelle crosstalk is an exciting opportunity for future work.

Overall, our work provides the first evidence that disruption of ER-export *via* SEC24A&B depletion reduces the survival of MM cells in vitro and in vivo by triggering energetic imbalance through a novel ERAD-mTORC1 connection (Supplementary Fig. 23). We establish SEC24A and SEC24B as central nodes linking ER-proteostasis, nutrient sensing, and cellular energy metabolism. Targeting this pathway could therefore represent a novel therapeutic strategy for MM in the future. Currently, there are no drugs that reduce the levels of SEC24A or SEC24B or completely block their function. Future strategies could include the development of PROTACs or chemical glues that promote degradation of SEC24A&B. This could represent a major advance compared to a general inhibition of ER export as is the case with inhibitors of LTK, which we showed recently to induce myeloma cell death.^36^

## Materials and methods

### Cell culture and transfection

Human MM cell lines KMS-12PE, AMO-1, and NCI-H929 were cultured in RPMI1640 supplemented with 10% fetal bovine serum, 2 mM L-glutamine, 100 mg/mL penicillin and streptomycin 100 U/mL and incubated in a humidified atmosphere (5% CO_2_, 37 °C). Further details on cell culture and transfection are provided in the Supplement. Work with patient derived cells was approved by the ethics committee of the Medical University of Innsbruck (Nr: 1198/2021). Further details on isolation of cells are provided in Supplementary methods.

### Immunofluorescence microscopy

Cytospin samples were fixed with 4% paraformaldehyde, permeabilized in 0.1% Triton-X 100, immunostained as detailed in the Supplementary Methods and imaged using a Nikon Spinning Disk Eclipse Ti2 confocal microscope. Details on image analysis and a list of antibodies used in this study is provided in Supplemental Methods.

### Flow cytometry

Flow cytometry data were acquired by BD Fortessa flow cytometer and analyzed by FlowJo software. Details on TPE-MI staining are provided in Supplemental Methods.

### Quantitative analysis of amino acids and biogenic amines

AMO-1 cells transfected with siRNAs for 48 h, or treated with cycloheximide (25 ug/mL) for 4 h as a positive control. Protocols of cell collection and Liquid chromatography-tandem mass spectrometry are provided in Supplemental Methods.

### Chick embryo chorioallantoic membrane (CAM) assay

CAM of fertilized White Leghorn chicken eggs was used mainly following the protocol published by Martowicz^28^ and details are provided in Supplemental Methods.

### Intrafemoral multiple myeloma model in NSG mice

All animal procedures were approved by Animal Care and Use Committee of Kobay Laboratory [Ethical approval no: 778] and were performed in accordance with national and institutional ethical guidelines. Inducible shRNA-expressing NCI-H929 cells were injected intrafemorally into NOD.Cg-*Prkdc*^*scid*^ *Il2rg*^*tm1Wjl*^/SzJ (NSG) mice to establish an orthotopic multiple myeloma model. After tumor establishment, shRNA expression was induced by doxycycline treatment, and disease progression was monitored through bioluminescent imaging following CycLuc1 administration. Details are provided in Supplemental Methods.

## Supplementary information

Supplementary table S3 and S4

Supplementary Material

## Data Availability

All data are publicly available in the main text/supplementary materials.
