# Supplementary material for: Targeting endoplasmic reticulum export disrupts metabolic resilience in multiple myeloma
Source: Signal Transduct Target Ther. 2026 Jul 7;11:262. doi: 10.1038/s41392-026-02833-y (PMC13338250; doi:10.1038/s41392-026-02833-y)
Supplement: Supplementary file 2 — Supplementary Material [file 41392_2026_2833_MOESM2_ESM.docx]

Supplementary Materials for

Targeting ER export disrupts metabolic resilience in multiple myeloma

Utku Horzum, Herbert Oberacher, Margot Haun, Stephan Geley, Monica Roman-Trufero, Holger Auner, Agnieszka Martowicz, Gerold Untergasser, Eberhard Gunsilius, Wolfgang Willenbacher, Hamdullah Yanik, Gunes Esendagli, Dominik Wolf, Hesso Farhan

Correspondence to: [hesso.farhan@i-med.ac.at](mailto:hesso.farhan@i-med.ac.at) , [utku.horzum@i-med.ac.at](mailto:utku.horzum@i-med.ac.at)

**This PDF file includes:**

Materials and Methods

Figures. S1 to S23

Tables S1 to S2

Uncropped images of western blots

Key resources

Extended statistics

Materials and Methods

**Cell culture and transfection**

Human multiple myeloma (MM) cell lines KMS-12PE, AMO-1, and NCI-H929 were cultured in RPMI-1640 supplemented with 10% fetal bovine serum (FBS), 2 mM L-glutamine, 100 mg/mL penicillin and streptomycin 100 U/mL. The other 6 MM cell lines were cultured under the following conditions: URVIN in RPMI-1640 supplemented with 15% FBS and 1 ng/mL interleukin-6 (IL-6); FOLE in RPMI-1640 with 15% human serum (HS); OH-2 and KJON in RPMI-1640 containing 15% HS and 1 ng/mL IL-6; and VOLIN in RPMI-1640 with 15% FBS. All cells were maintained in a humidified incubator at 37°C with 5% CO₂. Cells were passaged at a seeding density of 5 × 10^5^ cells/mL every 3–4 days. The passage routine is critical for MM cell lines. Cells that have been passaged many times and in an irregular manner tend to change their growth behavior and morphology, and are less susceptible to transfection. The day prior to transfection, cell media were refreshed to provide optimal gas exchange and logarithmic growth of cells. On day of transfection, cells were washed twice in RPMI media and resuspended in the same serum free media at a concentration of 2.0 x 10^5^ cells in 50 μL RPMI. After 25 min, serum-starved cells were transferred to the wells of 96-well flat bottom plate, and gently shaken for uniform distribution. After an extra 5 min for placing of cells to the surface of well, transfection complexes (900 ng siRNA in 50 uL RPMI containing 6 uL HiPerfect reagent) were gently added from one side of the well onto the cells. Plate was centrifuged at 30xg for 30 min at RT with slow acceleration/deceleration in a benchtop centrifuge with swinging bucket rotor and plate carriers. Following centrifugation, 200 uL of fresh growth media were immediately added to each wells. This brings the final siRNA concentration to 200 nM. Then, cells were incubated in their standard growth conditions (37°C, 5% CO2). The experiments were performed 48 h after the transfection. The transfection efficiencies were greater than 70% based on the mRNA and protein levels of target genes. The synchronization of cargo traffic in the early secretory pathway by blocking membrane traffic from the ER, temperature block, was performed at 10°C for 1 hour using a heat block. The cells were then applied to slides by cytospin for 3 min at 50xg and for 1 min at 150xg in a pre-cooled centrifuge. Same centrifugation values were used to prepare all cytospun MM samples for immunofluorescence analysis. To assess cell viability and ATP levels, we used the ATP-based CellTiter-Glo Luminescent Cell Viability assay according to manufacturer’s instructions. Senescence was evaluated using the Beta-Glo Assay System according to the manufacturer’s instructions. Glutamine levels were measured using the Glutamine/Glutamate-Glo Assay according to the manufacturer’s instructions. Cellular metabolic rates were measured using a Seahorse XF96 Analyzer, with the Mito Stress Test Kit and the Glycolytic Rate Assay Kit, following the manufacturer's recommendations and necessary optimizations. MM cells infected with the cell membrane marker, GFP-CaaX, were used in *ex ovo* experiments. To generate stable cell line, lentiviral infection of MM cells was performed. For lentivirus production, HEK293T cells were transiently transfected with a lentiviral GFP-CaaX vector together with pCMV-VSV-G and psPAX2 in a respective ratio of 2:1:1. PEI was used as a transfection agent. The media on the HEK293T cells was replaced with fresh media 8–16 hours post transfection. After cells were cultured for 2 days, the supernatant was collected and passed through a 0.45-mm syringe filter unit. MM cells were infected with lentivirus containing cell culture supernatant and 8 mg/mL polybrene. GFP-positive cells were isolated using BD FACS Aria-III cell sorter. SEC24A&B knockdowns were also achieved using RNA interference in NCI-H929 cell line. Briefly, complementary shRNA oligonucleotides directed against SEC24A or SEC24B were designed, annealed and cloned into the pENTR-THTIII vector. Sequence-verified THT-shRNA cassettes were recombined into the GATEWAY-based lentiviral doxycycline-regulated conditional RNAi vector pGLTR-X Puro or GFP. Lentiviral particles were produced by the same way using HEK293T cells. Infected NCI-H929 cells were selected in two steps with puromycin for SEC24A and sorted based on GFP for SEC24B vice versa. Scrambled shRNA was used as the non-targeting control. Successful knockdowns of SEC24A and SEC24B were confirmed by Western blotting. In parallel, shRNA-expressing NCI-H929 cells were also infected with a luciferase reporter vector followed by a selection with hygromycin as previously described above.

**Immunofluorescence microscopy**

Cytospin samples were fixed with 4% paraformaldehyde (PFA) for 15 min at room temperature. Next, cells were washed twice with PBS and permeabilized in 0.1% Triton-X 100 solution for 3 min at room temperature. Afterwards, samples were rinsed twice with PBS, and blocked using 3% BSA in PBS for 1 hour at RT. After blocking nonspecific binding sites, samples were incubated with the primary antibodies diluted in PBS with 3% BSA for 1 hour at RT. Samples were then washed with PBS three times and incubated with florescence-conjugated secondary antibodies diluted in PBS with 3% BSA for 1 hour at RT. After washing three times with PBS, slides were mounted on glass coverslips using polyvinyl alcohol. To assess vacuolization, cytospun samples were stained with 10% May-Grunwald Giemsa. To assess the secretory load of multiple myeloma cells, steady-state cells were cytospun, stained with an anti–human immunoglobulin G, A, and M (H+L) antibody conjugated to FITC, and counterstained with DAPI to visualize nuclei. All images were acquired with a Nikon Spinning Disk Eclipse Ti2 confocal microscope equipped with a 60x oil immersion objective.

**Western blotting**

MM cell lines were transfected with siRNAs against SEC24A and/or SEC24B or negative control (as described above). Drugs or corresponding amounts of DMSO treatments were performed overnight at standard growth conditions. Protein lysates were extracted from the MM cell lines at 48 h after transfection. Cells were washed with ice-cold PBS and homogenized with lysis buffer (150 mM NaCl, 1% NP-40, 0.5% sodium deoxycholate, 0.1% sodium dodecyl sulfate, 50 mM Tris-HCl (pH 8), and 1 mM PMSF supplemented with protease and phosphatase inhibitor cocktail) for 30 min at 4ºC. Crude extracts were then centrifuged at 20,000xg at 4ºC for 15 min to remove cell debris, and proteins in the supernatant were quantified using the Bradford assay. The supernatants were mixed with Laemmli reducing buffer and further denatured at 95ºC for 5 min. Equal amount of proteins were loaded and resolved on 10% SDS-PAGE for nearly all analyses except LC3 which was separated on 15% gels. After electrophoretic separation, proteins were transferred to nitrocellulose membranes and blocked with 5% skim milk in TBS-Tween 20 at RT for 1 hour. Membranes were then incubated with primary antibodies overnight at 4ºC. HRP-conjugated secondary antibodies were applied to the membranes for 1 hour at RT. We detected puromycin incorporation by immunoblotting with the 2A4 monoclonal antibody to puromycin. Cells were pretreated with cycloheximide (25 μM) for 10 min in culture conditions and washed twice with fresh growth media. Then, cells were labeled with 10 μg/ml of puromycin for indicated times at their standard culture conditions. Puromycin incorporation into cells detected by anti-puromycin blot or flow cytometry. Immunoblots were visualized and recorded using enhanced chemiluminescence reagent and ChemiDot imaging system.

**Flow Cytometry**

Flow cytometry data were acquired by BD Fortessa flow cytometer and analyzed by FlowJo software. To measure misfolded protein level, cells were treated with TPE-MI (50 μM in PBS) for 30 min at 37 °C and then washed and resuspended in PBS for flow cytometry analysis. Secretory loads of multiple myeloma cells were measured by calculating the ratio of intracellular immunoglobulin MFI in brefeldin A-treated cells relative to steady-state cells. Briefly, cells were treated with brefeldin A (5 µg/mL) for 4 h, intracellularly stained with an anti–human immunoglobulin G, A, and M (H+L) antibody conjugated to FITC, and analyzed by flow cytometry. Mitochondrial membrane depolarization was measured by JC1 MitoProbe. Briefly, cells washed once by adding warm PBS and incubated in a fresh growth medium with 2 μM of JC-1 dye at 37 °C, 5% CO_2_ for 20 min. Then, cells were washed once by adding warm PBS and analyzed immediately by flow cytometry. For experiments involving viability of AMO-1 cells treated with Z-VAD-FMK, torin-1, and kifunensine at the indicated concentrations overnight, apoptosis was measured by flow cytometry analysis using Annexin V and 7-AAD apoptosis detection kit according to the manufacturer’s protocols. The purity of isolated patient-derived myeloma cells was assessed via flow cytometry following surface staining of CD138, CD19 and CD56 molecules.

**Quantitative analysis of amino acids and biogenic amines**

***Sample preparation***

AMO-1 cells treated with siRNAs for 48 h or cycloheximide (25 ug/ml) for 4 h were collected and washed three times with PBS. Cells were then resuspended at a density of 1.0 x 10^6^ cells per 250 μL of PBS, and subjected to homogenization with repeated freeze thaw cycles following mechanical disruption using 25G syringe. The homogenates were clarified by centrifugation at 20,000x g for 15 min and clear supernatants were collected. Protein concentrations were determined by the Bradford assay. The supernatants were stored at –80°C.

***Chemicals***

Methanol, acetonitrile, and water (all HPLC grade) were obtained from Honeywell (Seelze, Germany). Triethylamine, phenyl isothiocyanate, acetic acid, the amino acid standards (A9906, asparagine, glutamine, kynurenine), and the stable isotope labeled amino acid mix solution (96378) were purchased from Sigma-Aldrich (St. Louis, MO, USA). Tryptophan-D5 were provided by Toronto Research Chemicals (Toronto, Ontario, Canada). To prepare stock solutions of asparagine, glutamine, kynurenine and tryptophan-D5, 1.0 mg of the standards were dissolved in methanol-water (1:1, v/v). For the preparation of calibration standards, the reference standard solutions were mixed and dissolved in methanol-water (1:1, v/v) to reach concentrations between 50 nM and 250 µM.

***Derivatization procedure***

10 µL of the cell lysates or calibration standards were pipetted into a 1.5 mL vial and mixed with 5 µL of the internal standard solution (2.5 µM of each internal standard in methanol-water (1:1, v/v)) as well as 65 µL methanol, 5 µL water, 10 µl triethylamine and 10 µL phenyl isothiocyanate. Derivatization was performed at room temperature for 30 min, after which, the excess reagent was removed by drying at 60 °C with a gentle stream of nitrogen. The dried samples were dissolved with 100 µL of methanol-water (1:3, v/v).

***Liquid chromatography-tandem mass spectrometry***

The LC-MS/MS system consisted of Acquity UPLC H-Class Plus Bio System (Waters, Milford, MA, USA) and a QTrap 6500+ mass spectrometer (Sciex, Framingham, MA, USA). Separations were accomplished on an Eclipse XBD-C18 column (3.5 µm, 3.0 × 100 mm, Agilent) using a ten-minute gradient of 2–98% acetonitrile in aqueous 0.5% acetic acid solution. The flow rate was set to 250 µL/min, and the column temperature was kept at 50 °C. The injection volume was 10 µL. Mass spectrometry detection was performed with electrospray ionization in positive ion mode. Multiple reaction monitoring was carried out using the precursor-to-product ion transitions summarized in Table 1. The peak area ratios of analyte-specific ions to the corresponding internal standard ions obtained from the calibration standards versus concentrations were used to fit 1/x−weighted linear, least squares regression models. The calibration models were used to calculate the analyte concentrations from the peak area ratios of analyte-specific ions to the corresponding internal standard ions obtained from the samples.

**Table 1.** *Multireaction monitoring transitions for the targets and the corresponding internal standards*.

| **Target** | **Q1 (*m/z*)** | **Q3 (*m/z*)** | **Internal Standard** |
| --- | --- | --- | --- |
| Alanine | 225 | 44 | Alanine-^13^C_3_^15^N |
| Arginine | 310 | 217 | Arginine-^13^C_6_ |
| Aspartic acid | 269 | 116 | Aspartic acid-^13^C_4_ |
| Glutamic acid | 283 | 130 | Glutamic acid-^13^C_5_ |
| Glycine | 211 | 76 | Glycine-^13^C_2_^15^N |
| Histidine | 291 | 110 | Histidine-^13^C_6_ |
| Leucine | 267 | 43 | Leucine-^13^C_6_^15^N |
| Isoleucine | 267 | 69 | Isoleucine-^13^C_6_^15^N |
| Lysine | 417 | 324 | Lysine-^13^C_6_ |
| Methionine | 285 | 104 | Methionine-^13^C_5_^15^N |
| Phenylalanine | 301 | 120 | Phenylalanine-^13^C_6_ |
| Proline | 251 | 70 | Proline-^13^C_5_ |
| Serine | 241 | 60 | Serine-^13^C_3_^15^N |
| Threonine | 255 | 74 | Threonine-^13^C_4_ |
| Tryptophan | 340 | 188 | Tryptophan-D_5_ |
| Tyrosine | 317 | 136 | Tyrosine-^13^C_6_ |
| Valine | 253 | 72 | Valine-^13^C_5_ |
| Asparagine | 268 | 87 | Serine-^13^C_3_^15^N |
| Carnosine | 362 | 110 | Arginine-^13^C_6_ |
| Citrulline | 311 | 113 | Serine-^13^C_3_^15^N |
| Glutamine | 282 | 130 | Serine-^13^C_3_^15^N |
| Kynurenine | 344 | 146 | Tryptophan-D_5_ |
| Ornithine | 403 | 310 | Methionine-^13^C_5_^15^N |
| c4-OH-Pro | 267 | 68 | Arginine-^13^C_6_ |
| Alanine-^13^C_3_^15^N | 229 | 47 |  |
| Arginine-^13^C_6_ | 316 | 223 |  |
| Aspartic acid-^13^C_4_ | 273 | 120 |  |
| Glycine-^13^C_2_^15^N | 214 | 79 |  |
| Histidine-^13^C_6_ | 297 | 115 |  |
| Leucine-^13^C_6_^15^N | 274 | 92 |  |
| Isoleucine-^13^C_6_^15^N | 423 | 330 |  |
| Lysine-^13^C_6_ | 307 | 126 |  |
| Phenylalanine-^13^C_6_ | 256 | 74 |  |
| Proline-^13^C_5_ | 245 | 63 |  |
| Serine-^13^C_3_^15^N | 259 | 63 |  |
| Threonine-^13^C_4_ | 259 | 77 |  |
| Tyrosine-^13^C_6_ | 323 | 142 |  |
| Valine-^13^C_5_ | 258 | 76 |  |
| Glutamic acid-^13^C_5_ | 288 | 135 |  |
| Methionine-^13^C_5_^15^N | 291 | 109 |  |
| Tryptophan-D_5_ | 345 | 193 |  |

The MxP® Quant 500 kit (Biocrates, Innsbruck, Austria) was used as a ready-to-use platform according to the manufacturer’s instructions for targeted metabolite quantification in Supplementary Figures 14 and 15. Data were processed using the Biocrates proprietary MetIDQ software, which is an integral component of the kit.

**ELISA**

Supernatants for immunoglobulin analysis were harvested after 48 hours from wells in which AMO-1 or NCI-H929 MM cell lines were transfected with siRNA targeting a non-specific sequence (Ctrl) or siRNA targeting SEC24A&B. Supernatants were centrifuged at 500x g for 5 min to exclude any cell contamination. Then, cell-free supernatants were harvested and incubated with IgA antibody-coated ELISA plates according to the manufacturer’s instructions. All samples were assayed in duplicate. IgA concentrations were calculated by interpolation from a standard curve generated using a microplate reader at a wavelength of 450 nm. To quantify GFP levels of tumor spheroids containing GFP-CaaX transfected MM cells, each MM spheroid transferred into 0.5 mL cell extraction buffer PTR containing 200 µg/ml protease inhibitors. After 10 min of incubation on ice, three freeze-thaw cycles were performed using liquid nitrogen and 37ºC water bath*,* with vigorous vortexing after each cycle. The homogenates were then centrifuged at 18,000 x g for 20 minutes at 4°C. The supernatants were collected and further diluted 10-fold. Following preparation of samples, GFP levels were determined using SimpleStep ELISA kit according to the manufacturer’s instructions.

**Image analysis**

All image processing steps are carried out using the ImageJ software (version 1.53q, National Institutes of Health). In the first step, all fluorescence images were background subtracted. ERES images were filtered using LOG3D plugin (Sage et al. 2005) before thresholding. Identified ERES were analyzed with ‘Analyze Particles’ command. Image stacks of ER were filtered, segmented and analyzed by plugins of 3D ImageJ Suite (Ollion et al. 2013).

**Chick embryo chorioallantoic membrane (CAM) assay**

CAM of fertilized White Leghorn chicken eggs was used mainly following the protocol published by Martowicz (Martowicz et al. 2015). MM cells expressing GFP-CaaX treated with siRNAs for 24 hr were collected and suspended at a density of 250,000 cells per 30 μL of growth factor-reduced Matrigel. MM spheroids were formed with the polymerization of Matrigel for 30 min at 37°C. MM spheroids were then placed on the surface of the CAM (2 cm away from the embryo), in 9-day-old chicken embryos (four onplants for per embryo). After four days of in vivo growth in an egg incubator (Grumbach) at 37°C and 70% humidity, MM spheroids were imaged using a fluorescence stereomicroscope (Olympus SZX10), and images were acquired with cellSens Standard Imaging Software (v.1.18, Olympus). To quantify the growth of MM spheroids, GFP-levels were measured by a commercial SingleStep GFP ELISA Kit using biotinylated anti-GFP antibodies, according to the manufacturer’s protocol.

**Intrafemural multiple myeloma model in NSG mice**

NOD.Cg-*Prkdc^scid^ Il2rg^tm1Wjl^*/SzJ (NSG) ) immunocompromised mice were purchased from Kobay Experimental Animal Production Laboratory (Türkiye) and maintained under specific pathogen-free conditions in individually ventilated cages with ad libitum access to food and water. All animal procedures were approved by Animal Care and Use Committee of Kobay Laboratory [Ethical approval no: **778**] and were performed in accordance with national and institutional ethical guidelines. For generation of the orthotopic MM model, inducible shRNA-expressing NCI-H929 cells (50.000 cells in 10 μL) were injected intrafemorally into the NSG mice (6–8 weeks old). Tumors were allowed to establish for 2 weeks before induction of shRNA expression. Doxycycline was administered intraperitoneally (i.p.) at a dose of 2.5 mg/kg, twice at 5-day intervals. Following two rounds of doxycycline induction, the mice were injected i.p. with 100 μl of CycLuc1 (5 mM) and imaged ten minutes post-injection. Tumor progression was monitored by bioluminescent imaging (BLI) using an in vivo imaging system (Newton 7.0). The total photon flux (photons/sec) was measured from region over the entire hindlimb of the mouse. All tumor inoculation and imaging procedures were conducted under anesthesia with ketamine (60 mg/kg) and xylazine (10 mg/kg).

**Ethics statement and clinical specimens**

Bone marrow aspirates obtained from patients with newly diagnosed or relapsed MM following signed informed consent in compliance with the Declaration of Helsinki. The study was approved by the ethics committee of the Medical University of Innsbruck (Nr: 1198/2021). Bone marrow samples were subjected to red blood cell (RBC) lysis, according to manufacturer’s instructions (Biolegend). Cells were washed twice with PBS and resuspended in MACS buffer (Miltenyi Biotech). Then, CD138^+^ myeloma cells were separated by positive MACS selection using CD138^+^ microbeads. After purity check by surface staining of CD138, CD19 and CD56 molecules (as described above), CD138^+^ myeloma cells at a purity over 75% were used in assays. For survival analyses, we used the survival data from the Multiple Myeloma Research Foundation (MMRF) CoMMpass Study dataset. Transcript per Million (TPM) reads values of SEC24 paralogs were used for survival analyses. The MMRF genomic data can be found on the Genomic Data Commons (GDC) Data Portal (<https://portal.gdc.cancer.gov/>).

SUPPLEMENTARY FIGURES:


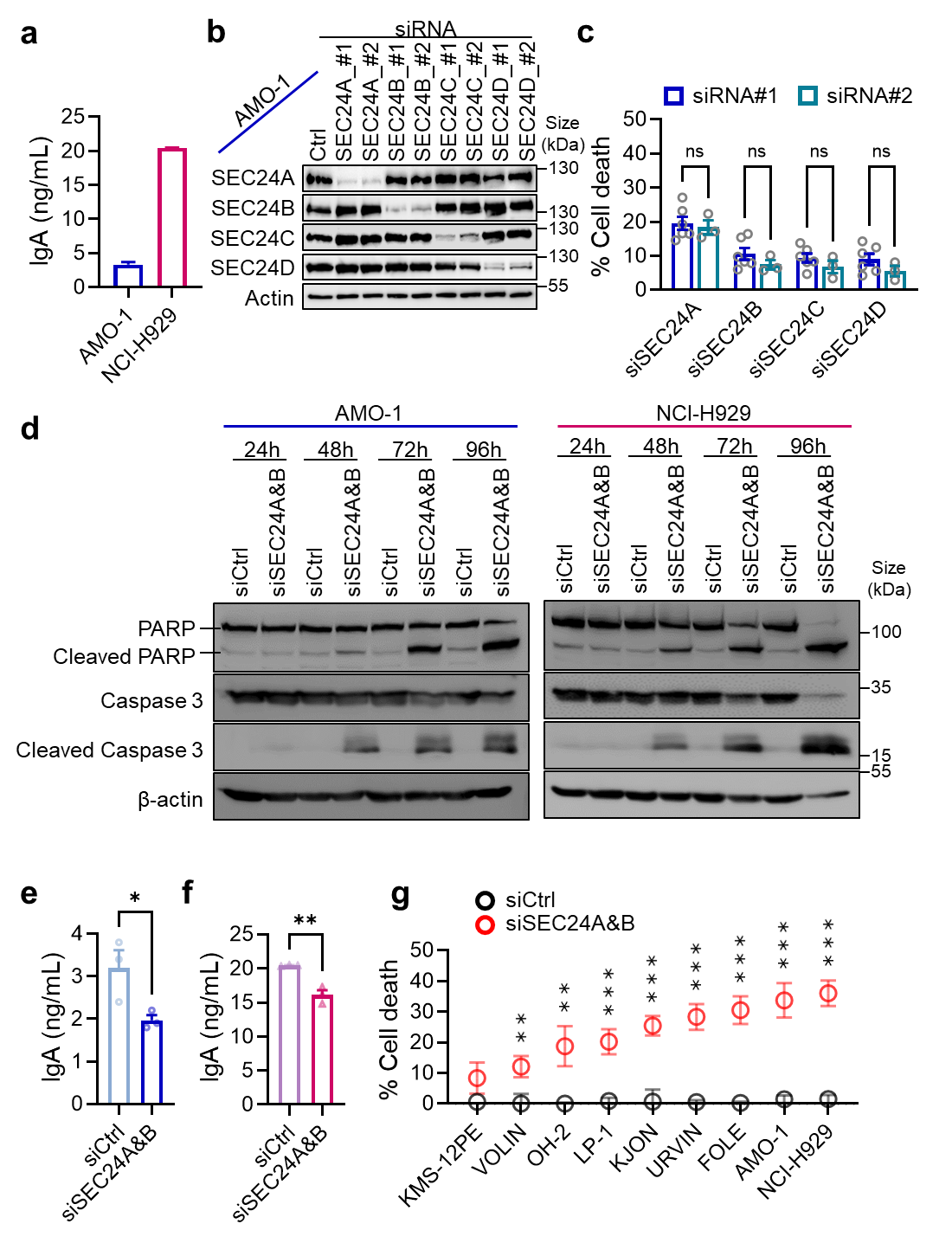


Figure S1. a ELISA quantification of IgA titers in AMO-1 and NCI-H929 MM cell lines. The supernatant was collected at 48 hours for IgA ELISA analysis. b Rationally designed siRNA oligos targeting SEC24 paralogs were evaluated for silencing efficiency. Transient transfections of AMO-1 cells with siRNAs against SEC24 paralogs demonstrated acceptable knockdown efficiencies, as determined by immunoblotting after 48 hours. c After 48 hours of knockdown, cell viability was measured for each siRNA targeting SEC24 paralogs in AMO-1 cells. Cell viability was assessed using the CellTiter-Glo assay, and data were normalized to cells treated with negative control siRNA. ns = non-significant, Student’s unpaired two-tailed t-test. siRNA#1 and siRNA#2 indicate two different siRNAs targeting the same gene. d PARP and Caspase-3 cleavage were detected at multiple time points following co-depletion of SEC24A&B, compared with control condition. (e and f) Changes in IgA secretion were assessed by ELISA using the supernatant of e AMO-1 and f NCI-H929 cells at 48 hours post-transfection with negative control or SEC24A&B siRNAs. g The impact of SEC24A&B depletion on cell viability was assessed in multiple MM cell lines: non-secretory KMS-12PE, IgG-secreting VOLIN, IgG-secreting OH-2, IgG-secreting LP-1, FLC-secreting KJON, IgG-secreting URVIN, IgA/FLC-secreting FOLE, IgA-secreting AMO-1, and IgA-secreting NCI-H929. Cell viability was assessed using the CellTiter-Glo assay. Gray circles, siCtrl; red circles, siSEC24A&B. ***P < .001, **P < .01, Student’s unpaired two-tailed t-test.


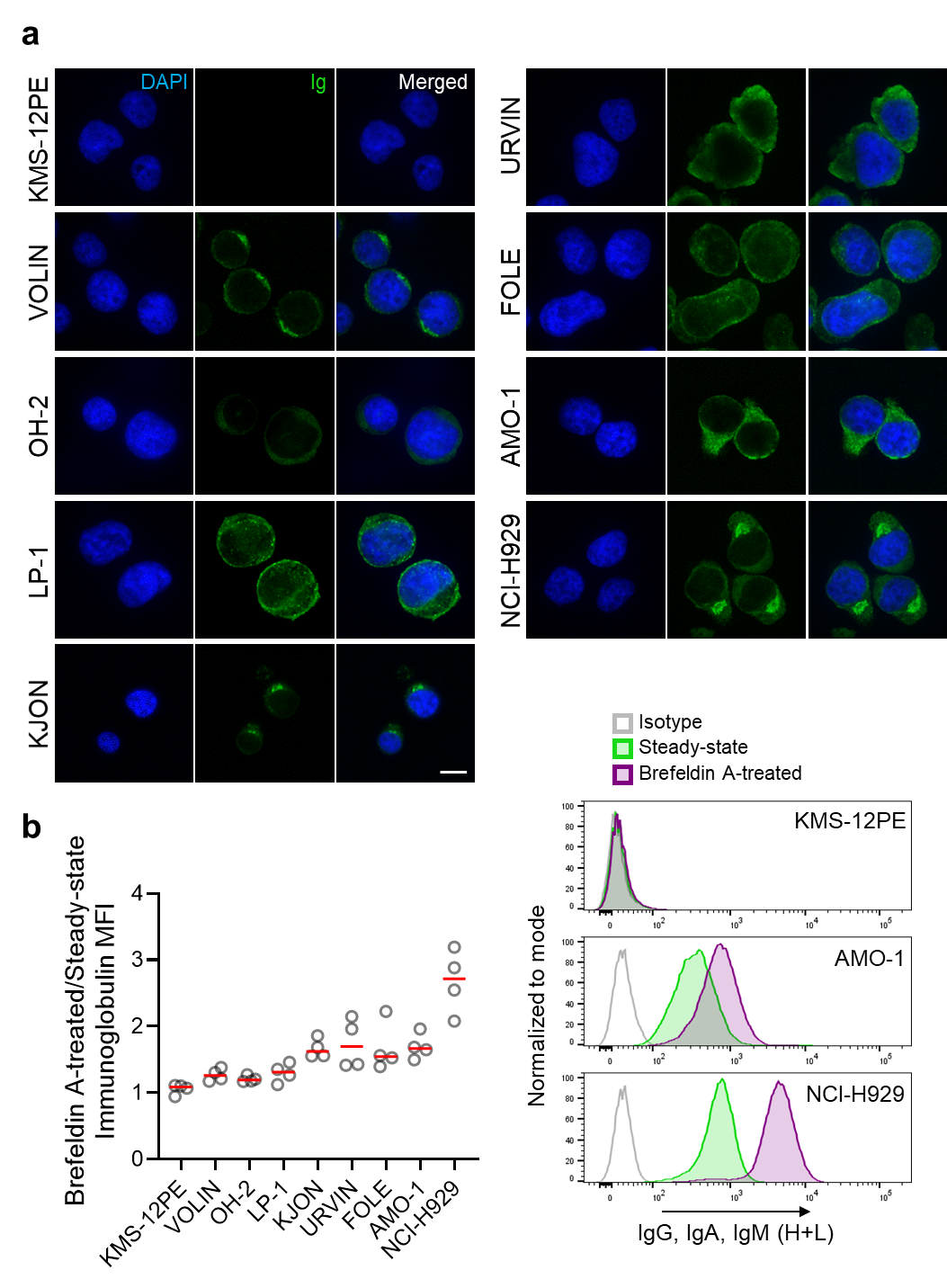


Figure S2. a Multiple myeloma cells were labeled at steady state with an anti-human immunoglobulin G, A, and M (H+L) antibody conjugated to FITC and counterstained with DAPI to visualize nuclei. All images were acquired using identical microscopy settings. Scale bar, 10 µm. b, Multiple myeloma cells were treated with brefeldin A (5 µg/mL) for 4 h and intracellularly stained with an anti-human immunoglobulin G, A, and M (H+L) antibody conjugated to FITC. Immunoglobulin median fluorescence intensity (MFI) in brefeldin A–treated cells was normalized to steady-state MFI (n = 4 independent experiments). Overlaid histograms show representative flow cytometry profiles of the non-secretory KMS-12PE, moderately secretory AMO-1, and hypersecretory NCI-H929 cell lines at their steady state and Brefeldin A-treated states.


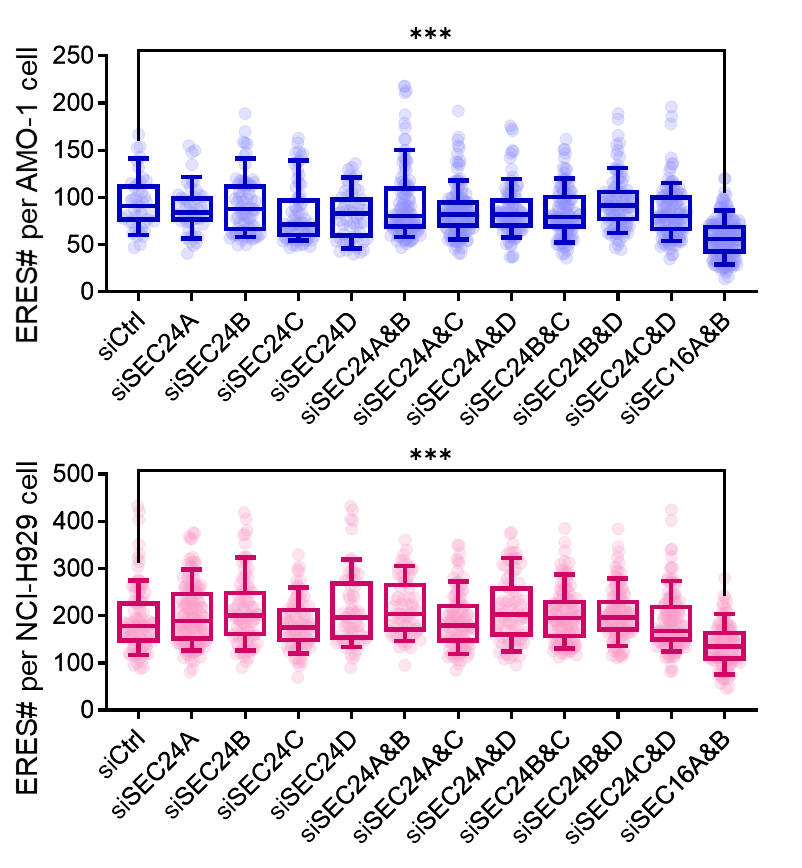


**Figure S3.** Knockdown of any of the SEC24 paralogs did not exert a negative effect on ERES. AMO-1 (blue) and NCI-H929 (pink) cells were treated with siRNA against the indicated gene, followed by fixation and staining after 48 hours with anti-SEC31 to label ERESs. Quantification of the number of ERESs per cell is displayed. Data are from three independent experiments with at least 60 cells per condition. Each symbol represents individual data points. Lines represent the median, edges represent the interquartile range, and whiskers represent the 10th and 90th percentiles. Statistical significance was measured using one-way ANOVA followed by Dunnett’s test, ***P < .001. Statistical significance is presented only in comparison to the control.


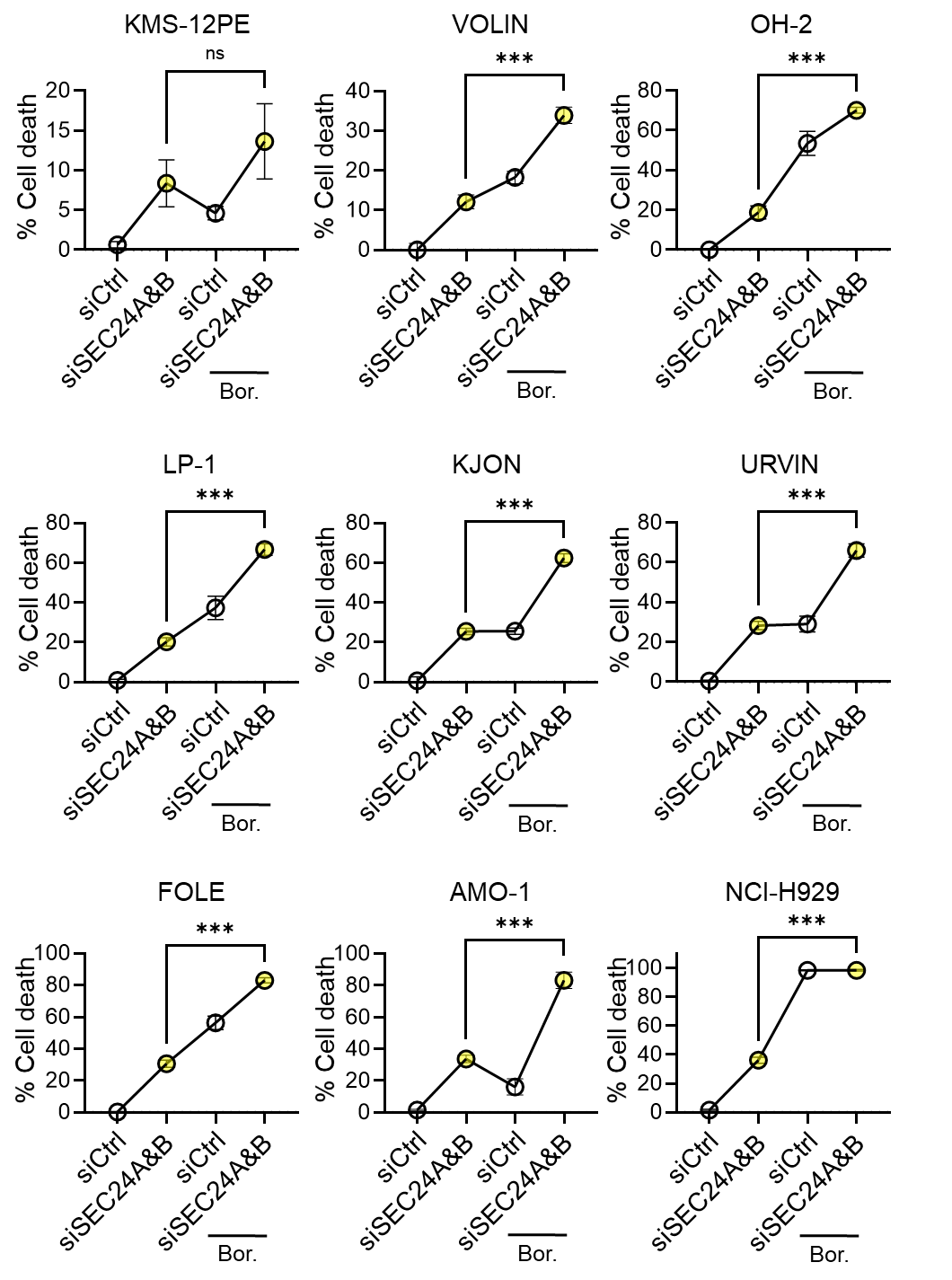


**Figure S4.** The effect of bortezomib (Bor.) after SEC24A&B depletion on cell viability was assessed in multiple MM cell lines: non-secretory KMS-12PE, IgG-secreting VOLIN, IgG-secreting OH-2, IgG-secreting LP-1, FLC-secreting KJON, IgG-secreting URVIN, IgA/FLC-secreting FOLE, IgA-secreting AMO-1, and IgA-secreting NCI-H929. Cells were treated with 5 nM bortezomib during the last 16 hours of the 48-hour knockdown. Gray circles, siCtrl; yellow-filled circles, siSEC24A&B. Data were generated in ≥3 independent experiments. ***P < .001, Student’s unpaired two-tailed t-test.


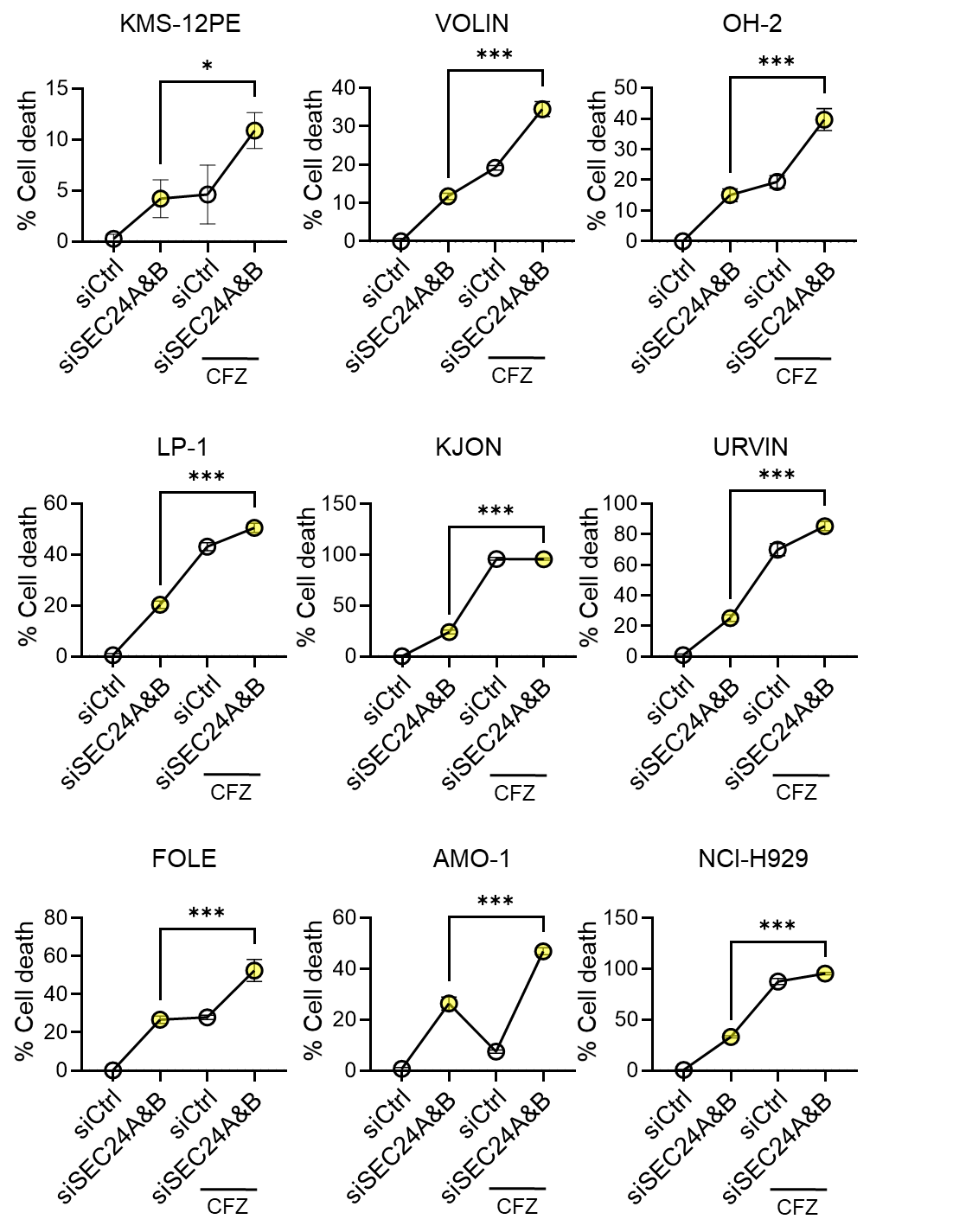


**Figure. S5.** The effect of carfilzomib (CFZ) after SEC24A&B depletion on cell viability was assessed in multiple MM cell lines: non-secretory KMS-12PE, IgG-secreting VOLIN, IgG-secreting OH-2, IgG-secreting LP-1, FLC-secreting KJON, IgG-secreting URVIN, IgA/FLC-secreting FOLE, IgA-secreting AMO-1, and IgA-secreting NCI-H929. Cells were treated with 10 nM carfilzomib during the last 16 hours of the 48-hour knockdown. Gray circles, siCtrl; yellow-filled circles, siSEC24A&B. Data were generated in ≥3 independent experiments. ***P ≤ 0.001, * P ≤ 0.05, Student’s unpaired two-tailed t-test.


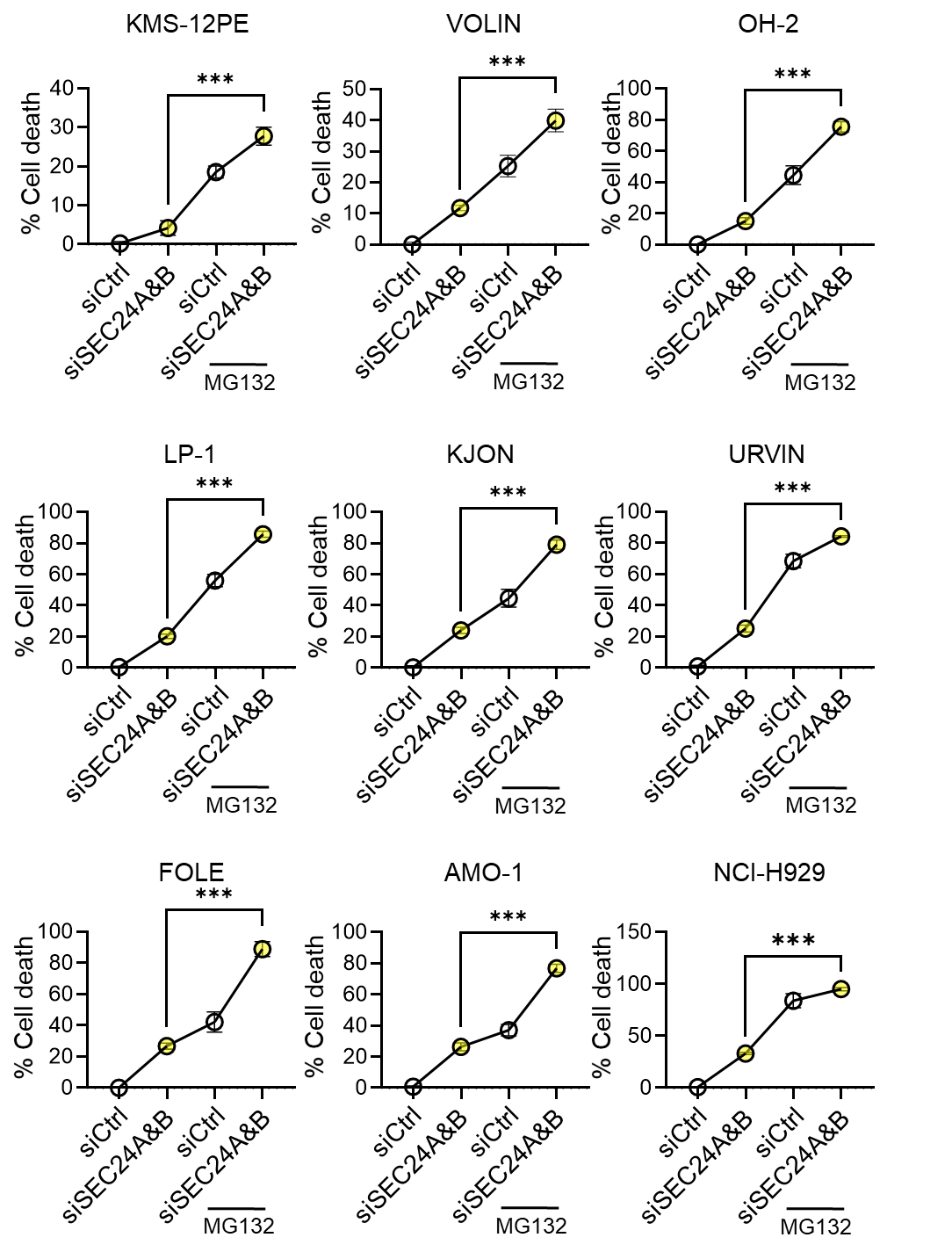


**Figure S6.** The effect of MG132 after SEC24A&B depletion on cell viability was assessed in multiple MM cell lines: non-secretory KMS-12PE, IgG-secreting VOLIN, IgG-secreting OH-2, IgG-secreting LP-1, FLC-secreting KJON, IgG-secreting URVIN, IgA/FLC-secreting FOLE, IgA-secreting AMO-1, and IgA-secreting NCI-H929. Cells were treated with 10 μM MG132 during the last 16 hours of the 48-hour knockdown. Gray circles, siCtrl; yellow-filled circles, siSEC24A&B. Data were generated in ≥3
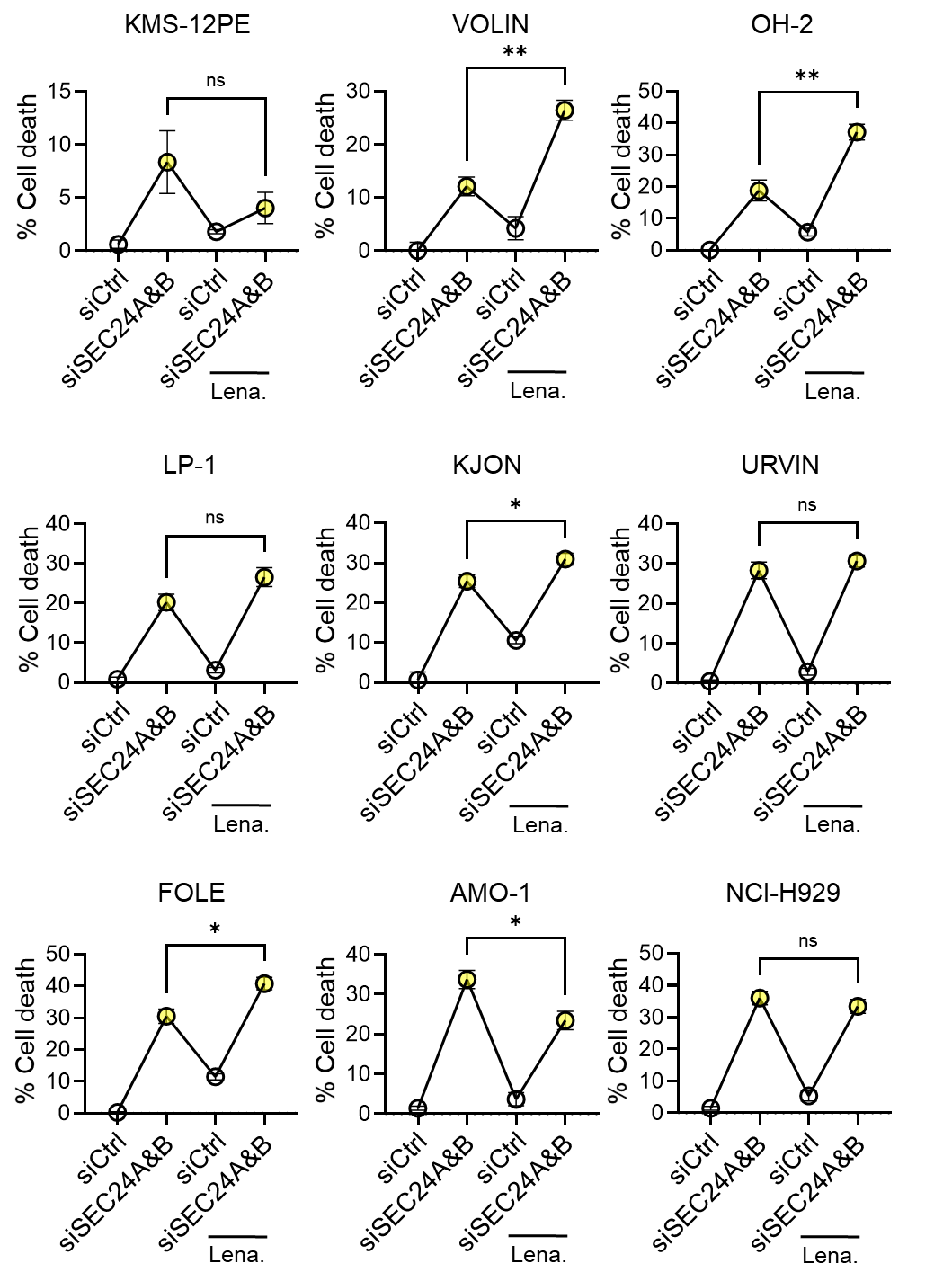
independent experiments. ***P < .001, Student’s unpaired two-tailed t-test.

**Figure S7.** The effect of lenalidomide after SEC24A&B depletion on cell viability was assessed in multiple MM cell lines: non-secretory KMS-12PE, IgG-secreting VOLIN, IgG-secreting OH-2, IgG-secreting LP-1, FLC-secreting KJON, IgG-secreting URVIN, IgA/FLC-secreting FOLE, IgA-secreting AMO-1, and IgA-secreting NCI-H929. Cells were treated with 50 μM lenalidomide during the knockdown period. Cell viability were measured using the CellTiter-Glo assay after 48 hours of knockdowns. Gray circles, siCtrl; yellow-filled circles, siSEC24A&B. Data were generated in ≥3 independent experiments. ***P < .001, **P < .01, *P < .05, Student’s unpaired two-tailed t-test.


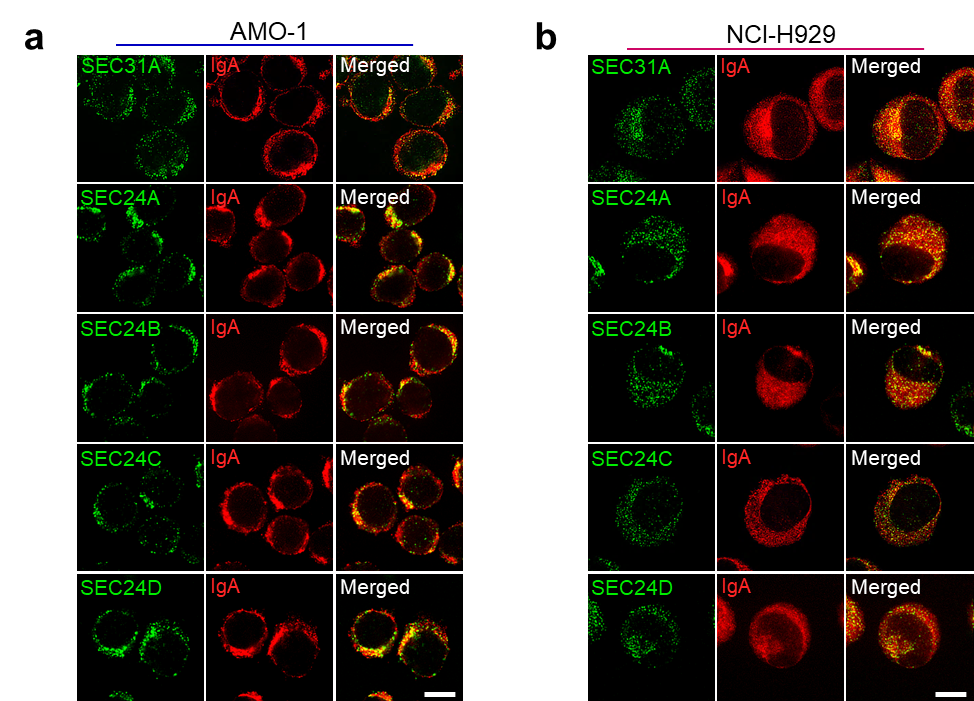


Figure S8. a AMO-1 or b NCI-H929 cells were incubated at 10°C for 60 min to increase the local concentration of Igs at ERES. Cells were then spun onto coverslips and processed for immunofluorescence staining using antibodies against SEC24 paralogs (green) and IgA (red). SEC31A staining (green) was used as a positive control representing almost all ERES. Scale bar, 10 µm.


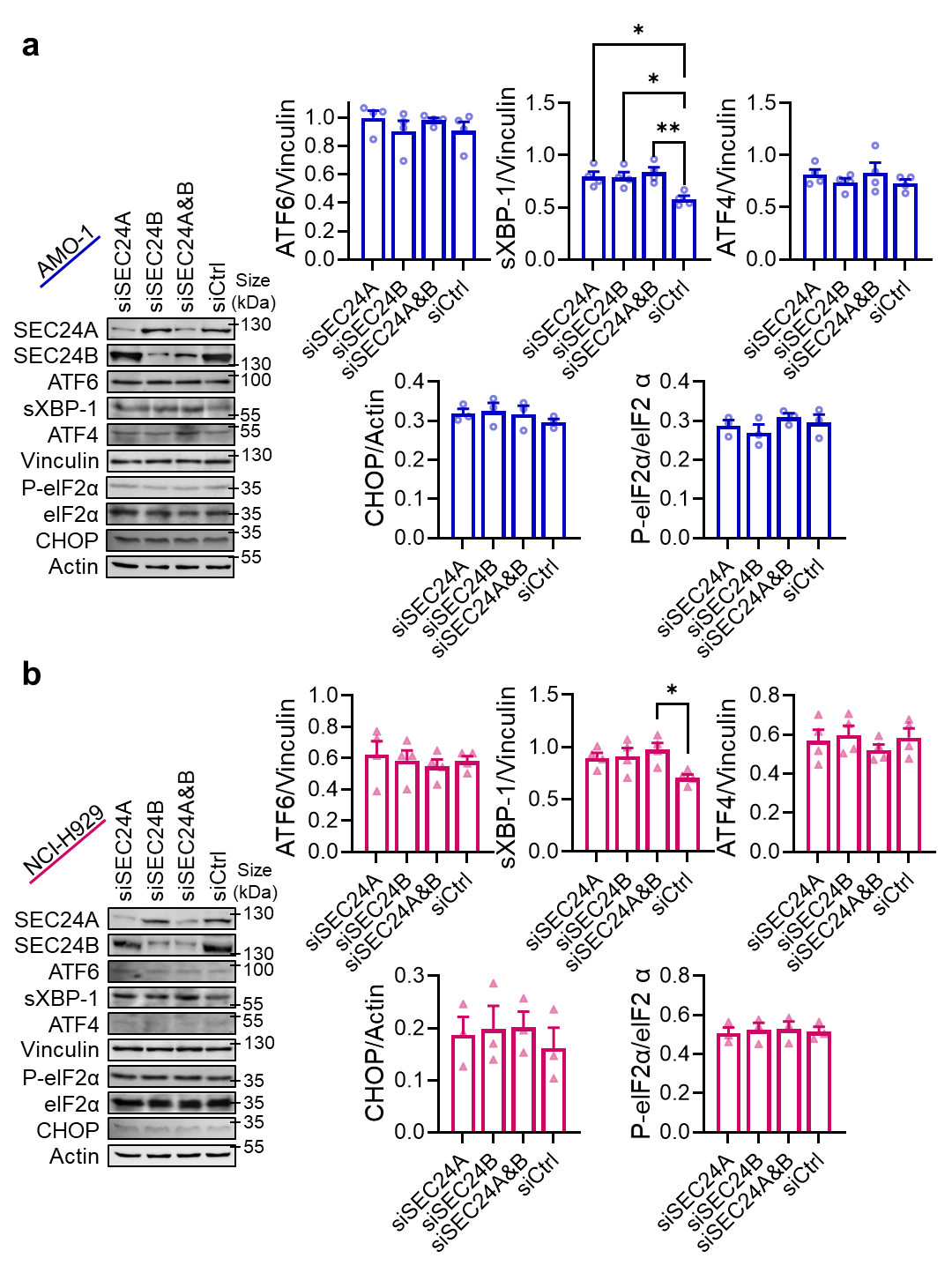


**Figure S9.** Immunoblot quantifications of ATF6, sXBP-1, ATF4, phosphor-eIF2α, and CHOP in **a** AMO-1 and **b** NCI-H929 cells after 48 hours of knockdown of the indicated SEC24 paralogs and their combinations. Protein band intensity was evaluated using ImageJ software and normalized to vinculin or actin. Significance was tested using the Tukey multiple comparison test (one-way ANOVA, **P < .01; *P < .05). Data are presented as mean values ± SEM.


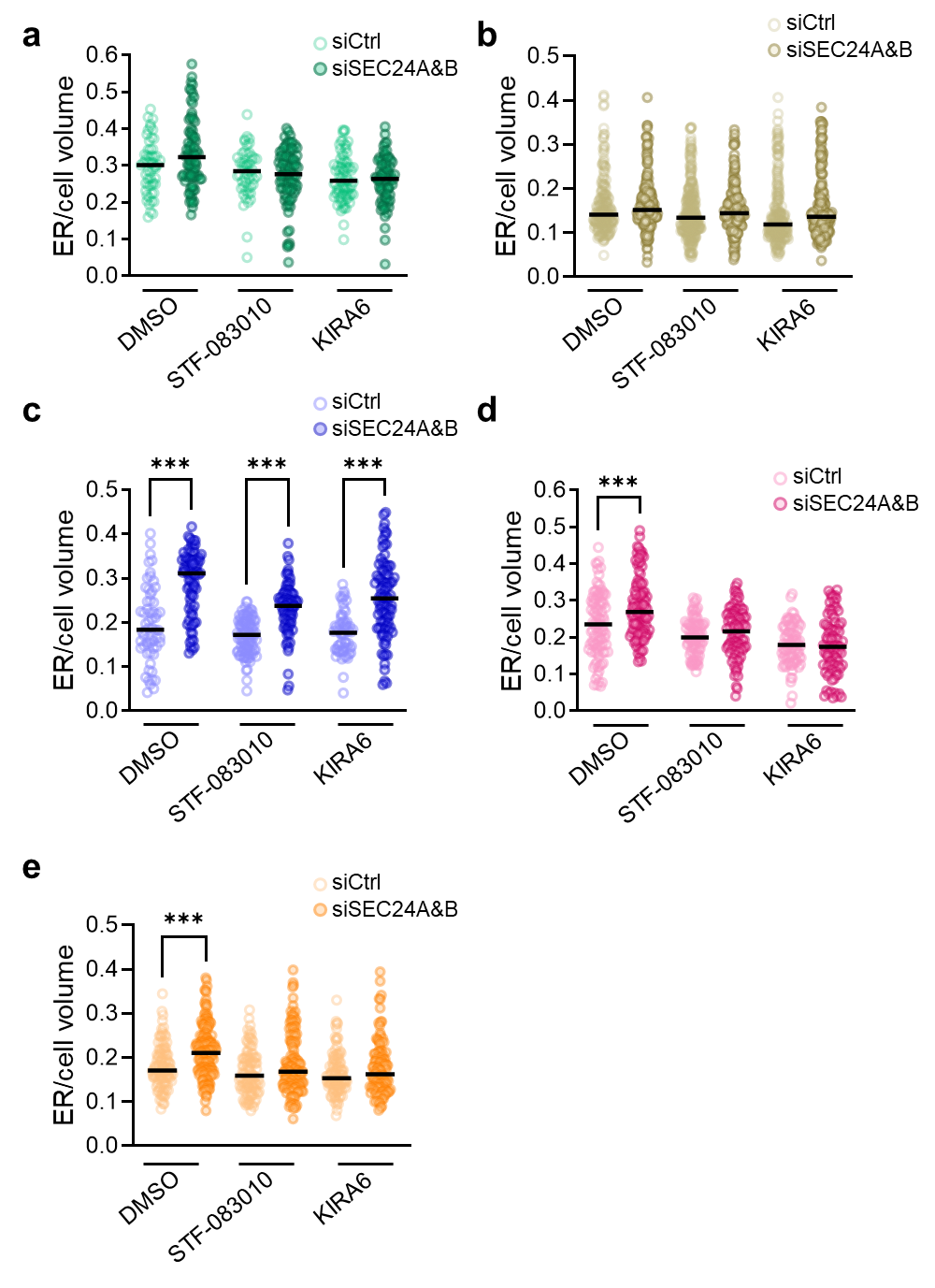


**Figure S10.** The increase in ER volume was dependent on IRE1 activity in the secretory MM cell lines. **a** KMS-12PE, **b** OH-2, **c** AMO-1, **d** NCI-H929, and **e** FOLE cell lines were treated with control or SEC24A&B siRNAs for 48 hours in the presence of DMSO (Ctrl), 10 µM STF-083010, or 2 nM KIRA-6 for 16 hours. Cells were stained with calnexin for the ER, and confocal images of 3D reconstructions of the ER were acquired. Image stacks with 0.1 μm steps along the z-axis were used to analyze ER morphology using ImageJ 3D-suite. ER volume was normalized to the corresponding cell volume. Quantification was performed from three independent experiments. Horizontal solid black lines represent medians. Dots represent individual cells (n > 60 for each condition). Statistical analysis was performed using one-way ANOVA with Tukey’s correction for multiple comparisons, ***P < .001. Statistical significance is illustrated only when comparing with the corresponding control condition.


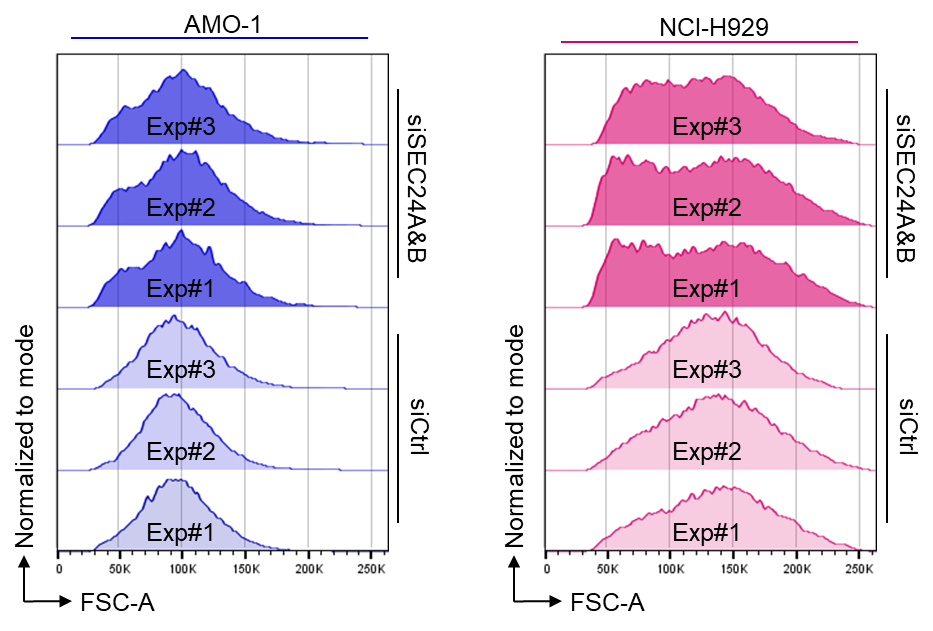


Figure S11. AMO-1 or NCI-H929 cells were transfected with either a non-targeting control siRNA (siCtrl) or siRNA targeting SEC24A&B for 48 hours. Cell size was measured by flow cytometry. Overlaid histograms represent three independent experiments (exp #1, 2, and 3).


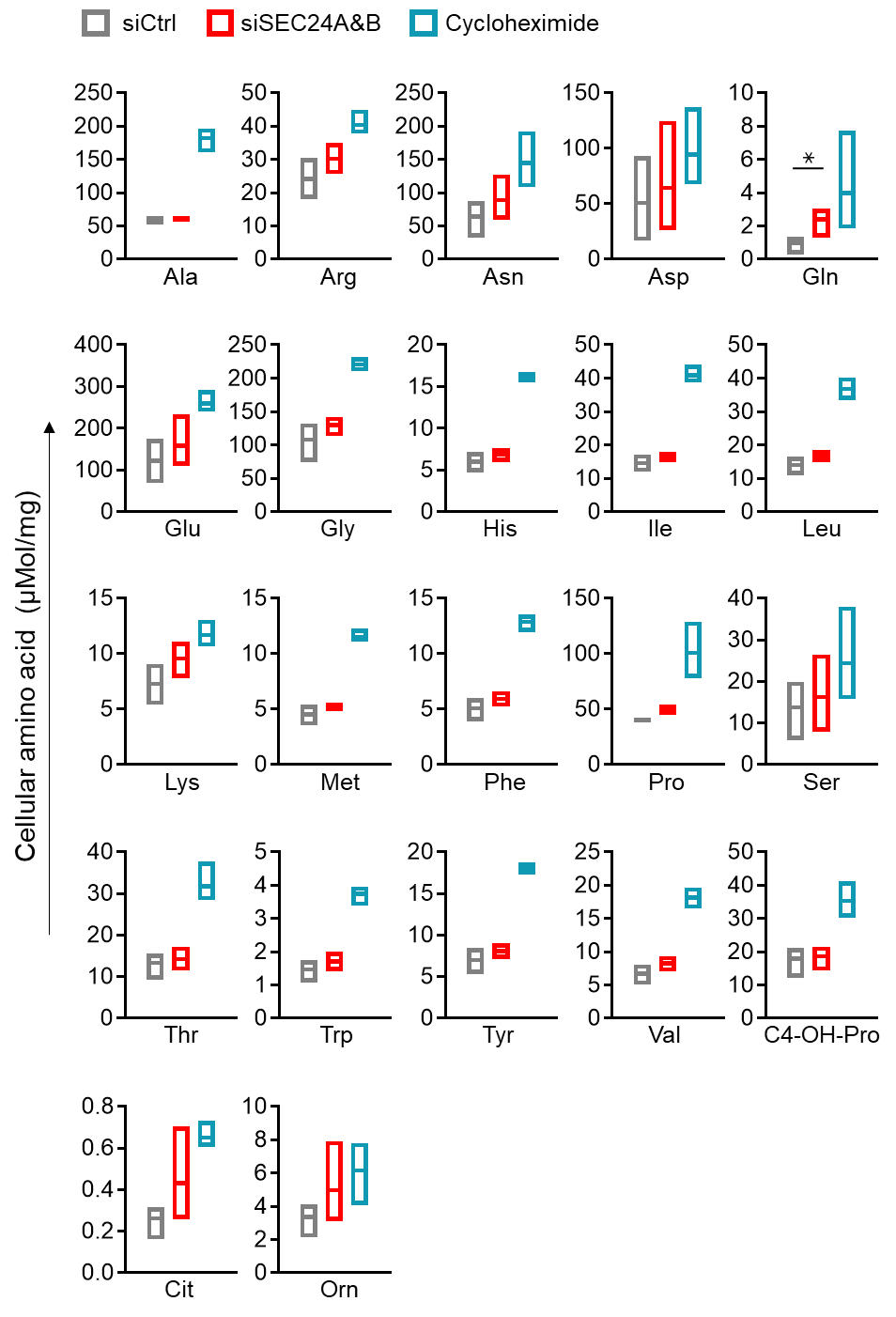


**Figure S12.**

Alteration of ER export resulted in an increase in almost all amino acids. AMO-1 cells were transfected with siRNA targeting a non-specific sequence (Ctrl) or siRNA targeting SEC24A&B for 48 hours, or treated with 25 μg/ml cycloheximide for 6 hours. Cells were then homogenized by three freeze-thaw cycles and passed through a 25G syringe at least 30 times, followed by centrifugation at 20,000 g for 20 minutes. Intracellular amino acid levels were measured in the supernatants. The data were normalized to protein concentration using the Bradford method. Floating bars represent the minimum to maximum, with a line at the median (n = 3 independent experiments). Statistical significance was measured using one-way ANOVA followed by Dunnett’s test, *P < .05. Statistical significance is illustrated only when comparing the siSEC24A&B-treated condition with the control condition.

**
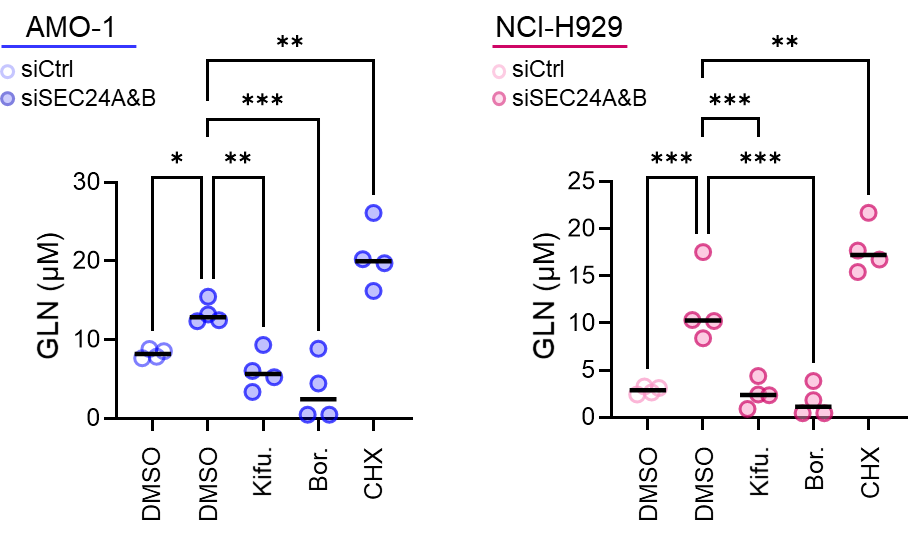
**

**Figure S13.** AMO-1 and NCI-H929 cells were transfected with siRNAs targeting the SEC24A and SEC24B paralogs. Cells were treated with kifunensine (2 µM) for 16 h, bortezomib (500 pM) for 4 h, or cycloheximide (200 nM) for 4 h. Glutamine levels were measured from 20,000 cells using the Glutamine/Glutamate-Glo assay at the end of 48 hours of knockdown. Each circle represents an independent experiment, and horizontal lines indicate median values. Statistical significance was determined by one-way ANOVA with Tukey’s multiple-comparison test (*P < 0.05, **P < 0.01, ***P < 0.001).


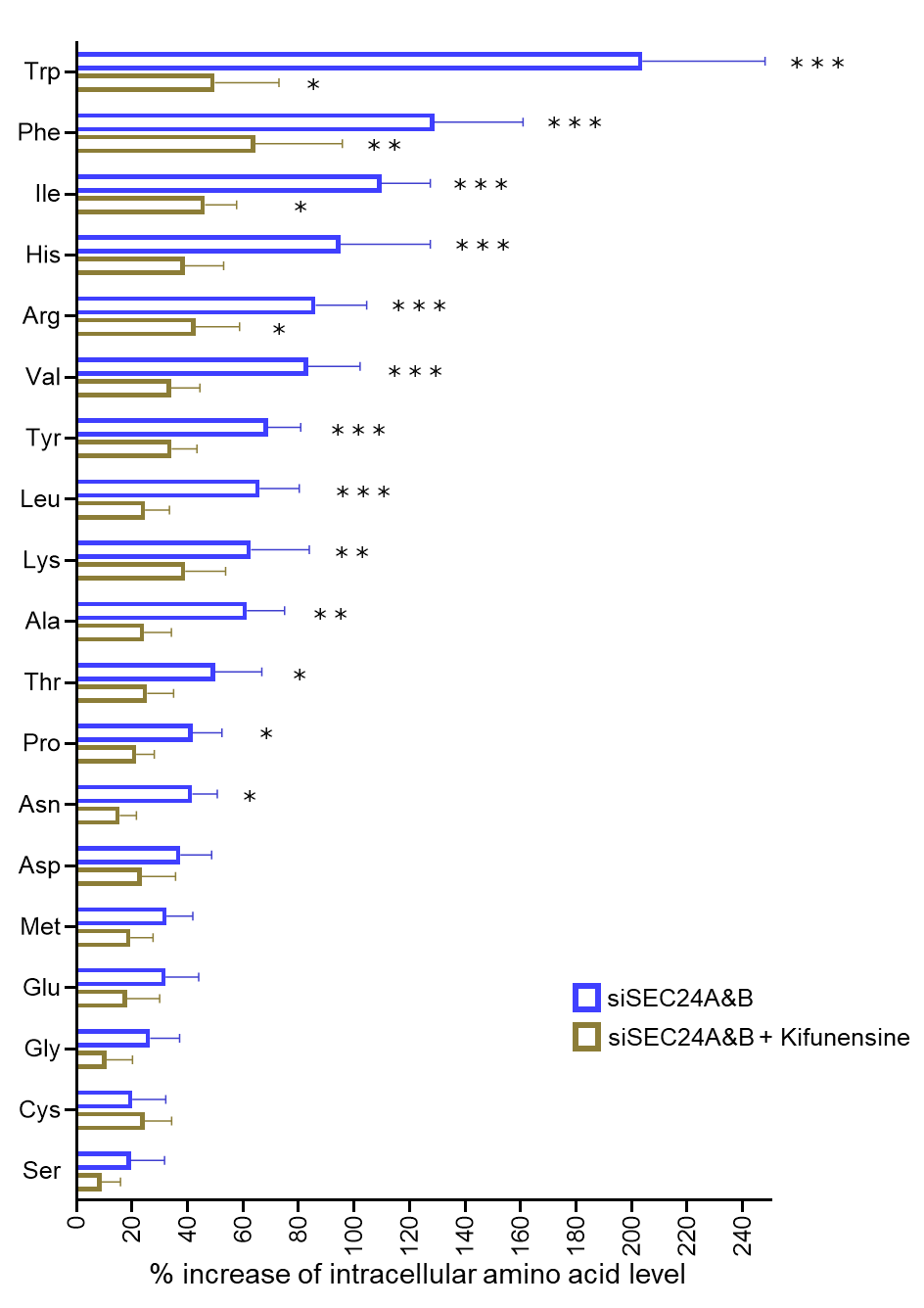


**Figure S14.** AMO-1 cells were transfected with siRNA targeting a non-specific sequence (Ctrl) or siRNA targeting SEC24A&B for 48 hours. SEC24A&B-depleted cells were treated with kifunensine (2 μM) during the last 16 hours of the 48-hour. Cells were then homogenized by three freeze-thaw cycles and passed through a 25G syringe at least 30 times, followed by centrifugation at 20,000 g for 20 minutes. Intracellular amino acid levels were measured in the supernatants. The data were normalized to protein concentration using the Bradford method. Bar graphs represent mean ± SEM from four independent experiments. Statistical significance was assessed using Tukey’s multiple-comparison test following two-way ANOVA (***P < 0.001; **P < 0.01; *P < 0.05). Statistical significance is shown only for comparisons between siSEC24A&B-treated conditions and the siCtrl condition.


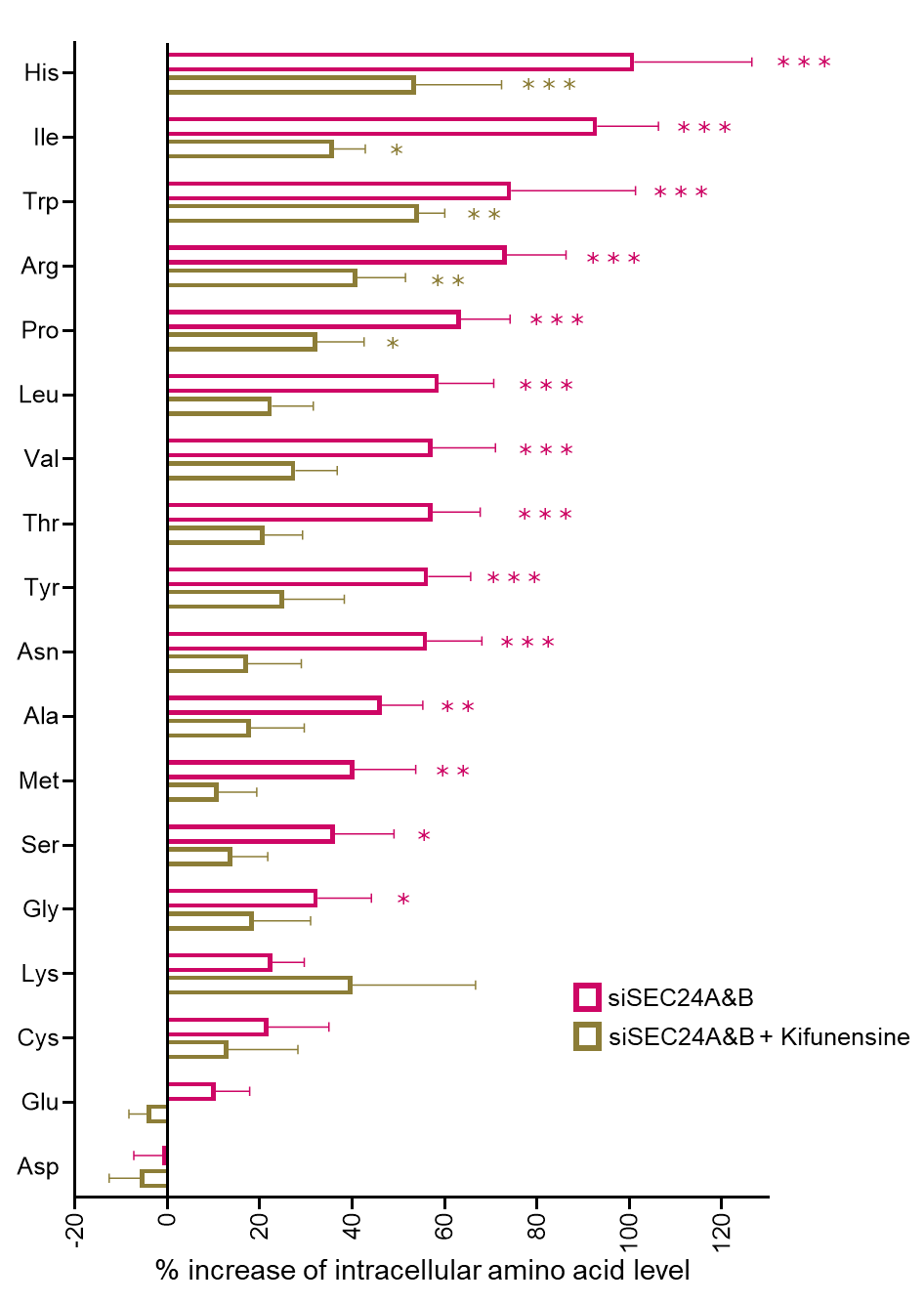


**Figure S15.** NCI-H929 cells were transfected with siRNA targeting a non-specific sequence (Ctrl) or siRNA targeting SEC24A&B for 48 hours. SEC24A&B-depleted cells were treated with kifunensine (2 μM) during the last 16 hours of the 48-hour. Cells were then homogenized by three freeze-thaw cycles and passed through a 25G syringe at least 30 times, followed by centrifugation at 20,000 g for 20 minutes. Intracellular amino acid levels were measured in the supernatants. The data were normalized to protein concentration using the Bradford method. Bar graphs represent mean ± SEM from four independent experiments. Statistical significance was assessed using Tukey’s multiple-comparison test following two-way ANOVA (***P < 0.001; **P < 0.01; *P < 0.05). Statistical significance is shown only for comparisons between siSEC24A&B-treated conditions and the siCtrl condition.


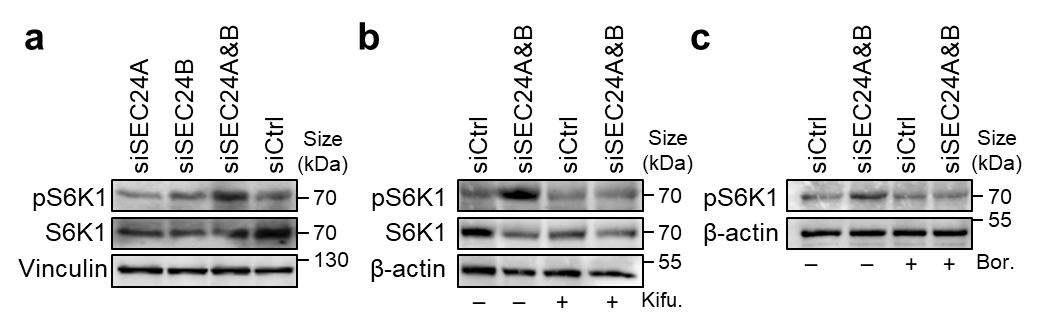


**Figure S16.** Silencing SEC24A&B resulted in high mTORC1 activity. **a** NCI-H929 cells were transfected with the indicated SEC24 siRNAs or control siRNA and subjected to western blot analyses of phosphorylated and total S6K1 after 48 hours of knockdown. **b** NCI-H929 cells transfected with siCtrl or siSEC24A&B were treated with 2 μM of kifunensine for 16 hours, **c** or with 200 nM of bortezomib for 4 hours, and then subjected to immunoblots of phosphorylated and total S6K1 at the end of the 48-hour knockdown period. Data are representative of three or more independent experiments.


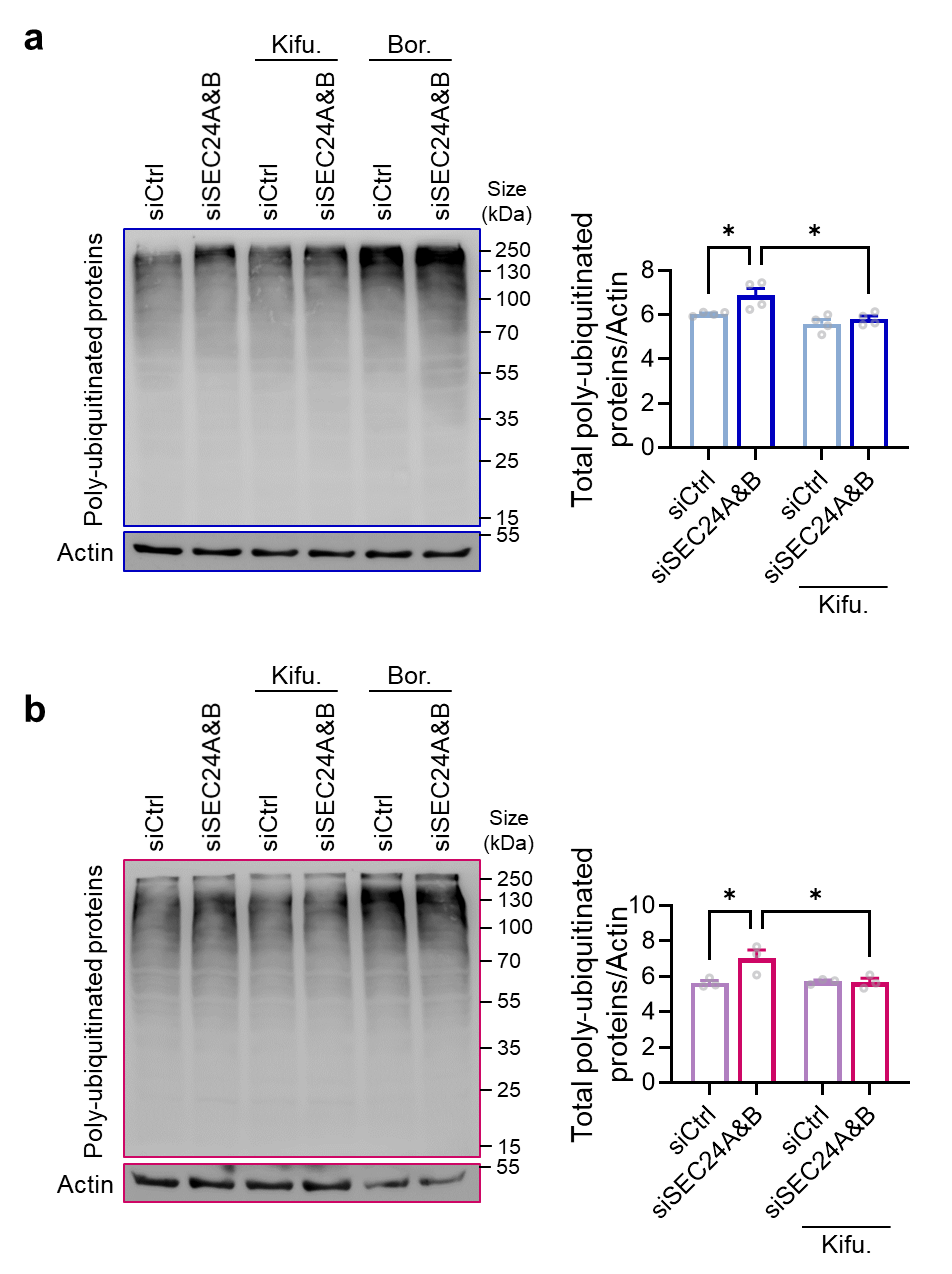


**Figure S17.** Western blot analysis of total ubiquitin-bound protein levels. Western blot performed on a AMO-1 and b NCI-H929 cell lines transfected with siCtrl or siSEC24A&B. Cells were treated with 1 μM of kifunensine for the last 16 hours, or with 200 nM of bortezomib for the last 4 hours, and then subjected to immunoblots of total ubiquitin-bound protein at the end of the 48-hour knockdown period. Total ubiquitin-bound protein levels was quantified by densitometry analysis, normalized on actin levels. Data represent three independent experiments. Statistical significance was determined by one-way ANOVA with Tukey’s multiple comparison test (*P < .05). Data are presented as mean values ± SEM.


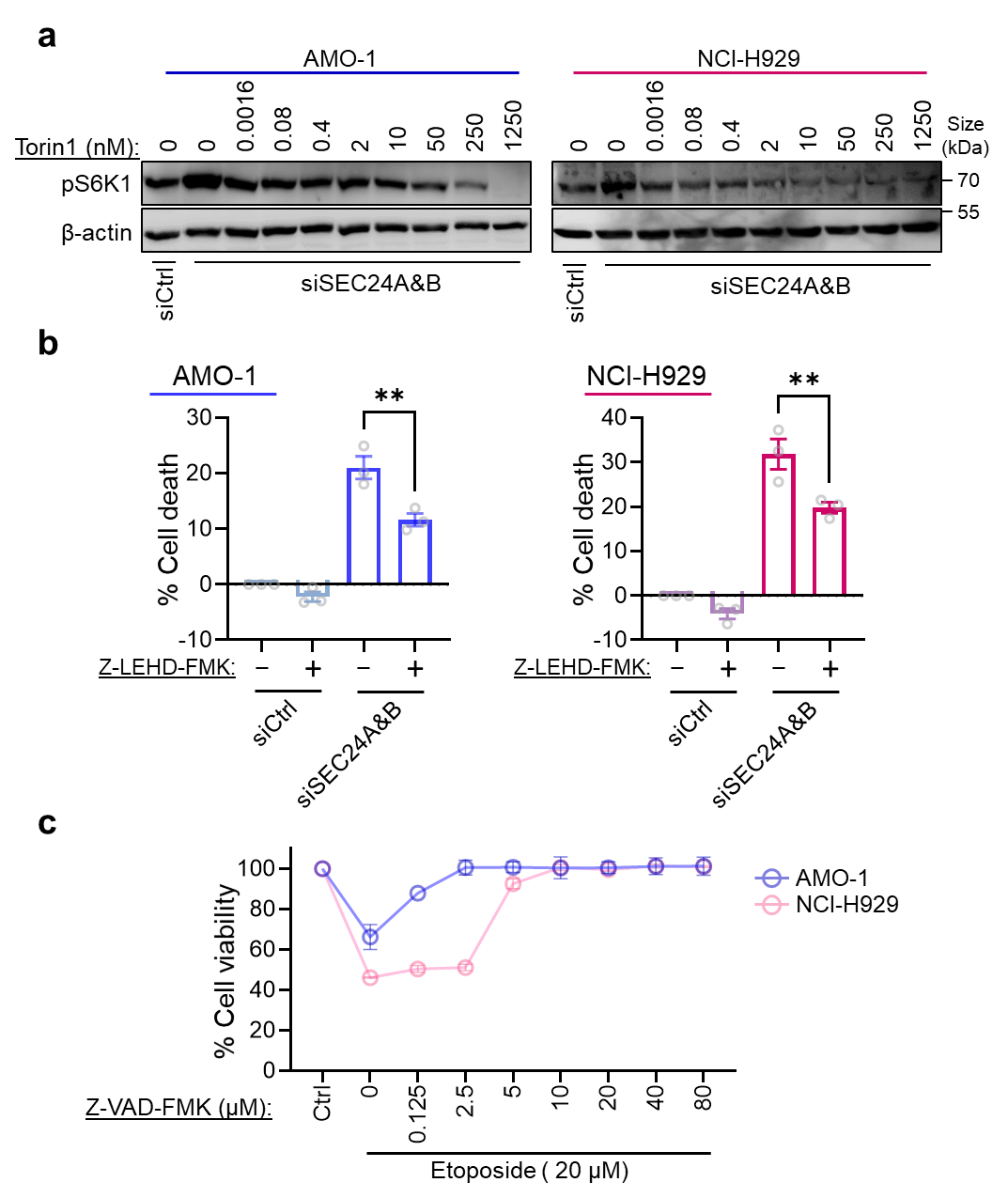


Figure S18. a Representative immunoblots showing the effects of increasing concentrations of Torin-1 in SEC24A&B co-depleted (siSEC24A&B) AMO-1 and NCI-H929 cells compared with control cells (siCtrl). b AMO-1 and NCI-H929 cells were treated with Z-LEHD-FMK (2 µM) or DMSO during the final 16 h of a 48-h SEC24A&B or control siRNA transfection. Cell viability was assessed by CellTiter-Glo. Data are presented as mean ± SEM from three independent experiments. Statistical significance was determined by one-way ANOVA with Tukey’s multiple-comparison test (**P < 0.01). c Dose–response curves of AMO-1 and NCI-H929 cells treated with increasing concentrations of Z-VAD-FMK. Cells were exposed to etoposide (20 µM) for 16 h in the presence of the indicated concentrations of Z-VAD-FMK, and cell viability was measured by CellTiter-Glo (n = 3 independent experiments).


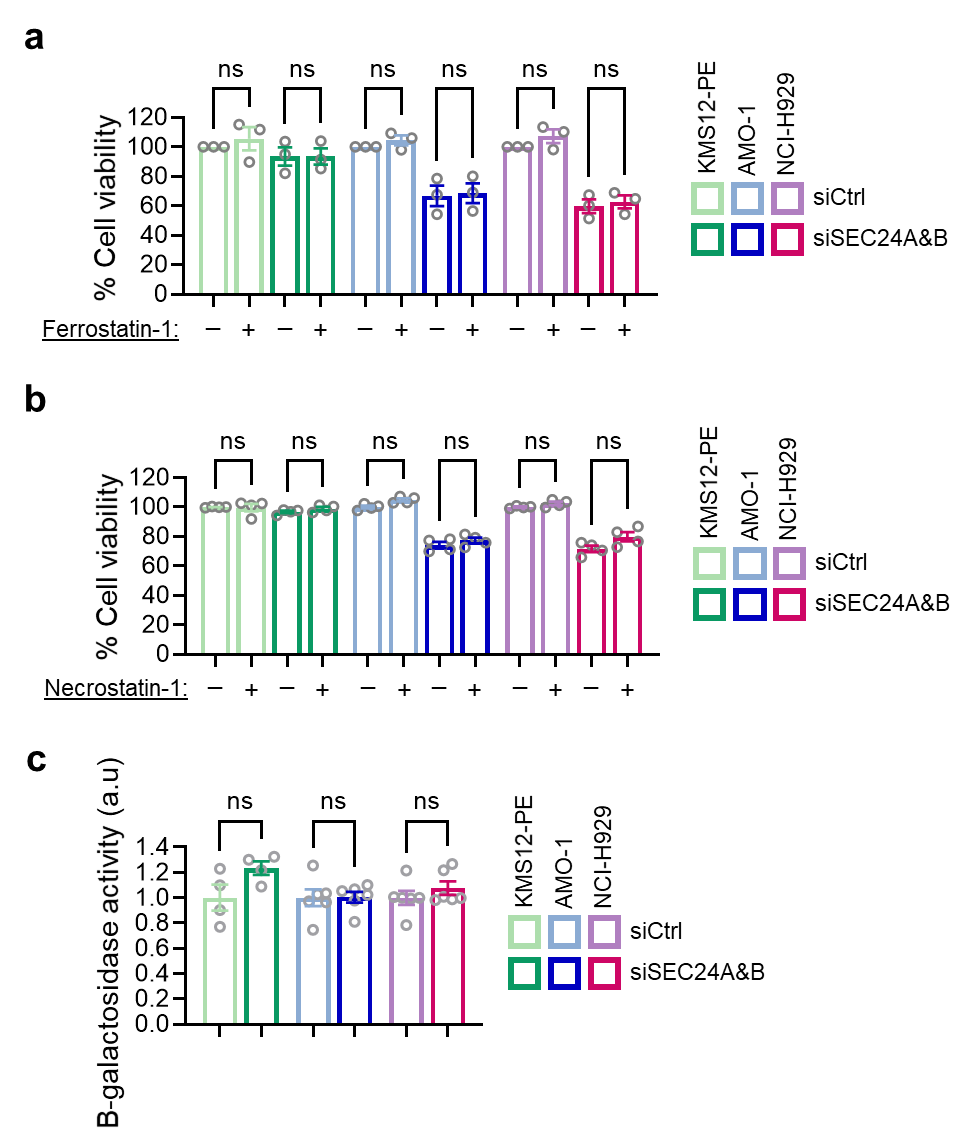


**Figure S19.** Ferroptosis, necroptosis and senescence failed to show appreciable activities in SEC24A&B-depleted MM cell lines. KMS-12PE (green), AMO-1 (blue), and NCI-H929 (pink) MM cell lines were transfected with non-targeting siRNAs (Ctrl) or SEC24A&B siRNAs, and further treated with the anti-ferroptosis compound ferrostatin-1 (1 μM) or **b** anti-necroptosis compound necrostatin-1 (30 μM) for 24 hours. At the end of the total 48 hours of siRNA-mediated silencing, cell viability was measured using the CellTiterGlo assay. **c** Senescence-associated β-galactosidase activity was evaluated using the Beta-Glo Assay System following siRNA-mediated silencing of SEC24A&B in MM cell lines for 48 hours. Each gray circle represents an independent experiment, and the data are shown as mean ± SEM; Student’s t-test, ns = nonsignificant.

**
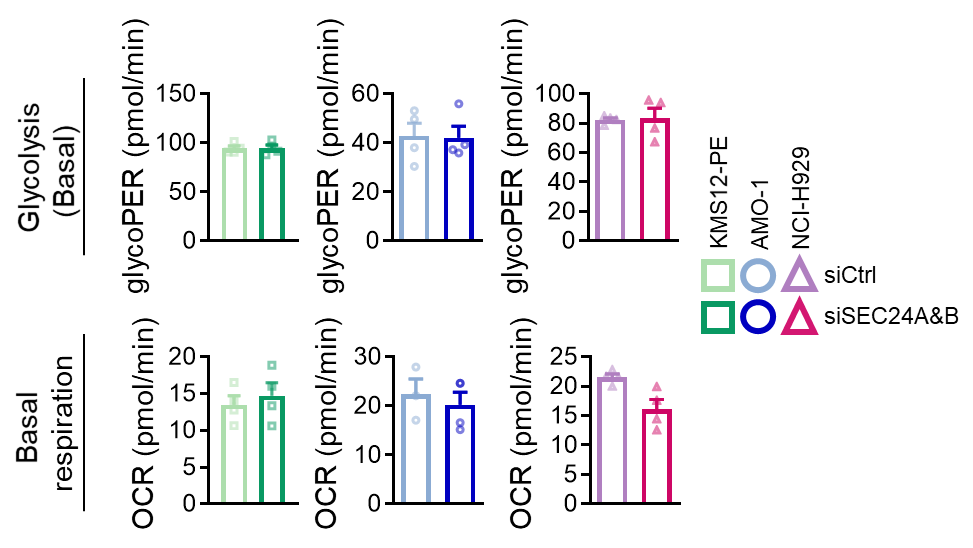
**

**Figure S20.** No striking differences in basal glycolysis and basal respiration were observed under knockdown conditions compared to controls. After 48 hours of control or SEC24A&B siRNA-mediated transfection, the glycolytic proton efflux rate (glycoPER), an indicator of glycolysis, and the oxygen consumption rate (OCR), an indicator of OXPHOS, were measured on a Seahorse XF96 Analyzer under basal conditions. Compared with controls, no significant differences were detected by Student’s t-test.


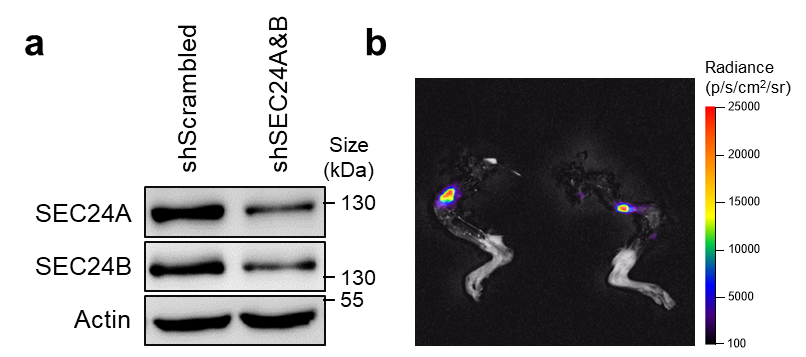


Figure S21. NCI-H929 cells expressing scrambled shRNA or SEC24A&B shRNA were incubated with 1 µg/mL doxycycline for 96 hours. a Immunoblots validating knockdown of SEC24A and SEC24B paralogs. b At the end of the experiment, mice were sacrificed and hind limbs were dissected. Representative bioluminescence images of hind limbs from a mouse bearing scrambled shRNA show femoral localization of myeloma cells.


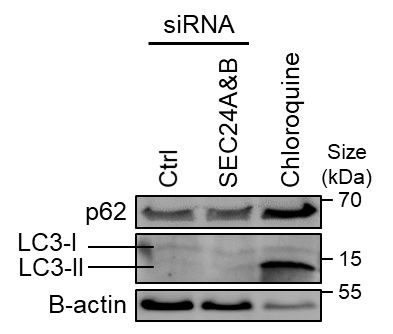


Figure S22. Blocking ER export did not affect autophagic activity. Protein expression of p62 and LC3 in AMO-1 cells, following the silencing of SEC24A&B, was monitored by immunoblotting. Cells were treated with the lysosomal inhibitor chloroquine (50 μM) for 16 hours as a positive control. Increased levels of p62 and LC3-II indicate either enhanced autophagic flux or a fusion defect between autophagosomes and lysosomes.


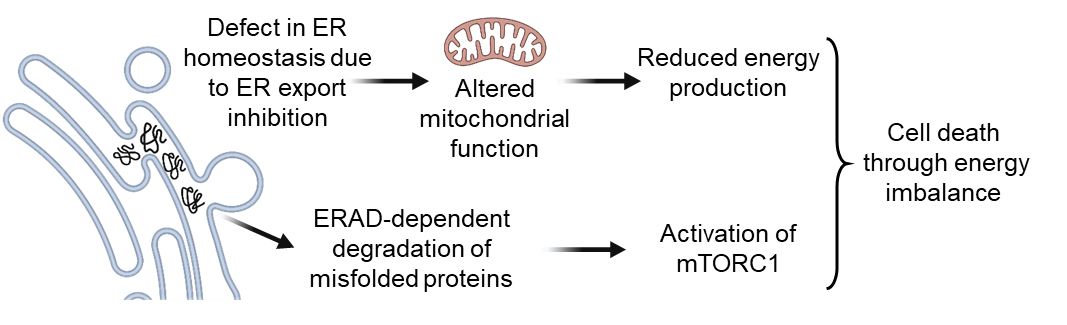


Figure S23. Schematic demonstration of the major findings. Icons were drawn using BioRender.

Table S1. Secretory status of MM cell lines.

| Cell line | Secretion type |
| --- | --- |
| KMS-12PE | Non-secretory |
| VOLIN | IgG |
| OH-2 | IgG |
| LP-1 | IgG |
| KJON | FLC |
| URVIN | IgG |
| FOLE | IgA/FLC |
| AMO-1 | IgA |
| NCI-H929 | IgA |

**Table S2.** MM patient samples used in the study.

| Sample number | Age/  Sex | M protein isotype | Best response | BM at biopsy | MM activity | Line of therapy |
| --- | --- | --- | --- | --- | --- | --- |
| 1 | 56/M | FLC λ | CR | 2% clonal PCs | PD (EMM, Bone) | 3 |
| 2 | 68/F | FLC κ | MRDneg. sCR | MRD neg. CR | CR | 1 |
| 3 | 71/M | IgA κ | CR | 5.6% clonal PCs | PD | 7 |
| 4 | 56/F | IgG κ | sCR | 0,0037% clonal PCs | MRDpos. sCR | 1 |
| 5 | 57/F | IgG κ | sCR | 42% clonal PCs | treatment naive at biopsy! | 0 |
| 6 | 50/M | FLC λ | sCR | 30% clonal PCs | treatment naive at biopsy! | 0 |
| 7 | 65/M | FLC κ | MRDneg. sCR | 10,5% clonal PCs | treatment naive at biopsy! | 0 |
| 8 | 48/F | IgA κ | MRDneg. sCR | 14% clonal PCs | treatment naive at biopsy! | 0 |
| 9 | 68/F | FLC λ | MRDpos sCR | 17,5% clonal PCs | PD | 1 |
| 10 | 82/M | FLC κ | VGPR | 33% clonal PCs | treatment naive at biopsy! | 0 |
| 11 | 60/M | FLC κ | MRDpos sCR | 6,5% clonal PCs | PD | 0 |
| 12 | 55/F | FLC λ | MRDneg. sCR | MRDneg. sCR | MRDneg. sCR | 1 |
| 13 | 63/F | IgA λ | MRDneg. sCR | MRDneg. sCR | MRDneg. sCR | 1 |
| 14 | 74/F | IgG λ | VGPR | 31% clonal PCs | treatment naive at biopsy! | 0 |
| 15 | 82/M | FLC λ | VGPR | 53% clonal PCs | treatment naive at biopsy! | 0 |
| 16 | 72/M | IgG κ | MRDneg. sCR | MRDneg. sCR | MRDneg. sCR | 1 |
| 17 | 71/F | IgG κ | NA | no clonal PCs | NA | 0 |

Sample number 17: Monoclonal gammopathy of undetermined significance.

F, female; M, male; MRD, minimal residual disease; CR, complete response; sCR, stringent complete response; VGPR, very good partial response; EMM, extramedullary myeloma; PC, plasma cell; PD, progressive disease; NA, not applicable.

Uncropped Images of Western Blots


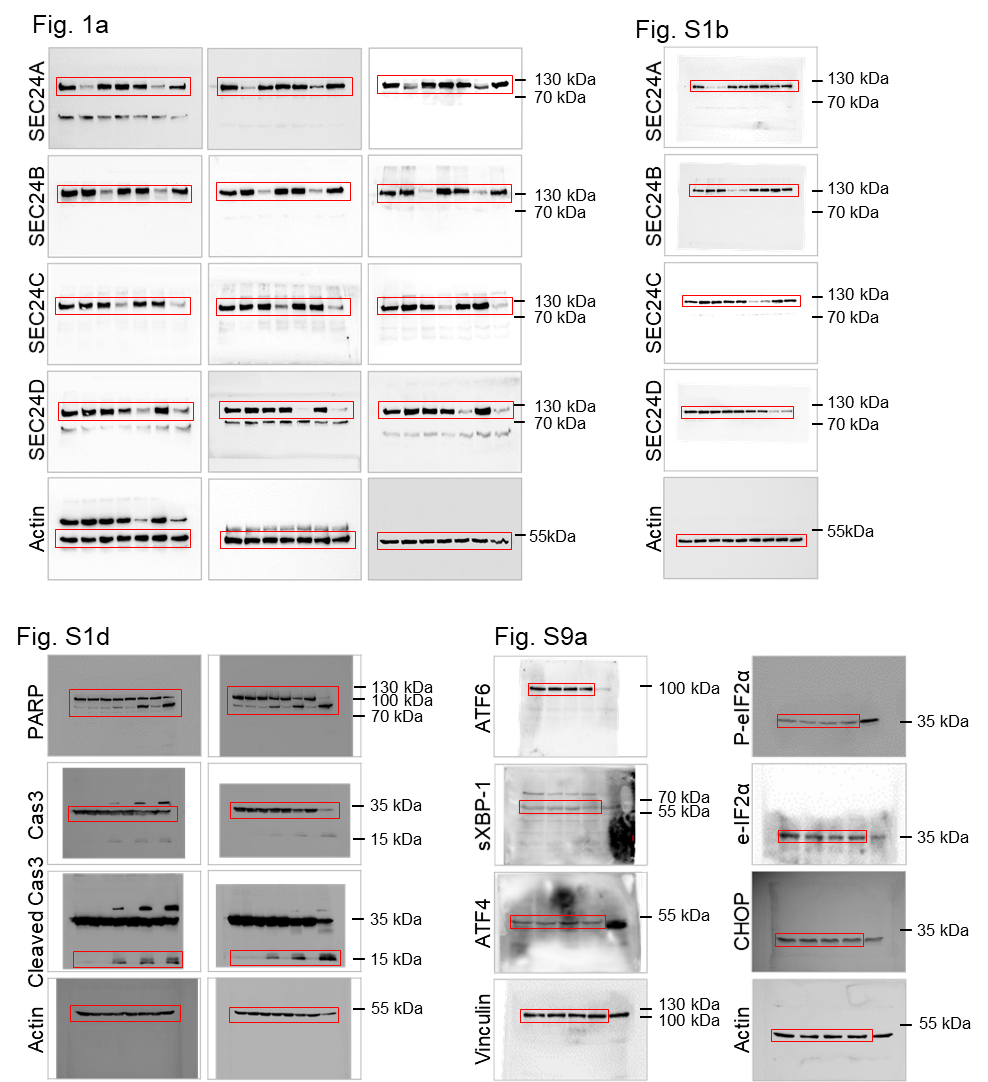


Uncropped Images of Western Blots


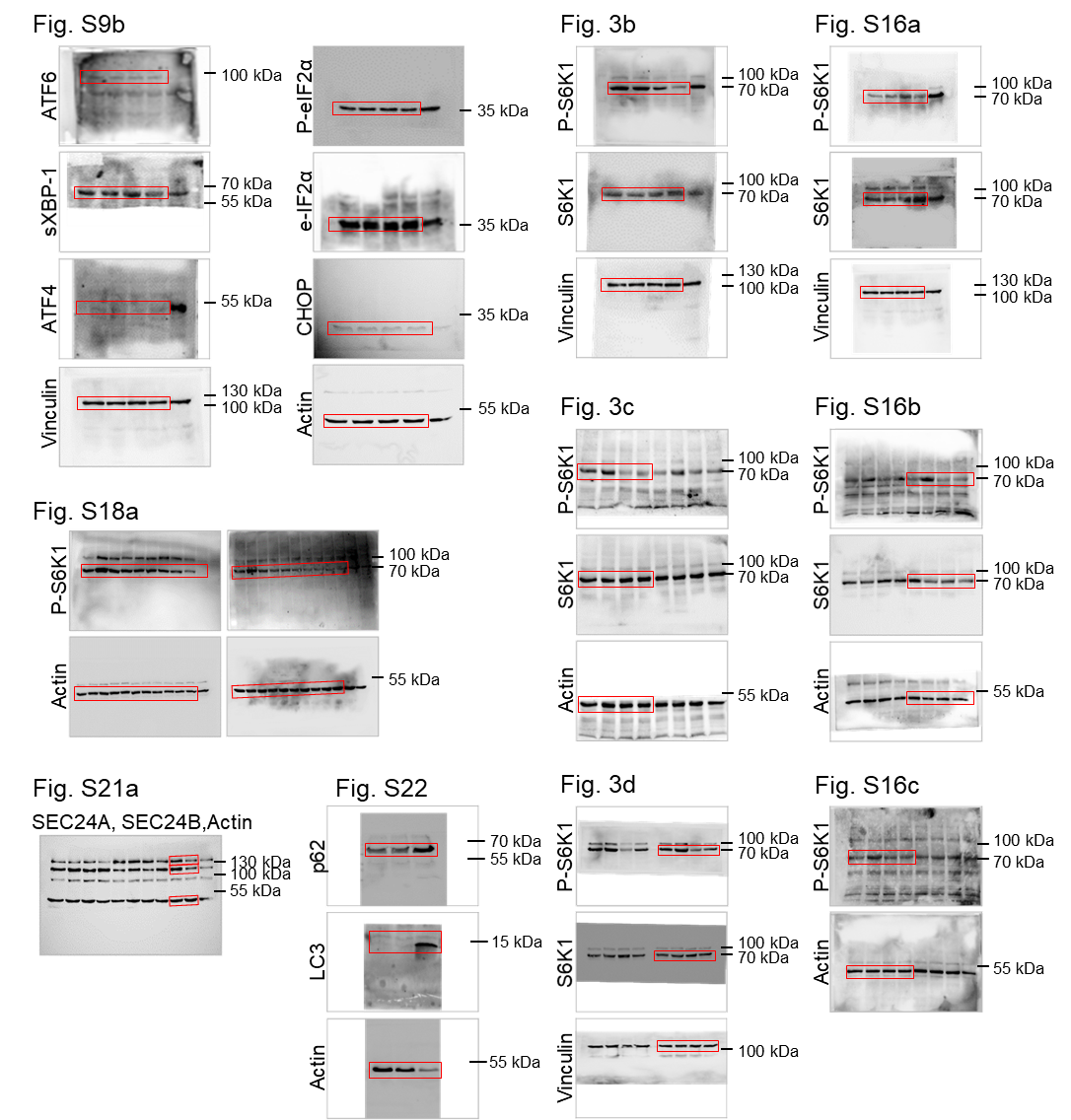


**KEY RESOURCES**

| **REAGENT** | **SOURCE** | **IDENTIFIER** |
| --- | --- | --- |
| Rabbit SEC24A antibody | Invitrogen | #PA5-66043 |
| Rabbit SEC24B antibody | Bethyl Laboratories | #A304-876A |
| Rabbit SEC24C antibody | Bethyl Laboratories | #A304-759A |
| Rabbit SEC24D antibody | Bethyl Laboratories | #A304-813A |
| Rabbit SEC16A antibody | Bethyl Laboratories | #A300-648A |
| Rabbit SEC16B antibody | Abnova | #PAB19201 |
| Mouse SEC31A antibody | BD biosciences | #612351 |
| Rabbit Calnexin antibody | Abcam | #ab22595 |
| Rabbit SEC24C antibody | Cell signaling | #14676 |
| Rabbit SEC24B antibody | Cell signaling | #12042 |
| Rat ATF6 antibody | Biolegend | #853102 |
| Mouse sXBP-1 antibody | Biolegend | #647502 |
| Rabbit ATF4 antibody | Cell signaling | #11815 |
| Mouse Vinculin antibody | Sigma-Aldrich | #SAB4200729 |
| Mouse Beta Actin antibody | Santa Cruz Biotechnology | #sc-47778 |
| Rabbit p70 S6 kinase antibody | Cell signaling | #9202 |
| Rabbit phospho-p70 S6 kinase antibody | Cell signaling | #9205 |
| Mouse Puromycin antibody | Biolegend | # 381508 |
| Mouse SQSTM1/p62 antibody | Abnova | #K7241-2C11 |
| Rabbit LC3 antibody | MBL | #PM036 |
| Mouse CD138 antibody | Biolegend | #356531 |
| Mouse CD19 antibody | Biolegend | #302207 |
| Mouse CD56 antibody | Biolegend | #318303 |
| Goat anti-Human IgG/IgA/IgM Secondary Antibody, TRITC | Invitrogen | #A18852 |
| Goat anti-Human IgG/IgA/IgM Secondary Antibody, FITC | Invitrogen | #A18848 |
| Mouse CHOP antibody | Cell signaling | # 2895S |
| Rabbit phospho-eIF2α (Ser51) antibody | Cell signaling | # 3597S |
| Mouse eIF2α antibody | Cell signaling | #2103 |
| Rabbit Ubiquitin antibody | Cell signaling | #43124 |
| Caspase 3 | Cell signaling | #9662 |
| PARP | Cell signaling | #9542 |
| Goat anti-Mouse IgG (H+L) Secondary Antibody, Alexa Fluor 488 | Invitrogen | #A-11001 |
| Goat anti-Mouse IgG (H+L) Secondary Antibody, Alexa Fluor 568 | Invitrogen | #A-11031 |
| Goat anti-Mouse IgG (H+L) Secondary Antibody, Alexa Fluor 647 | Invitrogen | #A-21235 |
| Goat anti-Rabbit IgG (H+L) Secondary Antibody, Alexa Fluor 488 | Invitrogen | #A-11008 |
| Goat anti-Rabbit IgG (H+L) Secondary Antibody, Alexa Fluor 568 | Invitrogen | #A-11011 |
| Goat anti-Rabbit IgG (H+L) Secondary Antibody, Alexa Fluor 647 | Invitrogen | #A-21244 |
| CD138 MicroBeads | Miltenyi Biotec | #130-051-301 |
| CellTiter-Glo 2.0 Cell Viability Assay kit | Promega | #G9242 |
| Glutamine/Glutamate-Glo Assay | Promega | #J8021 |
| Annexin V Apoptosis Detection Kit with 7-AAD | Biolegend | #640930 |
| SimpleStep GFP ELISA kit | Abcam | #ab171581 |
| Goat Anti-Human IgG+IgM+IgA H&L ELISA kit | Abcam | #ab102416 |
| Beta-Glo Assay kit | Promega | #E4720 |
| Cytofix/Cytoperm Fixation/Permeabilization Kit | BD biosciences | # 554714 |
| Seahorse XF Glycolytic Rate Assay Kit | Agilent | #103346-100 |
| Seahorse XF Cell Mito Stress Test Kit | Agilent | #103010-100 |
| Seahorse XF Media, Supplements & Calibrant | Agilent | #103010-100 |
| Seahorse FluxPaks | Agilent | #103022-100 |
| May-Grunwald's eosin methylene blue | Merck Millipore | #101352 |
| Giemsa's azur-eosin-methylene blue | Merck Millipore | #109203 |
| JC-1 | Enzo | #ENZ-52304 |
| Bradford Protein Assay Dye | Bio-Rad | #5000006 |
| TPE-MI | MedChemExpress | #HY-143218 |
| HiPerFect Transfection Reagent | Qiagen | #301707 |
| PEI Transfection Reagent | MedChemExpress | #HY-K2014 |
| Polybrene | MedChemExpress | #HY-112735 |
| Cycloheximide | Sigma-Aldrich | #239765 |
| Puromycin | InvivoGen | #ant-pr-1 |
| Bortezomib | Selleckchem | #S1013 |
| Lenalidomide | MedChemExpress | #HY-A0003 |
| Torin 1 | MedChemExpress | #HY-13003 |
| Kifunensine | MedChemExpress | #HY-19332 |
| STF-083010 | MedChemExpress | #HY-15845 |
| KIRA-6 | MedChemExpress | #HY-19708 |
| Z-VAD-FMK | Selleckchem | #S7023 |
| Z-LEHD-FMK | MedChemExpress | #HY-P1010 |
| Brefeldin A | Sigma-Aldrich | #B6542 |
| MG132 | Selleckchem | #S2619 |
| Carfilzomib | Cayman Chemical | #17554 |
| Etoposide | Sigma-Aldrich | #E1383 |
| Necrostatin-1 | MedChemExpress | # HY-15760 |
| Ferrostatin-1 | MedChemExpress | #HY-100579 |
| Chloroquine | MedChemExpress | #HY-17589A |
| Protease and Phosphatase Inhibitor | Thermo Scientific | #A32961 |
| Matrigel | Corning | #356230 |
| Fetal Bovine Serum | Gibco | #A5256701 |
| Penicillin-Streptomycin | Gibco | #15070063 |
| L-Glutamine | Gibco | #25030149 |
| RPMI 1640 Medium | Gibco | #21870076 |
| L-15 Medium | Sigma-Aldrich | #L1518 |
| pCMV-VSV-G | Addgene | #8454 |
| psPAX2 | Addgene | #12260 |
| pHR GFP-CaaX | Addgene | #113020 |
| Triton X-100 | Sigma-Aldrich | #X100 |
| Paraformaldehyde | Sigma-Aldrich | #158127 |
| Polyvinyl alcohol mounting medium with DABCO | Sigma-Aldrich | #10981 |
| PAGE gel solution | Carl Roth | #3029.1 |
| TEMED | Sigma-Aldrich | #T9281 |
| Ammonium persulfate | Sigma-Aldrich | #A3678 |
| Dimethyl sulfoxide | Sigma-Aldrich | #34869 |
| Sodium dodecyl sulfate | Carl Roth | #CN30.2 |
| Sodium deoxycholate monohydrate | Thermo Scientific | #B20759 |
| Sodium chloride | Carl Roth | #P029.3 |
| TRIS | Carl Roth | #4855.3 |
| Tween 20 | Thermo Scientific | #A85115 |
| White Leghorn chicken eggs | Schropper | #BeLSL |
| Doxycycline hyclate | MedChemExpress | #HY-N0565B |
| CycLuc1 | MedChemExpress | #HY-111653 |

**siRNAs**

| **Target** | **Sense strand (5' - 3')** | **Anti-sense strand (5' - 3')** |
| --- | --- | --- |
| NegCtrl_#1 | CTTCAACGCCACCTACGTCGA | TCGACGTAGGTGGCGTTGAAG |
| NegCtrl_#2 | UGUUCUUAGGCCCAUUUCAGA | UCUGAAAUGGGCCUAAGAACA |
| NegCtrl_#3 | CTTCAACGCCACCTACGTCGA | TCGACGTAGGTGGCGTTGAAG |
| SEC24A_#1 | CUUUGAAUUGGCAAUAUAACU | AGUUAUAUUGCCAAUUCAAAG |
| SEC24A_#2 | CAGUUAAAGAUAAUUCAUUCU | AGAAUGAAUUAUCUUUAACUG |
| SEC24B_#1 | CAAACGUGUGGAUGCUUAUGA | UCAUAAGCAUCCACACGUUUG |
| SEC24B_#2 | GAGGAACCCUUUACAAAUACA | UGUAUUUGUAAAGGGUUCCUC |
| SEC24C_#1 | GGAAAUACAAGUUUAACCACA | UGUGGUUAAACUUGUAUUUCC |
| SEC24C_#2 | UGAUUCAUCCCAACUUAUACA | UGUAUAAGUUGGGAUGAAUCA |
| SEC24D_#1 | AGGUGCUCCACUUCUAUAAUG | CAUUAUAGAAGUGGAGCACCU |
| SEC24D_#2 | CAAAAGAGGCCAUAUUCAAUG | CAUUGAAUAUGGCCUCUUUUG |

**EXTENDED STATISTICS**

**Results of statistical analyses for Figure 1b**

| **2way ANOVA Comparisons** | **Summary** | **P Value** |
| --- | --- | --- |
| siCtrl_ AMO1 vs. siCtrl_ KMS-12PE | ns | 0.815 |
| siCtrl_ AMO1 vs. siCtrl_ NCI-H929 | ns | 0.992 |
| siCtrl_ AMO1 vs. siSEC24A_ AMO1 | *** | <.001 |
| siCtrl_ AMO1 vs. siSEC24A_ KMS-12PE | ns | 0.606 |
| siCtrl_ AMO1 vs. siSEC24A_ NCI-H929 | *** | <.001 |
| siCtrl_ AMO1 vs. siSEC24B_ AMO1 | ** | 0.002 |
| siCtrl_ AMO1 vs. siSEC24B_ KMS-12PE | ns | 0.947 |
| siCtrl_ AMO1 vs. siSEC24B_ NCI-H929 | *** | <.001 |
| siCtrl_ AMO1 vs. siSEC24C_ AMO1 | ** | 0.006 |
| siCtrl_ AMO1 vs. siSEC24C_ KMS-12PE | ns | 0.645 |
| siCtrl_ AMO1 vs. siSEC24C_ NCI-H929 | *** | <.001 |
| siCtrl_ AMO1 vs. siSEC24D_ AMO1 | * | 0.017 |
| siCtrl_ AMO1 vs. siSEC24D_ KMS-12PE | ns | 0.938 |
| siCtrl_ AMO1 vs. siSEC24D_ NCI-H929 | *** | <.001 |
| siCtrl_ AMO1 vs. siSEC24A&B_ AMO1 | *** | <.001 |
| siCtrl_ AMO1 vs. siSEC24A&B_ KMS-12PE | ns | 0.051 |
| siCtrl_ AMO1 vs. siSEC24A&B_ NCI-H929 | *** | <.001 |
| siCtrl_ AMO1 vs. siSEC24A&C_ AMO1 | *** | <.001 |
| siCtrl_ AMO1 vs. siSEC24A&C_ KMS-12PE | * | 0.041 |
| siCtrl_ AMO1 vs. siSEC24A&C_ NCI-H929 | *** | <.001 |
| siCtrl_ AMO1 vs. siSEC24A&D_ AMO1 | *** | <.001 |
| siCtrl_ AMO1 vs. siSEC24A&D_ KMS-12PE | * | 0.031 |
| siCtrl_ AMO1 vs. siSEC24A&D_ NCI-H929 | *** | <.001 |
| siCtrl_ AMO1 vs. siSEC24B&C_ AMO1 | *** | <.001 |
| siCtrl_ AMO1 vs. siSEC24B&C_ KMS-12PE | * | 0.035 |
| siCtrl_ AMO1 vs. siSEC24B&C_ NCI-H929 | *** | <.001 |
| siCtrl_ AMO1 vs. siSEC24B&D_ AMO1 | *** | <.001 |
| siCtrl_ AMO1 vs. siSEC24B&D_ KMS-12PE | ns | 0.583 |
| siCtrl_ AMO1 vs. siSEC24B&D_ NCI-H929 | *** | <.001 |
| siCtrl_ AMO1 vs. siSEC24C&D_ AMO1 | *** | <.001 |
| siCtrl_ AMO1 vs. siSEC24C&D_ KMS-12PE | ** | 0.001 |
| siCtrl_ AMO1 vs. siSEC24C&D_ NCI-H929 | *** | <.001 |
| siCtrl_ KMS-12PE vs. siCtrl_ NCI-H929 | ns | 0.814 |
| siCtrl_ KMS-12PE vs. siSEC24A_ AMO1 | *** | <.001 |
| siCtrl_ KMS-12PE vs. siSEC24A_ KMS-12PE | ns | 0.516 |
| siCtrl_ KMS-12PE vs. siSEC24A_ NCI-H929 | *** | <.001 |
| siCtrl_ KMS-12PE vs. siSEC24B_ AMO1 | ** | 0.005 |
| siCtrl_ KMS-12PE vs. siSEC24B_ KMS-12PE | ns | 0.885 |
| siCtrl_ KMS-12PE vs. siSEC24B_ NCI-H929 | *** | <.001 |
| siCtrl_ KMS-12PE vs. siSEC24C_ AMO1 | * | 0.013 |
| siCtrl_ KMS-12PE vs. siSEC24C_ KMS-12PE | ns | 0.547 |
| siCtrl_ KMS-12PE vs. siSEC24C_ NCI-H929 | *** | <.001 |
| siCtrl_ KMS-12PE vs. siSEC24D_ AMO1 | * | 0.029 |
| siCtrl_ KMS-12PE vs. siSEC24D_ KMS-12PE | ns | 0.787 |
| siCtrl_ KMS-12PE vs. siSEC24D_ NCI-H929 | *** | <.001 |
| siCtrl_ KMS-12PE vs. siSEC24A&B_ AMO1 | *** | <.001 |
| siCtrl_ KMS-12PE vs. siSEC24A&B_ KMS-12PE | ns | 0.058 |
| siCtrl_ KMS-12PE vs. siSEC24A&B_ NCI-H929 | *** | <.001 |
| siCtrl_ KMS-12PE vs. siSEC24A&C_ AMO1 | *** | <.001 |
| siCtrl_ KMS-12PE vs. siSEC24A&C_ KMS-12PE | * | 0.049 |
| siCtrl_ KMS-12PE vs. siSEC24A&C_ NCI-H929 | *** | <.001 |
| siCtrl_ KMS-12PE vs. siSEC24A&D_ AMO1 | *** | <.001 |
| siCtrl_ KMS-12PE vs. siSEC24A&D_ KMS-12PE | * | 0.039 |
| siCtrl_ KMS-12PE vs. siSEC24A&D_ NCI-H929 | *** | <.001 |
| siCtrl_ KMS-12PE vs. siSEC24B&C_ AMO1 | *** | <.001 |
| siCtrl_ KMS-12PE vs. siSEC24B&C_ KMS-12PE | * | 0.042 |
| siCtrl_ KMS-12PE vs. siSEC24B&C_ NCI-H929 | *** | <.001 |
| siCtrl_ KMS-12PE vs. siSEC24B&D_ AMO1 | *** | <.001 |
| siCtrl_ KMS-12PE vs. siSEC24B&D_ KMS-12PE | ns | 0.498 |
| siCtrl_ KMS-12PE vs. siSEC24B&D_ NCI-H929 | *** | <.001 |
| siCtrl_ KMS-12PE vs. siSEC24C&D_ AMO1 | ** | 0.002 |
| siCtrl_ KMS-12PE vs. siSEC24C&D_ KMS-12PE | ** | 0.002 |
| siCtrl_ KMS-12PE vs. siSEC24C&D_ NCI-H929 | *** | <.001 |
| siCtrl_ NCI-H929 vs. siSEC24A_ AMO1 | *** | <.001 |
| siCtrl_ NCI-H929 vs. siSEC24A_ KMS-12PE | ns | 0.624 |
| siCtrl_ NCI-H929 vs. siSEC24A_ NCI-H929 | *** | <.001 |
| siCtrl_ NCI-H929 vs. siSEC24B_ AMO1 | ** | 0.003 |
| siCtrl_ NCI-H929 vs. siSEC24B_ KMS-12PE | ns | 0.942 |
| siCtrl_ NCI-H929 vs. siSEC24B_ NCI-H929 | *** | <.001 |
| siCtrl_ NCI-H929 vs. siSEC24C_ AMO1 | ** | 0.009 |
| siCtrl_ NCI-H929 vs. siSEC24C_ KMS-12PE | ns | 0.661 |
| siCtrl_ NCI-H929 vs. siSEC24C_ NCI-H929 | *** | <.001 |
| siCtrl_ NCI-H929 vs. siSEC24D_ AMO1 | * | 0.024 |
| siCtrl_ NCI-H929 vs. siSEC24D_ KMS-12PE | ns | 0.946 |
| siCtrl_ NCI-H929 vs. siSEC24D_ NCI-H929 | *** | <.001 |
| siCtrl_ NCI-H929 vs. siSEC24A&B_ AMO1 | *** | <.001 |
| siCtrl_ NCI-H929 vs. siSEC24A&B_ KMS-12PE | ns | 0.059 |
| siCtrl_ NCI-H929 vs. siSEC24A&B_ NCI-H929 | *** | <.001 |
| siCtrl_ NCI-H929 vs. siSEC24A&C_ AMO1 | *** | <.001 |
| siCtrl_ NCI-H929 vs. siSEC24A&C_ KMS-12PE | * | 0.049 |
| siCtrl_ NCI-H929 vs. siSEC24A&C_ NCI-H929 | *** | <.001 |
| siCtrl_ NCI-H929 vs. siSEC24A&D_ AMO1 | *** | <.001 |
| siCtrl_ NCI-H929 vs. siSEC24A&D_ KMS-12PE | * | 0.038 |
| siCtrl_ NCI-H929 vs. siSEC24A&D_ NCI-H929 | *** | <.001 |
| siCtrl_ NCI-H929 vs. siSEC24B&C_ AMO1 | *** | <.001 |
| siCtrl_ NCI-H929 vs. siSEC24B&C_ KMS-12PE | * | 0.041 |
| siCtrl_ NCI-H929 vs. siSEC24B&C_ NCI-H929 | *** | <.001 |
| siCtrl_ NCI-H929 vs. siSEC24B&D_ AMO1 | *** | <.001 |
| siCtrl_ NCI-H929 vs. siSEC24B&D_ KMS-12PE | ns | 0.601 |
| siCtrl_ NCI-H929 vs. siSEC24B&D_ NCI-H929 | *** | <.001 |
| siCtrl_ NCI-H929 vs. siSEC24C&D_ AMO1 | *** | <.001 |
| siCtrl_ NCI-H929 vs. siSEC24C&D_ KMS-12PE | ** | 0.002 |
| siCtrl_ NCI-H929 vs. siSEC24C&D_ NCI-H929 | *** | <.001 |
| siSEC24A_ AMO1 vs. siSEC24A_ KMS-12PE | *** | <.001 |
| siSEC24A_ AMO1 vs. siSEC24A_ NCI-H929 | ns | 0.32 |
| siSEC24A_ AMO1 vs. siSEC24B_ AMO1 | ** | 0.002 |
| siSEC24A_ AMO1 vs. siSEC24B_ KMS-12PE | *** | <.001 |
| siSEC24A_ AMO1 vs. siSEC24B_ NCI-H929 | ns | 0.797 |
| siSEC24A_ AMO1 vs. siSEC24C_ AMO1 | *** | <.001 |
| siSEC24A_ AMO1 vs. siSEC24C_ KMS-12PE | *** | <.001 |
| siSEC24A_ AMO1 vs. siSEC24C_ NCI-H929 | ns | 0.741 |
| siSEC24A_ AMO1 vs. siSEC24D_ AMO1 | *** | <.001 |
| siSEC24A_ AMO1 vs. siSEC24D_ KMS-12PE | *** | <.001 |
| siSEC24A_ AMO1 vs. siSEC24D_ NCI-H929 | ns | 0.393 |
| siSEC24A_ AMO1 vs. siSEC24A&B_ AMO1 | *** | <.001 |
| siSEC24A_ AMO1 vs. siSEC24A&B_ KMS-12PE | ** | 0.002 |
| siSEC24A_ AMO1 vs. siSEC24A&B_ NCI-H929 | *** | <.001 |
| siSEC24A_ AMO1 vs. siSEC24A&C_ AMO1 | ns | 0.506 |
| siSEC24A_ AMO1 vs. siSEC24A&C_ KMS-12PE | ** | 0.002 |
| siSEC24A_ AMO1 vs. siSEC24A&C_ NCI-H929 | ns | 0.278 |
| siSEC24A_ AMO1 vs. siSEC24A&D_ AMO1 | ns | 0.51 |
| siSEC24A_ AMO1 vs. siSEC24A&D_ KMS-12PE | ** | 0.003 |
| siSEC24A_ AMO1 vs. siSEC24A&D_ NCI-H929 | * | 0.032 |
| siSEC24A_ AMO1 vs. siSEC24B&C_ AMO1 | ns | 0.804 |
| siSEC24A_ AMO1 vs. siSEC24B&C_ KMS-12PE | ** | 0.003 |
| siSEC24A_ AMO1 vs. siSEC24B&C_ NCI-H929 | ns | 0.26 |
| siSEC24A_ AMO1 vs. siSEC24B&D_ AMO1 | * | 0.025 |
| siSEC24A_ AMO1 vs. siSEC24B&D_ KMS-12PE | *** | <.001 |
| siSEC24A_ AMO1 vs. siSEC24B&D_ NCI-H929 | ** | 0.01 |
| siSEC24A_ AMO1 vs. siSEC24C&D_ AMO1 | ** | 0.007 |
| siSEC24A_ AMO1 vs. siSEC24C&D_ KMS-12PE | ns | 0.07 |
| siSEC24A_ AMO1 vs. siSEC24C&D_ NCI-H929 | *** | <.001 |
| siSEC24A_ KMS-12PE vs. siSEC24A_ NCI-H929 | *** | <.001 |
| siSEC24A_ KMS-12PE vs. siSEC24B_ AMO1 | * | 0.038 |
| siSEC24A_ KMS-12PE vs. siSEC24B_ KMS-12PE | ns | 0.614 |
| siSEC24A_ KMS-12PE vs. siSEC24B_ NCI-H929 | *** | <.001 |
| siSEC24A_ KMS-12PE vs. siSEC24C_ AMO1 | ns | 0.081 |
| siSEC24A_ KMS-12PE vs. siSEC24C_ KMS-12PE | ns | 0.962 |
| siSEC24A_ KMS-12PE vs. siSEC24C_ NCI-H929 | *** | <.001 |
| siSEC24A_ KMS-12PE vs. siSEC24D_ AMO1 | ns | 0.148 |
| siSEC24A_ KMS-12PE vs. siSEC24D_ KMS-12PE | ns | 0.705 |
| siSEC24A_ KMS-12PE vs. siSEC24D_ NCI-H929 | *** | <.001 |
| siSEC24A_ KMS-12PE vs. siSEC24A&B_ AMO1 | *** | <.001 |
| siSEC24A_ KMS-12PE vs. siSEC24A&B_ KMS-12PE | ns | 0.209 |
| siSEC24A_ KMS-12PE vs. siSEC24A&B_ NCI-H929 | *** | <.001 |
| siSEC24A_ KMS-12PE vs. siSEC24A&C_ AMO1 | *** | <.001 |
| siSEC24A_ KMS-12PE vs. siSEC24A&C_ KMS-12PE | ns | 0.183 |
| siSEC24A_ KMS-12PE vs. siSEC24A&C_ NCI-H929 | *** | <.001 |
| siSEC24A_ KMS-12PE vs. siSEC24A&D_ AMO1 | *** | <.001 |
| siSEC24A_ KMS-12PE vs. siSEC24A&D_ KMS-12PE | ns | 0.153 |
| siSEC24A_ KMS-12PE vs. siSEC24A&D_ NCI-H929 | *** | <.001 |
| siSEC24A_ KMS-12PE vs. siSEC24B&C_ AMO1 | *** | <.001 |
| siSEC24A_ KMS-12PE vs. siSEC24B&C_ KMS-12PE | ns | 0.163 |
| siSEC24A_ KMS-12PE vs. siSEC24B&C_ NCI-H929 | *** | <.001 |
| siSEC24A_ KMS-12PE vs. siSEC24B&D_ AMO1 | ** | 0.006 |
| siSEC24A_ KMS-12PE vs. siSEC24B&D_ KMS-12PE | ns | 0.977 |
| siSEC24A_ KMS-12PE vs. siSEC24B&D_ NCI-H929 | *** | <.001 |
| siSEC24A_ KMS-12PE vs. siSEC24C&D_ AMO1 | * | 0.017 |
| siSEC24A_ KMS-12PE vs. siSEC24C&D_ KMS-12PE | * | 0.016 |
| siSEC24A_ KMS-12PE vs. siSEC24C&D_ NCI-H929 | *** | <.001 |
| siSEC24A_ NCI-H929 vs. siSEC24B_ AMO1 | *** | <.001 |
| siSEC24A_ NCI-H929 vs. siSEC24B_ KMS-12PE | *** | <.001 |
| siSEC24A_ NCI-H929 vs. siSEC24B_ NCI-H929 | ns | 0.232 |
| siSEC24A_ NCI-H929 vs. siSEC24C_ AMO1 | *** | <.001 |
| siSEC24A_ NCI-H929 vs. siSEC24C_ KMS-12PE | *** | <.001 |
| siSEC24A_ NCI-H929 vs. siSEC24C_ NCI-H929 | ns | 0.225 |
| siSEC24A_ NCI-H929 vs. siSEC24D_ AMO1 | *** | <.001 |
| siSEC24A_ NCI-H929 vs. siSEC24D_ KMS-12PE | *** | <.001 |
| siSEC24A_ NCI-H929 vs. siSEC24D_ NCI-H929 | ns | 0.939 |
| siSEC24A_ NCI-H929 vs. siSEC24A&B_ AMO1 | *** | <.001 |
| siSEC24A_ NCI-H929 vs. siSEC24A&B_ KMS-12PE | *** | <.001 |
| siSEC24A_ NCI-H929 vs. siSEC24A&B_ NCI-H929 | *** | <.001 |
| siSEC24A_ NCI-H929 vs. siSEC24A&C_ AMO1 | ns | 0.718 |
| siSEC24A_ NCI-H929 vs. siSEC24A&C_ KMS-12PE | *** | <.001 |
| siSEC24A_ NCI-H929 vs. siSEC24A&C_ NCI-H929 | ns | 0.883 |
| siSEC24A_ NCI-H929 vs. siSEC24A&D_ AMO1 | ns | 0.714 |
| siSEC24A_ NCI-H929 vs. siSEC24A&D_ KMS-12PE | *** | <.001 |
| siSEC24A_ NCI-H929 vs. siSEC24A&D_ NCI-H929 | ns | 0.236 |
| siSEC24A_ NCI-H929 vs. siSEC24B&C_ AMO1 | ns | 0.448 |
| siSEC24A_ NCI-H929 vs. siSEC24B&C_ KMS-12PE | *** | <.001 |
| siSEC24A_ NCI-H929 vs. siSEC24B&C_ NCI-H929 | ns | 0.85 |
| siSEC24A_ NCI-H929 vs. siSEC24B&D_ AMO1 | ** | 0.002 |
| siSEC24A_ NCI-H929 vs. siSEC24B&D_ KMS-12PE | *** | <.001 |
| siSEC24A_ NCI-H929 vs. siSEC24B&D_ NCI-H929 | ns | 0.107 |
| siSEC24A_ NCI-H929 vs. siSEC24C&D_ AMO1 | *** | <.001 |
| siSEC24A_ NCI-H929 vs. siSEC24C&D_ KMS-12PE | * | 0.011 |
| siSEC24A_ NCI-H929 vs. siSEC24C&D_ NCI-H929 | ** | 0.007 |
| siSEC24B_ AMO1 vs. siSEC24B_ KMS-12PE | ** | 0.008 |
| siSEC24B_ AMO1 vs. siSEC24B_ NCI-H929 | ** | 0.008 |
| siSEC24B_ AMO1 vs. siSEC24C_ AMO1 | ns | 0.674 |
| siSEC24B_ AMO1 vs. siSEC24C_ KMS-12PE | * | 0.033 |
| siSEC24B_ AMO1 vs. siSEC24C_ NCI-H929 | * | 0.015 |
| siSEC24B_ AMO1 vs. siSEC24D_ AMO1 | ns | 0.43 |
| siSEC24B_ AMO1 vs. siSEC24D_ KMS-12PE | * | 0.012 |
| siSEC24B_ AMO1 vs. siSEC24D_ NCI-H929 | *** | <.001 |
| siSEC24B_ AMO1 vs. siSEC24A&B_ AMO1 | *** | <.001 |
| siSEC24B_ AMO1 vs. siSEC24A&B_ KMS-12PE | ns | 0.521 |
| siSEC24B_ AMO1 vs. siSEC24A&B_ NCI-H929 | *** | <.001 |
| siSEC24B_ AMO1 vs. siSEC24A&C_ AMO1 | *** | <.001 |
| siSEC24B_ AMO1 vs. siSEC24A&C_ KMS-12PE | ns | 0.579 |
| siSEC24B_ AMO1 vs. siSEC24A&C_ NCI-H929 | *** | <.001 |
| siSEC24B_ AMO1 vs. siSEC24A&D_ AMO1 | *** | <.001 |
| siSEC24B_ AMO1 vs. siSEC24A&D_ KMS-12PE | ns | 0.66 |
| siSEC24B_ AMO1 vs. siSEC24A&D_ NCI-H929 | *** | <.001 |
| siSEC24B_ AMO1 vs. siSEC24B&C_ AMO1 | ** | 0.001 |
| siSEC24B_ AMO1 vs. siSEC24B&C_ KMS-12PE | ns | 0.632 |
| siSEC24B_ AMO1 vs. siSEC24B&C_ NCI-H929 | *** | <.001 |
| siSEC24B_ AMO1 vs. siSEC24B&D_ AMO1 | ns | 0.393 |
| siSEC24B_ AMO1 vs. siSEC24B&D_ KMS-12PE | * | 0.041 |
| siSEC24B_ AMO1 vs. siSEC24B&D_ NCI-H929 | *** | <.001 |
| siSEC24B_ AMO1 vs. siSEC24C&D_ AMO1 | ns | 0.692 |
| siSEC24B_ AMO1 vs. siSEC24C&D_ KMS-12PE | ns | 0.47 |
| siSEC24B_ AMO1 vs. siSEC24C&D_ NCI-H929 | *** | <.001 |
| siSEC24B_ KMS-12PE vs. siSEC24B_ NCI-H929 | *** | <.001 |
| siSEC24B_ KMS-12PE vs. siSEC24C_ AMO1 | * | 0.021 |
| siSEC24B_ KMS-12PE vs. siSEC24C_ KMS-12PE | ns | 0.648 |
| siSEC24B_ KMS-12PE vs. siSEC24C_ NCI-H929 | *** | <.001 |
| siSEC24B_ KMS-12PE vs. siSEC24D_ AMO1 | * | 0.044 |
| siSEC24B_ KMS-12PE vs. siSEC24D_ KMS-12PE | ns | 0.9 |
| siSEC24B_ KMS-12PE vs. siSEC24D_ NCI-H929 | *** | <.001 |
| siSEC24B_ KMS-12PE vs. siSEC24A&B_ AMO1 | *** | <.001 |
| siSEC24B_ KMS-12PE vs. siSEC24A&B_ KMS-12PE | ns | 0.079 |
| siSEC24B_ KMS-12PE vs. siSEC24A&B_ NCI-H929 | *** | <.001 |
| siSEC24B_ KMS-12PE vs. siSEC24A&C_ AMO1 | *** | <.001 |
| siSEC24B_ KMS-12PE vs. siSEC24A&C_ KMS-12PE | ns | 0.068 |
| siSEC24B_ KMS-12PE vs. siSEC24A&C_ NCI-H929 | *** | <.001 |
| siSEC24B_ KMS-12PE vs. siSEC24A&D_ AMO1 | *** | <.001 |
| siSEC24B_ KMS-12PE vs. siSEC24A&D_ KMS-12PE | ns | 0.054 |
| siSEC24B_ KMS-12PE vs. siSEC24A&D_ NCI-H929 | *** | <.001 |
| siSEC24B_ KMS-12PE vs. siSEC24B&C_ AMO1 | *** | <.001 |
| siSEC24B_ KMS-12PE vs. siSEC24B&C_ KMS-12PE | ns | 0.059 |
| siSEC24B_ KMS-12PE vs. siSEC24B&C_ NCI-H929 | *** | <.001 |
| siSEC24B_ KMS-12PE vs. siSEC24B&D_ AMO1 | *** | <.001 |
| siSEC24B_ KMS-12PE vs. siSEC24B&D_ KMS-12PE | ns | 0.594 |
| siSEC24B_ KMS-12PE vs. siSEC24B&D_ NCI-H929 | *** | <.001 |
| siSEC24B_ KMS-12PE vs. siSEC24C&D_ AMO1 | ** | 0.003 |
| siSEC24B_ KMS-12PE vs. siSEC24C&D_ KMS-12PE | ** | 0.004 |
| siSEC24B_ KMS-12PE vs. siSEC24C&D_ NCI-H929 | *** | <.001 |
| siSEC24B_ NCI-H929 vs. siSEC24C_ AMO1 | ** | 0.002 |
| siSEC24B_ NCI-H929 vs. siSEC24C_ KMS-12PE | *** | <.001 |
| siSEC24B_ NCI-H929 vs. siSEC24C_ NCI-H929 | ns | 0.931 |
| siSEC24B_ NCI-H929 vs. siSEC24D_ AMO1 | *** | <.001 |
| siSEC24B_ NCI-H929 vs. siSEC24D_ KMS-12PE | *** | <.001 |
| siSEC24B_ NCI-H929 vs. siSEC24D_ NCI-H929 | ns | 0.293 |
| siSEC24B_ NCI-H929 vs. siSEC24A&B_ AMO1 | *** | <.001 |
| siSEC24B_ NCI-H929 vs. siSEC24A&B_ KMS-12PE | ** | 0.005 |
| siSEC24B_ NCI-H929 vs. siSEC24A&B_ NCI-H929 | *** | <.001 |
| siSEC24B_ NCI-H929 vs. siSEC24A&C_ AMO1 | ns | 0.373 |
| siSEC24B_ NCI-H929 vs. siSEC24A&C_ KMS-12PE | ** | 0.006 |
| siSEC24B_ NCI-H929 vs. siSEC24A&C_ NCI-H929 | ns | 0.203 |
| siSEC24B_ NCI-H929 vs. siSEC24A&D_ AMO1 | ns | 0.376 |
| siSEC24B_ NCI-H929 vs. siSEC24A&D_ KMS-12PE | ** | 0.008 |
| siSEC24B_ NCI-H929 vs. siSEC24A&D_ NCI-H929 | * | 0.022 |
| siSEC24B_ NCI-H929 vs. siSEC24B&C_ AMO1 | ns | 0.622 |
| siSEC24B_ NCI-H929 vs. siSEC24B&C_ KMS-12PE | ** | 0.008 |
| siSEC24B_ NCI-H929 vs. siSEC24B&C_ NCI-H929 | ns | 0.189 |
| siSEC24B_ NCI-H929 vs. siSEC24B&D_ AMO1 | ns | 0.06 |
| siSEC24B_ NCI-H929 vs. siSEC24B&D_ KMS-12PE | *** | <.001 |
| siSEC24B_ NCI-H929 vs. siSEC24B&D_ NCI-H929 | ** | 0.007 |
| siSEC24B_ NCI-H929 vs. siSEC24C&D_ AMO1 | * | 0.021 |
| siSEC24B_ NCI-H929 vs. siSEC24C&D_ KMS-12PE | ns | 0.123 |
| siSEC24B_ NCI-H929 vs. siSEC24C&D_ NCI-H929 | *** | <.001 |
| siSEC24C_ AMO1 vs. siSEC24C_ KMS-12PE | ns | 0.072 |
| siSEC24C_ AMO1 vs. siSEC24C_ NCI-H929 | ** | 0.005 |
| siSEC24C_ AMO1 vs. siSEC24D_ AMO1 | ns | 0.712 |
| siSEC24C_ AMO1 vs. siSEC24D_ KMS-12PE | * | 0.03 |
| siSEC24C_ AMO1 vs. siSEC24D_ NCI-H929 | *** | <.001 |
| siSEC24C_ AMO1 vs. siSEC24A&B_ AMO1 | *** | <.001 |
| siSEC24C_ AMO1 vs. siSEC24A&B_ KMS-12PE | ns | 0.765 |
| siSEC24C_ AMO1 vs. siSEC24A&B_ NCI-H929 | *** | <.001 |
| siSEC24C_ AMO1 vs. siSEC24A&C_ AMO1 | *** | <.001 |
| siSEC24C_ AMO1 vs. siSEC24A&C_ KMS-12PE | ns | 0.833 |
| siSEC24C_ AMO1 vs. siSEC24A&C_ NCI-H929 | *** | <.001 |
| siSEC24C_ AMO1 vs. siSEC24A&D_ AMO1 | *** | <.001 |
| siSEC24C_ AMO1 vs. siSEC24A&D_ KMS-12PE | ns | 0.923 |
| siSEC24C_ AMO1 vs. siSEC24A&D_ NCI-H929 | *** | <.001 |
| siSEC24C_ AMO1 vs. siSEC24B&C_ AMO1 | *** | <.001 |
| siSEC24C_ AMO1 vs. siSEC24B&C_ KMS-12PE | ns | 0.892 |
| siSEC24C_ AMO1 vs. siSEC24B&C_ NCI-H929 | *** | <.001 |
| siSEC24C_ AMO1 vs. siSEC24B&D_ AMO1 | ns | 0.203 |
| siSEC24C_ AMO1 vs. siSEC24B&D_ KMS-12PE | ns | 0.087 |
| siSEC24C_ AMO1 vs. siSEC24B&D_ NCI-H929 | *** | <.001 |
| siSEC24C_ AMO1 vs. siSEC24C&D_ AMO1 | ns | 0.415 |
| siSEC24C_ AMO1 vs. siSEC24C&D_ KMS-12PE | ns | 0.287 |
| siSEC24C_ AMO1 vs. siSEC24C&D_ NCI-H929 | *** | <.001 |
| siSEC24C_ KMS-12PE vs. siSEC24C_ NCI-H929 | *** | <.001 |
| siSEC24C_ KMS-12PE vs. siSEC24D_ AMO1 | ns | 0.134 |
| siSEC24C_ KMS-12PE vs. siSEC24D_ KMS-12PE | ns | 0.74 |
| siSEC24C_ KMS-12PE vs. siSEC24D_ NCI-H929 | *** | <.001 |
| siSEC24C_ KMS-12PE vs. siSEC24A&B_ AMO1 | *** | <.001 |
| siSEC24C_ KMS-12PE vs. siSEC24A&B_ KMS-12PE | ns | 0.192 |
| siSEC24C_ KMS-12PE vs. siSEC24A&B_ NCI-H929 | *** | <.001 |
| siSEC24C_ KMS-12PE vs. siSEC24A&C_ AMO1 | *** | <.001 |
| siSEC24C_ KMS-12PE vs. siSEC24A&C_ KMS-12PE | ns | 0.168 |
| siSEC24C_ KMS-12PE vs. siSEC24A&C_ NCI-H929 | *** | <.001 |
| siSEC24C_ KMS-12PE vs. siSEC24A&D_ AMO1 | *** | <.001 |
| siSEC24C_ KMS-12PE vs. siSEC24A&D_ KMS-12PE | ns | 0.14 |
| siSEC24C_ KMS-12PE vs. siSEC24A&D_ NCI-H929 | *** | <.001 |
| siSEC24C_ KMS-12PE vs. siSEC24B&C_ AMO1 | *** | <.001 |
| siSEC24C_ KMS-12PE vs. siSEC24B&C_ KMS-12PE | ns | 0.149 |
| siSEC24C_ KMS-12PE vs. siSEC24B&C_ NCI-H929 | *** | <.001 |
| siSEC24C_ KMS-12PE vs. siSEC24B&D_ AMO1 | ** | 0.005 |
| siSEC24C_ KMS-12PE vs. siSEC24B&D_ KMS-12PE | ns | 0.94 |
| siSEC24C_ KMS-12PE vs. siSEC24B&D_ NCI-H929 | *** | <.001 |
| siSEC24C_ KMS-12PE vs. siSEC24C&D_ AMO1 | * | 0.015 |
| siSEC24C_ KMS-12PE vs. siSEC24C&D_ KMS-12PE | * | 0.014 |
| siSEC24C_ KMS-12PE vs. siSEC24C&D_ NCI-H929 | *** | <.001 |
| siSEC24C_ NCI-H929 vs. siSEC24D_ AMO1 | ** | 0.002 |
| siSEC24C_ NCI-H929 vs. siSEC24D_ KMS-12PE | *** | <.001 |
| siSEC24C_ NCI-H929 vs. siSEC24D_ NCI-H929 | ns | 0.28 |
| siSEC24C_ NCI-H929 vs. siSEC24A&B_ AMO1 | *** | <.001 |
| siSEC24C_ NCI-H929 vs. siSEC24A&B_ KMS-12PE | ** | 0.009 |
| siSEC24C_ NCI-H929 vs. siSEC24A&B_ NCI-H929 | *** | <.001 |
| siSEC24C_ NCI-H929 vs. siSEC24A&C_ AMO1 | ns | 0.355 |
| siSEC24C_ NCI-H929 vs. siSEC24A&C_ KMS-12PE | * | 0.011 |
| siSEC24C_ NCI-H929 vs. siSEC24A&C_ NCI-H929 | ns | 0.197 |
| siSEC24C_ NCI-H929 vs. siSEC24A&D_ AMO1 | ns | 0.358 |
| siSEC24C_ NCI-H929 vs. siSEC24A&D_ KMS-12PE | * | 0.014 |
| siSEC24C_ NCI-H929 vs. siSEC24A&D_ NCI-H929 | * | 0.024 |
| siSEC24C_ NCI-H929 vs. siSEC24B&C_ AMO1 | ns | 0.581 |
| siSEC24C_ NCI-H929 vs. siSEC24B&C_ KMS-12PE | * | 0.013 |
| siSEC24C_ NCI-H929 vs. siSEC24B&C_ NCI-H929 | ns | 0.184 |
| siSEC24C_ NCI-H929 vs. siSEC24B&D_ AMO1 | ns | 0.093 |
| siSEC24C_ NCI-H929 vs. siSEC24B&D_ KMS-12PE | *** | <.001 |
| siSEC24C_ NCI-H929 vs. siSEC24B&D_ NCI-H929 | ** | 0.008 |
| siSEC24C_ NCI-H929 vs. siSEC24C&D_ AMO1 | * | 0.037 |
| siSEC24C_ NCI-H929 vs. siSEC24C&D_ KMS-12PE | ns | 0.161 |
| siSEC24C_ NCI-H929 vs. siSEC24C&D_ NCI-H929 | *** | <.001 |
| siSEC24D_ AMO1 vs. siSEC24D_ KMS-12PE | ns | 0.061 |
| siSEC24D_ AMO1 vs. siSEC24D_ NCI-H929 | *** | <.001 |
| siSEC24D_ AMO1 vs. siSEC24A&B_ AMO1 | *** | <.001 |
| siSEC24D_ AMO1 vs. siSEC24A&B_ KMS-12PE | ns | 0.997 |
| siSEC24D_ AMO1 vs. siSEC24A&B_ NCI-H929 | *** | <.001 |
| siSEC24D_ AMO1 vs. siSEC24A&C_ AMO1 | *** | <.001 |
| siSEC24D_ AMO1 vs. siSEC24A&C_ KMS-12PE | ns | 0.928 |
| siSEC24D_ AMO1 vs. siSEC24A&C_ NCI-H929 | *** | <.001 |
| siSEC24D_ AMO1 vs. siSEC24A&D_ AMO1 | *** | <.001 |
| siSEC24D_ AMO1 vs. siSEC24A&D_ KMS-12PE | ns | 0.837 |
| siSEC24D_ AMO1 vs. siSEC24A&D_ NCI-H929 | *** | <.001 |
| siSEC24D_ AMO1 vs. siSEC24B&C_ AMO1 | *** | <.001 |
| siSEC24D_ AMO1 vs. siSEC24B&C_ KMS-12PE | ns | 0.868 |
| siSEC24D_ AMO1 vs. siSEC24B&C_ NCI-H929 | *** | <.001 |
| siSEC24D_ AMO1 vs. siSEC24B&D_ AMO1 | ns | 0.102 |
| siSEC24D_ AMO1 vs. siSEC24B&D_ KMS-12PE | ns | 0.157 |
| siSEC24D_ AMO1 vs. siSEC24B&D_ NCI-H929 | *** | <.001 |
| siSEC24D_ AMO1 vs. siSEC24C&D_ AMO1 | ns | 0.237 |
| siSEC24D_ AMO1 vs. siSEC24C&D_ KMS-12PE | ns | 0.173 |
| siSEC24D_ AMO1 vs. siSEC24C&D_ NCI-H929 | *** | <.001 |
| siSEC24D_ KMS-12PE vs. siSEC24D_ NCI-H929 | *** | <.001 |
| siSEC24D_ KMS-12PE vs. siSEC24A&B_ AMO1 | *** | <.001 |
| siSEC24D_ KMS-12PE vs. siSEC24A&B_ KMS-12PE | ns | 0.103 |
| siSEC24D_ KMS-12PE vs. siSEC24A&B_ NCI-H929 | *** | <.001 |
| siSEC24D_ KMS-12PE vs. siSEC24A&C_ AMO1 | *** | <.001 |
| siSEC24D_ KMS-12PE vs. siSEC24A&C_ KMS-12PE | ns | 0.088 |
| siSEC24D_ KMS-12PE vs. siSEC24A&C_ NCI-H929 | *** | <.001 |
| siSEC24D_ KMS-12PE vs. siSEC24A&D_ AMO1 | *** | <.001 |
| siSEC24D_ KMS-12PE vs. siSEC24A&D_ KMS-12PE | ns | 0.071 |
| siSEC24D_ KMS-12PE vs. siSEC24A&D_ NCI-H929 | *** | <.001 |
| siSEC24D_ KMS-12PE vs. siSEC24B&C_ AMO1 | *** | <.001 |
| siSEC24D_ KMS-12PE vs. siSEC24B&C_ KMS-12PE | ns | 0.077 |
| siSEC24D_ KMS-12PE vs. siSEC24B&C_ NCI-H929 | *** | <.001 |
| siSEC24D_ KMS-12PE vs. siSEC24B&D_ AMO1 | ** | 0.002 |
| siSEC24D_ KMS-12PE vs. siSEC24B&D_ KMS-12PE | ns | 0.684 |
| siSEC24D_ KMS-12PE vs. siSEC24B&D_ NCI-H929 | *** | <.001 |
| siSEC24D_ KMS-12PE vs. siSEC24C&D_ AMO1 | ** | 0.005 |
| siSEC24D_ KMS-12PE vs. siSEC24C&D_ KMS-12PE | ** | 0.006 |
| siSEC24D_ KMS-12PE vs. siSEC24C&D_ NCI-H929 | *** | <.001 |
| siSEC24D_ NCI-H929 vs. siSEC24A&B_ AMO1 | *** | <.001 |
| siSEC24D_ NCI-H929 vs. siSEC24A&B_ KMS-12PE | *** | <.001 |
| siSEC24D_ NCI-H929 vs. siSEC24A&B_ NCI-H929 | *** | <.001 |
| siSEC24D_ NCI-H929 vs. siSEC24A&C_ AMO1 | ns | 0.795 |
| siSEC24D_ NCI-H929 vs. siSEC24A&C_ KMS-12PE | *** | <.001 |
| siSEC24D_ NCI-H929 vs. siSEC24A&C_ NCI-H929 | ns | 0.832 |
| siSEC24D_ NCI-H929 vs. siSEC24A&D_ AMO1 | ns | 0.791 |
| siSEC24D_ NCI-H929 vs. siSEC24A&D_ KMS-12PE | *** | <.001 |
| siSEC24D_ NCI-H929 vs. siSEC24A&D_ NCI-H929 | ns | 0.232 |
| siSEC24D_ NCI-H929 vs. siSEC24B&C_ AMO1 | ns | 0.527 |
| siSEC24D_ NCI-H929 vs. siSEC24B&C_ KMS-12PE | *** | <.001 |
| siSEC24D_ NCI-H929 vs. siSEC24B&C_ NCI-H929 | ns | 0.802 |
| siSEC24D_ NCI-H929 vs. siSEC24B&D_ AMO1 | ** | 0.005 |
| siSEC24D_ NCI-H929 vs. siSEC24B&D_ KMS-12PE | *** | <.001 |
| siSEC24D_ NCI-H929 vs. siSEC24B&D_ NCI-H929 | ns | 0.109 |
| siSEC24D_ NCI-H929 vs. siSEC24C&D_ AMO1 | ** | 0.001 |
| siSEC24D_ NCI-H929 vs. siSEC24C&D_ KMS-12PE | * | 0.017 |
| siSEC24D_ NCI-H929 vs. siSEC24C&D_ NCI-H929 | ** | 0.008 |
| siSEC24A&B_ AMO1 vs. siSEC24A&B_ KMS-12PE | *** | <.001 |
| siSEC24A&B_ AMO1 vs. siSEC24A&B_ NCI-H929 | ns | 0.469 |
| siSEC24A&B_ AMO1 vs. siSEC24A&C_ AMO1 | *** | <.001 |
| siSEC24A&B_ AMO1 vs. siSEC24A&C_ KMS-12PE | *** | <.001 |
| siSEC24A&B_ AMO1 vs. siSEC24A&C_ NCI-H929 | ** | 0.001 |
| siSEC24A&B_ AMO1 vs. siSEC24A&D_ AMO1 | *** | <.001 |
| siSEC24A&B_ AMO1 vs. siSEC24A&D_ KMS-12PE | *** | <.001 |
| siSEC24A&B_ AMO1 vs. siSEC24A&D_ NCI-H929 | * | 0.025 |
| siSEC24A&B_ AMO1 vs. siSEC24B&C_ AMO1 | *** | <.001 |
| siSEC24A&B_ AMO1 vs. siSEC24B&C_ KMS-12PE | *** | <.001 |
| siSEC24A&B_ AMO1 vs. siSEC24B&C_ NCI-H929 | ** | 0.001 |
| siSEC24A&B_ AMO1 vs. siSEC24B&D_ AMO1 | *** | <.001 |
| siSEC24A&B_ AMO1 vs. siSEC24B&D_ KMS-12PE | *** | <.001 |
| siSEC24A&B_ AMO1 vs. siSEC24B&D_ NCI-H929 | ns | 0.071 |
| siSEC24A&B_ AMO1 vs. siSEC24C&D_ AMO1 | *** | <.001 |
| siSEC24A&B_ AMO1 vs. siSEC24C&D_ KMS-12PE | *** | <.001 |
| siSEC24A&B_ AMO1 vs. siSEC24C&D_ NCI-H929 | ns | 0.71 |
| siSEC24A&B_ KMS-12PE vs. siSEC24A&B_ NCI-H929 | *** | <.001 |
| siSEC24A&B_ KMS-12PE vs. siSEC24A&C_ AMO1 | *** | <.001 |
| siSEC24A&B_ KMS-12PE vs. siSEC24A&C_ KMS-12PE | ns | 0.94 |
| siSEC24A&B_ KMS-12PE vs. siSEC24A&C_ NCI-H929 | *** | <.001 |
| siSEC24A&B_ KMS-12PE vs. siSEC24A&D_ AMO1 | *** | <.001 |
| siSEC24A&B_ KMS-12PE vs. siSEC24A&D_ KMS-12PE | ns | 0.861 |
| siSEC24A&B_ KMS-12PE vs. siSEC24A&D_ NCI-H929 | *** | <.001 |
| siSEC24A&B_ KMS-12PE vs. siSEC24B&C_ AMO1 | *** | <.001 |
| siSEC24A&B_ KMS-12PE vs. siSEC24B&C_ KMS-12PE | ns | 0.888 |
| siSEC24A&B_ KMS-12PE vs. siSEC24B&C_ NCI-H929 | *** | <.001 |
| siSEC24A&B_ KMS-12PE vs. siSEC24B&D_ AMO1 | ns | 0.182 |
| siSEC24A&B_ KMS-12PE vs. siSEC24B&D_ KMS-12PE | ns | 0.219 |
| siSEC24A&B_ KMS-12PE vs. siSEC24B&D_ NCI-H929 | *** | <.001 |
| siSEC24A&B_ KMS-12PE vs. siSEC24C&D_ AMO1 | ns | 0.335 |
| siSEC24A&B_ KMS-12PE vs. siSEC24C&D_ KMS-12PE | ns | 0.238 |
| siSEC24A&B_ KMS-12PE vs. siSEC24C&D_ NCI-H929 | *** | <.001 |
| siSEC24A&B_ NCI-H929 vs. siSEC24A&C_ AMO1 | *** | <.001 |
| siSEC24A&B_ NCI-H929 vs. siSEC24A&C_ KMS-12PE | *** | <.001 |
| siSEC24A&B_ NCI-H929 vs. siSEC24A&C_ NCI-H929 | *** | <.001 |
| siSEC24A&B_ NCI-H929 vs. siSEC24A&D_ AMO1 | *** | <.001 |
| siSEC24A&B_ NCI-H929 vs. siSEC24A&D_ KMS-12PE | *** | <.001 |
| siSEC24A&B_ NCI-H929 vs. siSEC24A&D_ NCI-H929 | ** | 0.007 |
| siSEC24A&B_ NCI-H929 vs. siSEC24B&C_ AMO1 | *** | <.001 |
| siSEC24A&B_ NCI-H929 vs. siSEC24B&C_ KMS-12PE | *** | <.001 |
| siSEC24A&B_ NCI-H929 vs. siSEC24B&C_ NCI-H929 | *** | <.001 |
| siSEC24A&B_ NCI-H929 vs. siSEC24B&D_ AMO1 | *** | <.001 |
| siSEC24A&B_ NCI-H929 vs. siSEC24B&D_ KMS-12PE | *** | <.001 |
| siSEC24A&B_ NCI-H929 vs. siSEC24B&D_ NCI-H929 | * | 0.022 |
| siSEC24A&B_ NCI-H929 vs. siSEC24C&D_ AMO1 | *** | <.001 |
| siSEC24A&B_ NCI-H929 vs. siSEC24C&D_ KMS-12PE | *** | <.001 |
| siSEC24A&B_ NCI-H929 vs. siSEC24C&D_ NCI-H929 | ns | 0.339 |
| siSEC24A&C_ AMO1 vs. siSEC24A&C_ KMS-12PE | *** | <.001 |
| siSEC24A&C_ AMO1 vs. siSEC24A&C_ NCI-H929 | ns | 0.623 |
| siSEC24A&C_ AMO1 vs. siSEC24A&D_ AMO1 | ns | 0.995 |
| siSEC24A&C_ AMO1 vs. siSEC24A&D_ KMS-12PE | *** | <.001 |
| siSEC24A&C_ AMO1 vs. siSEC24A&D_ NCI-H929 | ns | 0.117 |
| siSEC24A&C_ AMO1 vs. siSEC24B&C_ AMO1 | ns | 0.676 |
| siSEC24A&C_ AMO1 vs. siSEC24B&C_ KMS-12PE | *** | <.001 |
| siSEC24A&C_ AMO1 vs. siSEC24B&C_ NCI-H929 | ns | 0.593 |
| siSEC24A&C_ AMO1 vs. siSEC24B&D_ AMO1 | ** | 0.004 |
| siSEC24A&C_ AMO1 vs. siSEC24B&D_ KMS-12PE | *** | <.001 |
| siSEC24A&C_ AMO1 vs. siSEC24B&D_ NCI-H929 | * | 0.045 |
| siSEC24A&C_ AMO1 vs. siSEC24C&D_ AMO1 | *** | <.001 |
| siSEC24A&C_ AMO1 vs. siSEC24C&D_ KMS-12PE | * | 0.019 |
| siSEC24A&C_ AMO1 vs. siSEC24C&D_ NCI-H929 | ** | 0.002 |
| siSEC24A&C_ KMS-12PE vs. siSEC24A&C_ NCI-H929 | *** | <.001 |
| siSEC24A&C_ KMS-12PE vs. siSEC24A&D_ AMO1 | *** | <.001 |
| siSEC24A&C_ KMS-12PE vs. siSEC24A&D_ KMS-12PE | ns | 0.921 |
| siSEC24A&C_ KMS-12PE vs. siSEC24A&D_ NCI-H929 | *** | <.001 |
| siSEC24A&C_ KMS-12PE vs. siSEC24B&C_ AMO1 | ** | 0.001 |
| siSEC24A&C_ KMS-12PE vs. siSEC24B&C_ KMS-12PE | ns | 0.948 |
| siSEC24A&C_ KMS-12PE vs. siSEC24B&C_ NCI-H929 | *** | <.001 |
| siSEC24A&C_ KMS-12PE vs. siSEC24B&D_ AMO1 | ns | 0.211 |
| siSEC24A&C_ KMS-12PE vs. siSEC24B&D_ KMS-12PE | ns | 0.192 |
| siSEC24A&C_ KMS-12PE vs. siSEC24B&D_ NCI-H929 | *** | <.001 |
| siSEC24A&C_ KMS-12PE vs. siSEC24C&D_ AMO1 | ns | 0.38 |
| siSEC24A&C_ KMS-12PE vs. siSEC24C&D_ KMS-12PE | ns | 0.27 |
| siSEC24A&C_ KMS-12PE vs. siSEC24C&D_ NCI-H929 | *** | <.001 |
| siSEC24A&C_ NCI-H929 vs. siSEC24A&D_ AMO1 | ns | 0.619 |
| siSEC24A&C_ NCI-H929 vs. siSEC24A&D_ KMS-12PE | *** | <.001 |
| siSEC24A&C_ NCI-H929 vs. siSEC24A&D_ NCI-H929 | ns | 0.324 |
| siSEC24A&C_ NCI-H929 vs. siSEC24B&C_ AMO1 | ns | 0.387 |
| siSEC24A&C_ NCI-H929 vs. siSEC24B&C_ KMS-12PE | *** | <.001 |
| siSEC24A&C_ NCI-H929 vs. siSEC24B&C_ NCI-H929 | ns | 0.968 |
| siSEC24A&C_ NCI-H929 vs. siSEC24B&D_ AMO1 | ** | 0.002 |
| siSEC24A&C_ NCI-H929 vs. siSEC24B&D_ KMS-12PE | *** | <.001 |
| siSEC24A&C_ NCI-H929 vs. siSEC24B&D_ NCI-H929 | ns | 0.163 |
| siSEC24A&C_ NCI-H929 vs. siSEC24C&D_ AMO1 | *** | <.001 |
| siSEC24A&C_ NCI-H929 vs. siSEC24C&D_ KMS-12PE | * | 0.01 |
| siSEC24A&C_ NCI-H929 vs. siSEC24C&D_ NCI-H929 | * | 0.014 |
| siSEC24A&D_ AMO1 vs. siSEC24A&D_ KMS-12PE | *** | <.001 |
| siSEC24A&D_ AMO1 vs. siSEC24A&D_ NCI-H929 | ns | 0.116 |
| siSEC24A&D_ AMO1 vs. siSEC24B&C_ AMO1 | ns | 0.68 |
| siSEC24A&D_ AMO1 vs. siSEC24B&C_ KMS-12PE | *** | <.001 |
| siSEC24A&D_ AMO1 vs. siSEC24B&C_ NCI-H929 | ns | 0.589 |
| siSEC24A&D_ AMO1 vs. siSEC24B&D_ AMO1 | ** | 0.004 |
| siSEC24A&D_ AMO1 vs. siSEC24B&D_ KMS-12PE | *** | <.001 |
| siSEC24A&D_ AMO1 vs. siSEC24B&D_ NCI-H929 | * | 0.044 |
| siSEC24A&D_ AMO1 vs. siSEC24C&D_ AMO1 | *** | <.001 |
| siSEC24A&D_ AMO1 vs. siSEC24C&D_ KMS-12PE | * | 0.02 |
| siSEC24A&D_ AMO1 vs. siSEC24C&D_ NCI-H929 | ** | 0.002 |
| siSEC24A&D_ KMS-12PE vs. siSEC24A&D_ NCI-H929 | *** | <.001 |
| siSEC24A&D_ KMS-12PE vs. siSEC24B&C_ AMO1 | ** | 0.002 |
| siSEC24A&D_ KMS-12PE vs. siSEC24B&C_ KMS-12PE | ns | 0.973 |
| siSEC24A&D_ KMS-12PE vs. siSEC24B&C_ NCI-H929 | *** | <.001 |
| siSEC24A&D_ KMS-12PE vs. siSEC24B&D_ AMO1 | ns | 0.256 |
| siSEC24A&D_ KMS-12PE vs. siSEC24B&D_ KMS-12PE | ns | 0.161 |
| siSEC24A&D_ KMS-12PE vs. siSEC24B&D_ NCI-H929 | *** | <.001 |
| siSEC24A&D_ KMS-12PE vs. siSEC24C&D_ AMO1 | ns | 0.446 |
| siSEC24A&D_ KMS-12PE vs. siSEC24C&D_ KMS-12PE | ns | 0.315 |
| siSEC24A&D_ KMS-12PE vs. siSEC24C&D_ NCI-H929 | *** | <.001 |
| siSEC24A&D_ NCI-H929 vs. siSEC24B&C_ AMO1 | ns | 0.053 |
| siSEC24A&D_ NCI-H929 vs. siSEC24B&C_ KMS-12PE | *** | <.001 |
| siSEC24A&D_ NCI-H929 vs. siSEC24B&C_ NCI-H929 | ns | 0.344 |
| siSEC24A&D_ NCI-H929 vs. siSEC24B&D_ AMO1 | *** | <.001 |
| siSEC24A&D_ NCI-H929 vs. siSEC24B&D_ KMS-12PE | *** | <.001 |
| siSEC24A&D_ NCI-H929 vs. siSEC24B&D_ NCI-H929 | ns | 0.68 |
| siSEC24A&D_ NCI-H929 vs. siSEC24C&D_ AMO1 | *** | <.001 |
| siSEC24A&D_ NCI-H929 vs. siSEC24C&D_ KMS-12PE | *** | <.001 |
| siSEC24A&D_ NCI-H929 vs. siSEC24C&D_ NCI-H929 | ns | 0.117 |
| siSEC24B&C_ AMO1 vs. siSEC24B&C_ KMS-12PE | ** | 0.002 |
| siSEC24B&C_ AMO1 vs. siSEC24B&C_ NCI-H929 | ns | 0.364 |
| siSEC24B&C_ AMO1 vs. siSEC24B&D_ AMO1 | * | 0.013 |
| siSEC24B&C_ AMO1 vs. siSEC24B&D_ KMS-12PE | *** | <.001 |
| siSEC24B&C_ AMO1 vs. siSEC24B&D_ NCI-H929 | * | 0.018 |
| siSEC24B&C_ AMO1 vs. siSEC24C&D_ AMO1 | ** | 0.004 |
| siSEC24B&C_ AMO1 vs. siSEC24C&D_ KMS-12PE | * | 0.045 |
| siSEC24B&C_ AMO1 vs. siSEC24C&D_ NCI-H929 | *** | <.001 |
| siSEC24B&C_ KMS-12PE vs. siSEC24B&C_ NCI-H929 | *** | <.001 |
| siSEC24B&C_ KMS-12PE vs. siSEC24B&D_ AMO1 | ns | 0.24 |
| siSEC24B&C_ KMS-12PE vs. siSEC24B&D_ KMS-12PE | ns | 0.171 |
| siSEC24B&C_ KMS-12PE vs. siSEC24B&D_ NCI-H929 | *** | <.001 |
| siSEC24B&C_ KMS-12PE vs. siSEC24C&D_ AMO1 | ns | 0.423 |
| siSEC24B&C_ KMS-12PE vs. siSEC24C&D_ KMS-12PE | ns | 0.299 |
| siSEC24B&C_ KMS-12PE vs. siSEC24C&D_ NCI-H929 | *** | <.001 |
| siSEC24B&C_ NCI-H929 vs. siSEC24B&D_ AMO1 | ** | 0.002 |
| siSEC24B&C_ NCI-H929 vs. siSEC24B&D_ KMS-12PE | *** | <.001 |
| siSEC24B&C_ NCI-H929 vs. siSEC24B&D_ NCI-H929 | ns | 0.175 |
| siSEC24B&C_ NCI-H929 vs. siSEC24C&D_ AMO1 | *** | <.001 |
| siSEC24B&C_ NCI-H929 vs. siSEC24C&D_ KMS-12PE | ** | 0.009 |
| siSEC24B&C_ NCI-H929 vs. siSEC24C&D_ NCI-H929 | * | 0.015 |
| siSEC24B&D_ AMO1 vs. siSEC24B&D_ KMS-12PE | ** | 0.007 |
| siSEC24B&D_ AMO1 vs. siSEC24B&D_ NCI-H929 | *** | <.001 |
| siSEC24B&D_ AMO1 vs. siSEC24C&D_ AMO1 | ns | 0.646 |
| siSEC24B&D_ AMO1 vs. siSEC24C&D_ KMS-12PE | ns | 0.981 |
| siSEC24B&D_ AMO1 vs. siSEC24C&D_ NCI-H929 | *** | <.001 |
| siSEC24B&D_ KMS-12PE vs. siSEC24B&D_ NCI-H929 | *** | <.001 |
| siSEC24B&D_ KMS-12PE vs. siSEC24C&D_ AMO1 | * | 0.018 |
| siSEC24B&D_ KMS-12PE vs. siSEC24C&D_ KMS-12PE | * | 0.017 |
| siSEC24B&D_ KMS-12PE vs. siSEC24C&D_ NCI-H929 | *** | <.001 |
| siSEC24B&D_ NCI-H929 vs. siSEC24C&D_ AMO1 | *** | <.001 |
| siSEC24B&D_ NCI-H929 vs. siSEC24C&D_ KMS-12PE | *** | <.001 |
| siSEC24B&D_ NCI-H929 vs. siSEC24C&D_ NCI-H929 | ns | 0.233 |
| siSEC24C&D_ AMO1 vs. siSEC24C&D_ KMS-12PE | ns | 0.69 |
| siSEC24C&D_ AMO1 vs. siSEC24C&D_ NCI-H929 | *** | <.001 |
| siSEC24C&D_ KMS-12PE vs. siSEC24C&D_ NCI-H929 | *** | <.001 |

**Results of statistical analyses for Figure 2a, b**

| **1way ANOVA Comparisons of AMO-1** | **Summary** | **P Value** |
| --- | --- | --- |
| siCtrl vs. siSEC24A | *** | 0.0008 |
| siCtrl vs. siSEC24B | *** | <0.0001 |
| siCtrl vs. siSEC24A&B | *** | <0.0001 |
| siSEC24A&B vs. siSEC24A | * | .042 |
| siSEC24A&B vs. siSEC24B | ns | .818 |
| siSEC24A vs. siSEC24B | * | .015 |
|  |  |  |
| **1way ANOVA Comparisons of NCI-H929** | **Summary** | **P Value** |
| siCtrl vs. siSEC24A | * | 0.0410 |
| siCtrl vs. siSEC24B | ** | 0.0063 |
| siCtrl vs. siSEC24A&B | ** | 0.0092 |
| siSEC24A&B vs. siSEC24A | ns | .220 |
| siSEC24A&B vs. siSEC24B | ns | .854 |
| siSEC24A vs. siSEC24B | ns | .113 |

**Results of statistical analyses for Supplemental Figure 3**

| **1way ANOVA Comparisons_ERES# per AMO-1 cell** | **Summary** | **P Value** |
| --- | --- | --- |
| siCtrl vs. siSEC24A | ns | 0.999 |
| siCtrl vs. siSEC24B | ns | >.999 |
| siCtrl vs. siSEC24C | ns | 0.632 |
| siCtrl vs. siSEC24D | ns | 0.51 |
| siCtrl vs. siSEC24A&B | ns | >.999 |
| siCtrl vs. siSEC24A&C | ns | 0.89 |
| siCtrl vs. siSEC24A&D | ns | 0.937 |
| siCtrl vs. siSEC24B&C | ns | 0.754 |
| siCtrl vs. siSEC24B&D | ns | >.999 |
| siCtrl vs. siSEC24C&D | ns | 0.736 |
| siCtrl vs. siSEC16A&B | *** | <.001 |
| siSEC24A vs. siSEC24B | ns | >.999 |
| siSEC24A vs. siSEC24C | ns | 0.994 |
| siSEC24A vs. siSEC24D | ns | 0.974 |
| siSEC24A vs. siSEC24A&B | ns | 0.998 |
| siSEC24A vs. siSEC24A&C | ns | >.999 |
| siSEC24A vs. siSEC24A&D | ns | >.999 |
| siSEC24A vs. siSEC24B&C | ns | >.999 |
| siSEC24A vs. siSEC24B&D | ns | 0.984 |
| siSEC24A vs. siSEC24C&D | ns | >.999 |
| siSEC24A vs. siSEC16A&B | *** | <.001 |
| siSEC24B vs. siSEC24C | ns | 0.686 |
| siSEC24B vs. siSEC24D | ns | 0.557 |
| siSEC24B vs. siSEC24A&B | ns | >.999 |
| siSEC24B vs. siSEC24A&C | ns | 0.934 |
| siSEC24B vs. siSEC24A&D | ns | 0.972 |
| siSEC24B vs. siSEC24B&C | ns | 0.805 |
| siSEC24B vs. siSEC24B&D | ns | >.999 |
| siSEC24B vs. siSEC24C&D | ns | 0.782 |
| siSEC24B vs. siSEC16A&B | *** | <.001 |
| siSEC24C vs. siSEC24D | ns | >.999 |
| siSEC24C vs. siSEC24A&B | ns | 0.359 |
| siSEC24C vs. siSEC24A&C | ns | >.999 |
| siSEC24C vs. siSEC24A&D | ns | >.999 |
| siSEC24C vs. siSEC24B&C | ns | >.999 |
| siSEC24C vs. siSEC24B&D | ns | 0.193 |
| siSEC24C vs. siSEC24C&D | ns | >.999 |
| siSEC24C vs. siSEC16A&B | *** | <.001 |
| siSEC24D vs. siSEC24A&B | ns | 0.27 |
| siSEC24D vs. siSEC24A&C | ns | 0.996 |
| siSEC24D vs. siSEC24A&D | ns | 0.996 |
| siSEC24D vs. siSEC24B&C | ns | >.999 |
| siSEC24D vs. siSEC24B&D | ns | 0.142 |
| siSEC24D vs. siSEC24C&D | ns | >.999 |
| siSEC24D vs. siSEC16A&B | *** | <.001 |
| siSEC24A&B vs. siSEC24A&C | ns | 0.662 |
| siSEC24A&B vs. siSEC24A&D | ns | 0.819 |
| siSEC24A&B vs. siSEC24B&C | ns | 0.443 |
| siSEC24A&B vs. siSEC24B&D | ns | >.999 |
| siSEC24A&B vs. siSEC24C&D | ns | 0.399 |
| siSEC24A&B vs. siSEC16A&B | *** | <.001 |
| siSEC24A&C vs. siSEC24A&D | ns | >.999 |
| siSEC24A&C vs. siSEC24B&C | ns | >.999 |
| siSEC24A&C vs. siSEC24B&D | ns | 0.406 |
| siSEC24A&C vs. siSEC24C&D | ns | >.999 |
| siSEC24A&C vs. siSEC16A&B | *** | <.001 |
| siSEC24A&D vs. siSEC24B&C | ns | >.999 |
| siSEC24A&D vs. siSEC24B&D | ns | 0.596 |
| siSEC24A&D vs. siSEC24C&D | ns | >.999 |
| siSEC24A&D vs. siSEC16A&B | *** | <.001 |
| siSEC24B&C vs. siSEC24B&D | ns | 0.237 |
| siSEC24B&C vs. siSEC24C&D | ns | >.999 |
| siSEC24B&C vs. siSEC16A&B | *** | <.001 |
| siSEC24B&D vs. siSEC24C&D | ns | 0.203 |
| siSEC24B&D vs. siSEC16A&B | *** | <.001 |
| siSEC24C&D vs. siSEC16A&B | *** | <.001 |
|  |  |  |
| **1way ANOVA Comparisons_ERES# per NCI-H929 cell** | **Summary** | **P Value** |
| siCtrl vs. siSEC24A | ns | 0.997 |
| siCtrl vs. siSEC24B | ns | 0.655 |
| siCtrl vs. siSEC24C | ns | 0.998 |
| siCtrl vs. siSEC24D | ns | 0.673 |
| siCtrl vs. siSEC24A&B | ns | 0.478 |
| siCtrl vs. siSEC24A&C | ns | >.999 |
| siCtrl vs. siSEC24A&D | ns | 0.556 |
| siCtrl vs. siSEC24B&C | ns | >.999 |
| siCtrl vs. siSEC24B&D | ns | 0.987 |
| siCtrl vs. siSEC24C&D | ns | >.999 |
| siCtrl vs. siSEC16A&B | *** | <.001 |
| siSEC24A vs. siSEC24B | ns | 0.987 |
| siSEC24A vs. siSEC24C | ns | 0.647 |
| siSEC24A vs. siSEC24D | ns | 0.984 |
| siSEC24A vs. siSEC24A&B | ns | 0.928 |
| siSEC24A vs. siSEC24A&C | ns | 0.968 |
| siSEC24A vs. siSEC24A&D | ns | 0.967 |
| siSEC24A vs. siSEC24B&C | ns | >.999 |
| siSEC24A vs. siSEC24B&D | ns | >.999 |
| siSEC24A vs. siSEC24C&D | ns | 0.86 |
| siSEC24A vs. siSEC16A&B | *** | <.001 |
| siSEC24B vs. siSEC24C | ns | 0.125 |
| siSEC24B vs. siSEC24D | ns | >.999 |
| siSEC24B vs. siSEC24A&B | ns | >.999 |
| siSEC24B vs. siSEC24A&C | ns | 0.431 |
| siSEC24B vs. siSEC24A&D | ns | >.999 |
| siSEC24B vs. siSEC24B&C | ns | 0.982 |
| siSEC24B vs. siSEC24B&D | ns | 0.998 |
| siSEC24B vs. siSEC24C&D | ns | 0.222 |
| siSEC24B vs. siSEC16A&B | *** | <.001 |
| siSEC24C vs. siSEC24D | ns | 0.156 |
| siSEC24C vs. siSEC24A&B | ns | 0.077 |
| siSEC24C vs. siSEC24A&C | ns | >.999 |
| siSEC24C vs. siSEC24A&D | ns | 0.091 |
| siSEC24C vs. siSEC24B&C | ns | 0.76 |
| siSEC24C vs. siSEC24B&D | ns | 0.544 |
| siSEC24C vs. siSEC24C&D | ns | >.999 |
| siSEC24C vs. siSEC16A&B | *** | <.001 |
| siSEC24D vs. siSEC24A&B | ns | >.999 |
| siSEC24D vs. siSEC24A&C | ns | 0.469 |
| siSEC24D vs. siSEC24A&D | ns | >.999 |
| siSEC24D vs. siSEC24B&C | ns | 0.978 |
| siSEC24D vs. siSEC24B&D | ns | 0.997 |
| siSEC24D vs. siSEC24C&D | ns | 0.274 |
| siSEC24D vs. siSEC16A&B | *** | <.001 |
| siSEC24A&B vs. siSEC24A&C | ns | 0.289 |
| siSEC24A&B vs. siSEC24A&D | ns | >.999 |
| siSEC24A&B vs. siSEC24B&C | ns | 0.914 |
| siSEC24A&B vs. siSEC24B&D | ns | 0.98 |
| siSEC24A&B vs. siSEC24C&D | ns | 0.141 |
| siSEC24A&B vs. siSEC16A&B | *** | <.001 |
| siSEC24A&C vs. siSEC24A&D | ns | 0.343 |
| siSEC24A&C vs. siSEC24B&C | ns | 0.987 |
| siSEC24A&C vs. siSEC24B&D | ns | 0.926 |
| siSEC24A&C vs. siSEC24C&D | ns | >.999 |
| siSEC24A&C vs. siSEC16A&B | *** | <.001 |
| siSEC24A&D vs. siSEC24B&C | ns | 0.957 |
| siSEC24A&D vs. siSEC24B&D | ns | 0.994 |
| siSEC24A&D vs. siSEC24C&D | ns | 0.164 |
| siSEC24A&D vs. siSEC16A&B | *** | <.001 |
| siSEC24B&C vs. siSEC24B&D | ns | >.999 |
| siSEC24B&C vs. siSEC24C&D | ns | 0.931 |
| siSEC24B&C vs. siSEC16A&B | *** | <.001 |
| siSEC24B&D vs. siSEC24C&D | ns | 0.771 |
| siSEC24B&D vs. siSEC16A&B | *** | <.001 |
| siSEC24C&D vs. siSEC16A&B | *** | <.001 |

**Results of statistical analyses for Supplementary Figure 10**

| **1way ANOVA Comparisons of KMS-12PE** | **Summary** | **P Value** |
| --- | --- | --- |
| siCtrl vs. siSEC24A&B | ns | 0.06 |
| siCtrl vs. siCtrl STF-083010 | ns | 0.89 |
| siCtrl vs. siSEC24A&B STF-083010 | ns | 0.351 |
| siCtrl vs. siCtrl KIRA6 | ns | 0.213 |
| siCtrl vs. siSEC24A&B KIRA6 | ns | 0.056 |
| siSECsiSEC24A&B vs. siCtrl STF-083010 | ** | 0.002 |
| siSECsiSEC24A&B vs. siSEC24A&B STF-083010 | *** | <.001 |
| siSECsiSEC24A&B vs. siCtrl KIRA6 | *** | <.001 |
| siSECsiSEC24A&B vs. siSEC24A&B KIRA6 | *** | <.001 |
| siCtrl STF-083010 vs. siSEC24A&B STF-083010 | ns | 0.973 |
| siCtrl STF-083010 vs. siCtrl KIRA6 | ns | 0.9 |
| siCtrl STF-083010 vs. siSEC24A&B KIRA6 | ns | 0.588 |
| siSEC24A&B STF-083010 vs. siCtrl KIRA6 | ns | >.999 |
| siSEC24A&B STF-083010 vs. siSEC24A&B KIRA6 | ns | 0.92 |
| siCtrl KIRA6 vs. siSEC24A&B KIRA6 | ns | 0.988 |
|  |  |  |
| **1way ANOVA Comparisons of OH-2** | **Summary** | **P Value** |
| siCtrl vs. siSEC24A&B | ns | .987 |
| siCtrl vs. siCtrl STF-083010 | ns | .331 |
| siCtrl vs. siSEC24A&B STF-083010 | ns | .910 |
| siCtrl vs. siCtrl KIRA6 | ns | .107 |
| siCtrl vs. siSEC24A&B KIRA6 | ns | >.999 |
| siSECsiSEC24A&B vs. siCtrl STF-083010 | ns | .071 |
| siSECsiSEC24A&B vs. siSEC24A&B STF-083010 | ns | .558 |
| siSECsiSEC24A&B vs. siCtrl KIRA6 | * | .016 |
| siSECsiSEC24A&B vs. siSEC24A&B KIRA6 | ns | .946 |
| siCtrl STF-083010 vs. siSEC24A&B STF-083010 | ns | .955 |
| siCtrl STF-083010 vs. siCtrl KIRA6 | ns | .977 |
| siCtrl STF-083010 vs. siSEC24A&B KIRA6 | ns | .469 |
| siSEC24A&B STF-083010 vs. siCtrl KIRA6 | ns | .684 |
| siSEC24A&B STF-083010 vs. siSEC24A&B KIRA6 | ns | .967 |
| siCtrl KIRA6 vs. siSEC24A&B KIRA6 | ns | .171 |
|  |  |  |
| **1way ANOVA Comparisons of AMO-1** | **Summary** | **P Value** |
| siCtrl vs. siSEC24A&B | *** | <.001 |
| siCtrl vs. siCtrl STF-083010 | ns | 0.156 |
| siCtrl vs. siSEC24A&B STF-083010 | * | 0.022 |
| siCtrl vs. siCtrl KIRA6 | ns | 0.584 |
| siCtrl vs. siSEC24A&B KIRA6 | *** | <.001 |
| siSEC24A&B vs. siCtrl STF-083010 | *** | <.001 |
| siSEC24A&B vs. siSEC24A&B STF-083010 | *** | <.001 |
| siSEC24A&B vs. siCtrl KIRA6 | *** | <.001 |
| siSEC24A&B vs. siSEC24A&B KIRA6 | ** | 0.005 |
| siCtrl STF-083010 vs. siSEC24A&B STF-083010 | *** | <.001 |
| siCtrl STF-083010 vs. siCtrl KIRA6 | ns | 0.991 |
| siCtrl STF-083010 vs. siSEC24A&B KIRA6 | *** | <.001 |
| siSEC24A&B STF-083010 vs. siCtrl KIRA6 | *** | <.001 |
| siSEC24A&B STF-083010 vs. siSEC24A&B KIRA6 | ns | 0.288 |
| siCtrl KIRA6 vs. siSEC24A&B KIRA6 | *** | <.001 |
|  |  |  |
| **1way ANOVA Comparisons of FOLE** | **Summary** | **P Value** |
| siCtrl vs. siCtrl STF-083010 | ns | .469 |
| siCtrl vs. siCtrl KIRA6 | ns | .387 |
| siCtrl vs. siSEC24A&B | *** | <.001 |
| siCtrl vs. siSEC24A&B STF-083010 | ns | .974 |
| siCtrl vs. siSEC24A&B KIRA6 | ns | .998 |
| siCtrl STF-083010 vs. siCtrl KIRA6 | ns | >.999 |
| siCtrl STF-083010 vs. siSEC24A&B | *** | <.001 |
| siCtrl STF-083010 vs. siSEC24A&B STF-083010 | ns | .093 |
| siCtrl STF-083010 vs. siSEC24A&B KIRA6 | ns | .732 |
| siCtrl KIRA6 vs. siSEC24A&B | *** | <.001 |
| siCtrl KIRA6 vs. siSEC24A&B STF-083010 | ns | .065 |
| siCtrl KIRA6 vs. siSEC24A&B KIRA6 | ns | .652 |
| siSEC24A&B vs. siSEC24A&B STF-083010 | *** | <.001 |
| siSEC24A&B vs. siSEC24A&B KIRA6 | *** | <.001 |
| siSEC24A&B STF-083010 vs. siSEC24A&B KIRA6 | ns | .832 |
|  |  |  |
| **1way ANOVA Comparisons of NCI-H929** | **Summary** | **P Value** |
| siCtrl vs. siSEC24A&B | *** | <.001 |
| siCtrl vs. siCtrl STF-083010 | * | 0.026 |
| siCtrl vs. siSEC24A&B STF-083010 | ns | 0.319 |
| siCtrl vs. siCtrl KIRA6 | *** | <.001 |
| siCtrl vs. siSEC24A&B KIRA6 | *** | <.001 |
| siSEC24A&B vs. siCtrl STF-083010 | *** | <.001 |
| siSEC24A&B vs. siSEC24A&B STF-083010 | *** | <.001 |
| siSEC24A&B vs. siCtrl KIRA6 | *** | <.001 |
| siSEC24A&B vs. siSEC24A&B KIRA6 | *** | <.001 |
| siCtrl STF-083010 vs. siSEC24A&B STF-083010 | ns | 0.927 |
| siCtrl STF-083010 vs. siCtrl KIRA6 | ns | 0.357 |
| siCtrl STF-083010 vs. siSEC24A&B KIRA6 | ns | 0.251 |
| siSEC24A&B STF-083010 vs. siCtrl KIRA6 | * | 0.042 |
| siSEC24A&B STF-083010 vs. siSEC24A&B KIRA6 | * | 0.024 |
| siCtrl KIRA6 vs. siSEC24A&B KIRA6 | ns | >.999 |
